# Structural features of many circular and leaderless bacteriocins are similar to those in saposins and saposin-like peptides†
†The authors declare no competing interests.
‡
‡Electronic supplementary information (ESI) available. See DOI: 10.1039/c6md00607h


**DOI:** 10.1039/c6md00607h

**Published:** 2017-01-11

**Authors:** K. M. Towle, J. C. Vederas

**Affiliations:** a Department of Chemistry , University of Alberta , Edmonton , Alberta , T6G 2G2 Canada . Email: john.vederas@ualberta.ca

## Abstract

Bacteriocins are potent antimicrobial peptides that are ribosomally produced and exported by bacteria, presumably to aid elimination of competing microorganisms. Many circular and linear leaderless bacteriocins have a recuring three dimensional structural motif known as a saposin-like fold. Although these bacteriocin sizes and sequences are often quite different, and their mechanisms of action vary, this conserved motif of multiple helices appears critical for activity and may enable peptide–lipid and peptide–receptor interactions in target bacterial cell membranes. Comparisons between electrostatic surfaces and hydrophobic surface maps of different bacteriocins are discussed emphasizing similarities and differences in the context of proposed modes of action.

## Abstract 2

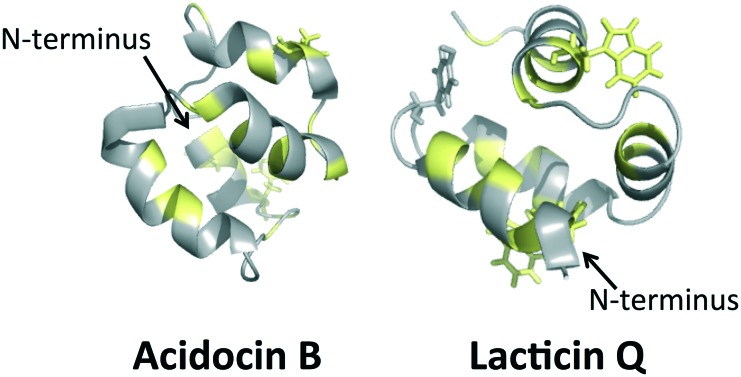
Bacteriocins are potent antimicrobial peptides that are ribosomally produced and exported by bacteria, presumably to aid elimination of competing microorganisms.

## Introduction

Antibiotics are of great importance in modern healthcare systems. They not only allow treatment of routine infections, but also enable advanced medical procedures, such as invasive surgeries and immunosuppression for cancer treatment. The global emergence of multi-drug resistant infections threatens the ability to practice medicine using techniques that are common today.^1^ The death of 700 000 people each year from drug resistant infections emphasizes the need for new antimicrobials.^1^ Antimicrobial peptides, both synthetic and natural, are emerging as an attractive approach for anti-infective therapy due to their ability to disrupt the bacterial cell membranes. This can occur either through a receptor mediated or receptor independent fashion.^2^ Peptides that act in a receptor independent fashion have been suggested to reduce the chance of resistance forming in the bacteria,^3^ but they are often much less active than natural compounds that require a receptor molecule in the target microorganism.

Ribosomally synthesized peptides produced by bacteria that possess antimicrobial activities are known as bacteriocins.^4^,^5^ The bacteriocins act as a defence mechanism against competing bacteria, and therefore their activity is sometimes limited to strains of bacteria closely related to the producing organism.^6^ However, many bacteriocins have been shown to exhibit broad-spectrum activity against Gram-positive organisms, and in some cases can also act against Gram-negative bacteria if the outer membrane is disrupted.^5^

It is accepted that the structure and function of peptides and proteins are deeply intertwined. Some researchers suggest that the structures of amino acid polymers are more highly conserved than their sequences, and several protein and peptide search programs now offer structural similarity searches to find common three dimensional motifs between otherwise unrelated peptides.^7^,^8^ It is presently unclear whether natural peptides with similar structure descend from the same phylogenic tree or if nature simply reuses the same motifs in protein folding. Diverse but recurring structural themes include TIM-barrels, trefoils, lassos and G-coupled protein receptors along with many others.^8^–^12^ As more three dimensional structures become available, it is apparent that one common motif among certain bacteriocin classes is the saposin-like fold. Herein we focus on the relationship between circular and linear leaderless bacteriocins whose backbone structure assumes a saposin-like fold or a related α-helical bundle in the context of their possible mechanisms and their interactions with lipids.

### Peptide–lipid interactions

The interaction of peptides and lipids has been studied extensively since Singer and Nicolson first proposed the fluid mosaic model of biological membranes.^13^,^14^ There are many different ways that peptides interact with lipids, and membrane proteins are often classified as integral membrane proteins or peripheral membrane proteins.^15^ Integral membrane proteins are permanently attached to the biological membrane.^15^ A few examples include transport proteins that allow movement of ions or molecules across a membrane, as well as integrins and G protein-coupled receptors, both of which act as signalling molecules. Peripheral membrane proteins are those that temporarily bind to the surface of the membrane, either through direct contact with the lipids themselves (receptor independent) or through other surface-bound targets (receptor dependent).^15^ Phospholipases, which bind to zwitterionic lipids, and G-proteins, which bind to G protein-coupled receptors, are a few examples of peripheral membrane proteins.^15^ Antimicrobial peptides typically fall into the category of peripheral membrane proteins based on their propensity to modify membrane properties but still be readily dissociated. Typically, the antimicrobial peptides bind to the surface of the membrane, followed by disruption of the membrane through a variety of mechanisms such as the carpet model, pore formation or the barrel-stave mechanism.^15^,^16^

Antimicrobial peptides (AMPs) are isolated from a broad range of eukaryotic and prokaryotic sources. AMPs differ widely in three dimensional structure and mechanism of action.^16^ Some, such as magainin-2 from frogs and the leaderless bacteriocin LsbB, exist predominantly as a single α-helix,^17^,^18^ whereas others, such as human alpha defensin 5 and leaderless bacteriocin laterosporulin, consist of a series of β-sheets.^19^–^21^ Yet others contain a mixture of both α-helix and β-sheets, such as leucocin A.^22^ Although certain AMPs, such as magainin, require no receptor and disrupt membranes directly as either l or d-enantiomers, many others require recognition of a chiral target molecule in the membrane and are only fully active as natural l-enantiomers.^6^,^23^ The bacteriocins that contain α-helices in a saposin-like fold or α-helical bundle vary in sequence, size, overall charge and possible receptor targets, but all are peripheral membrane binders that disrupt the lipid bilayer in their target organisms.

## Saposins and saposin-like peptides (SAPLIP)

**Fig. 1:**
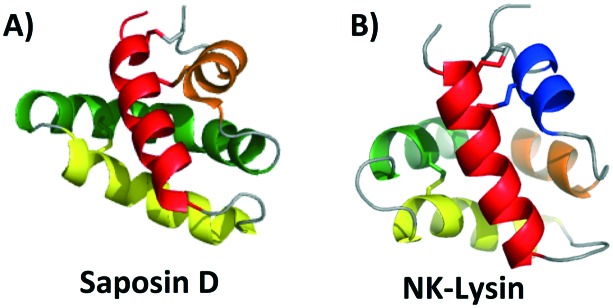
Cartoon representation of A) saposin D and B) NK-lysin created with PyMOL. To depict directionality, each helix is colored a different color starting with the N-terminus, red; α-helix 1, yellow; α-helix 2, green; α-helix 3 and orange; α-helix 4. In the case that there is a 5th α-helix it is colored blue.

Saposins are a group of four proteins derived from a single, larger precursor protein produced in humans, prosaposin, and are involved in sphingolipid catabolisim within the lysosome.^24^ These four proteins (saposins A–D) act as activator proteins, modifying the environment around lipids creating an opportunity for specific enzymes to reach the breakable bonds within the lipids.^25^ The interaction of these proteins with lipids is thought to occur through formation of oligomers that allow for lipid binding.^26^–^28^ Herein comparisons will be made to the structure of saposin D, which is known to preferentially bind anionic lipids, not unlike many bacteriocins that interact with negatively charged cell membranes.^29^,^30^ The saposin fold is comprised of 4 or 5 α-helices. These helices are packed in two “leaves” in such a way that a ‘v-shape’ is formed between α-helix 2 and α-helix 3 as well as α-helix 4 and α-helix 5. In addition, α-helix 1 is packed in such a way that it is nearly perpendicular to α-helices 2 and 3 (Fig. 1A).^31^ In the saposin fold, disulfide bonds occur between helices that add stability to this structural motif.^31^

Interestingly, the monomeric structural motif characteristic of the saposins is found in a larger superfamily of proteins, termed as saposin-like peptides (SAPLIPs). These peptides (*e.g.* NK-lysin)^32^ share structural similarities, and in most cases have three disulfide bonds that are formed between helices to add stability to the three dimensional structure (Fig. 1B). Many of these SAPLIPs also form dimers in their active state. In the case of human lung surfactant protein B (SP-B), a seventh cysteine forms an intermolecular disulfide bond to stabilize the active dimer.^31^ Other SAPLIPs rely on hydrophobic or electrostatic interactions to stabilize the formation of the dimer. Recent studies suggest that a transition in structure may occur upon interaction of these peptides with the cell membrane. Specifically, it could be that SAPLIPs, which are initially a small, compact, monomeric globular fold (saposin fold), shift to a more open dimeric shell upon binding. It is possible that this transition is an underlying general principle for members of this superfamily.^33^

Unexpectedly, this saposin fold is similar to a common structural motif in some bacteriocins discussed below. Termed the saposin-like *fold* (as opposed to saposin-like *peptide* (SAPLIP)), this structural motif is comprised of two α-helices that form a ‘v-shape’ and an additional helix that is nearly perpendicular to this ‘v-shape’. Unlike the saposin-fold, there are no disulfide bonds present to stabilize this saposin-like fold in the bacteriocins.

## Bacteriocins with a saposin-like fold or α-helical bundle

Traditionally, bacteriocins have been grouped according to a class system first introduced by Klaenhammer that was later refined and updated to include a multitude of modified peptides.^5^,^34^ It has been suggested that antimicrobial peptides, including bacteriocins, can be grouped by common structural motifs.^35^ Some recent studies suggest that common physical and functional properties among some AMPs may be partly due to the similar structural motifs shared amongst them.^35^

**Fig. 2:**
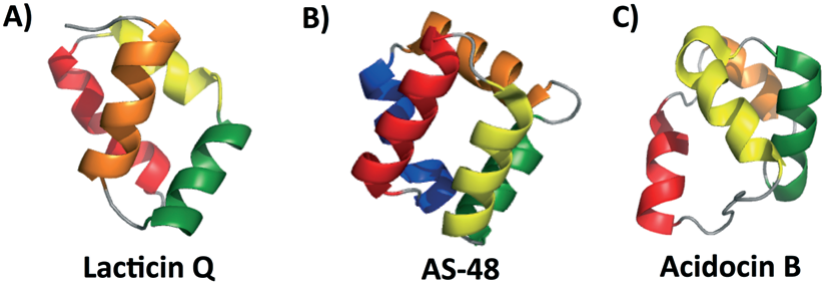
Cartoon representations of A) a leaderless bacteriocin lacticin Q, B) subgroup i circular bacteriocin AS-48 and C) subgroup ii circular bacteriocin acidocin B created with PyMOL. To depict directionality, each helix is colored a different color starting with the N-terminus, red; α-helix 1, yellow; α-helix 2, green; α-helix 3 and orange; α-helix 4. In the case that there is a 5th α-helix, it is colored blue.

The special structural features found in the saposins and SAPLIPs were first recognized in a circular bacteriocin, enterocin AS-48.^36^ Since then, the structures of many more bacteriocins have been solved or modelled. Analysis of these structures reveals that the saposin-like fold or a related α-helical bundle appears to be a conserved structural motif among some groups of bacteriocins. In particular, many leaderless bacteriocins and most circular bacteriocins possess such structures (Fig. 2). All of these are postulated to interact with bacterial membranes, emphasizing that the presence of this structural theme is a key feature for their interaction with lipids.^36^–^40^ As mentioned above, in contrast to the saposins and SAPLIPs, which contain conserved cysteines that participate in disulfide formation between helices, bacteriocins whose structure takes on a saposin-like fold or α-helical bundle do not contain any cysteines.

### Circular bacteriocins

Circular bacteriocins are ribosomally synthesized antimicrobial peptides in which the N and C termini are post-translationally linked through a peptide bond. These bacteriocins tend to have broad-spectrum activity against Gram-positive bacteria, and some have even been shown to have activity against Gram-negative bacteria if the outer membrane is disrupted with EDTA.^6^ To date, a dozen circular bacteriocins have been isolated and characterized (Table 1).^41^,^42^ Of these, the circular bacteriocins can be subdivided into two groups. Subgroub i is categorized by having an overall cationic surface, whereas the subgroup ii lacks such basic residues, resulting in low isoelectric point and overall anionic surface.^42^ The well characterized enterocin AS-48, carnocyclin A, NKR-5-3B all belong to this first subgroup i along with circularin A, uberolysin, garvicin ML, amylocyclin, leucocyclicin and lactocyclicin Q.^36^,^40^,^43^–^49^ The second subgroup ii includes the peptides acidocin B, gassericin A and butyrivibriocin AR10.^41^,^50^,^51^ The structures of subgroup ii, though primarily α-helical, do not assume the saposin-like fold, but rather a related four α-helix bundle.^41^ Subtilosin A, a circular sactipeptide with three cysteine sulfur to α-carbon linkages, is a notable exception and does not fit into either subgroup i or subgroup ii.^52^

**Table 1:** List of circular and leaderless bacteriocins that adopt a saposin-like fold or a helical-bundle

Bacteriocin	Producing organism	Circular (C) or leaderless (L) bacteriocin	No. of residues	Net charge at pH 7	Activity spectrum	Secondary structural motif	Reference
Circularin A^*a*^	*Geobacillus kaustophilus*	C	76	+1	br. Gr.+	Saposin-like	44
Uberolysin^*a*^	*Streptococcus uberis*	C	70	+3	br. Gr.+	Saposin-like	45
Enterocin AS-48^*b*^	*Enterocin feacalis*	C	70	+6	br. Gr.+	Saposin-like	36
NKR-5-3B^*c*^	*Enterococcus faecalis*	C	64	+5	br. Gr.+	Saposin-like	40
Pneumocyclicin^*d*^	*Streptococcus pneumoniae*	C	64	+5	N/A	Saposin-like	74
Leucocyclicin Q^*d*^	*Leuconostoc mesenteroides*	C	63	+3	br. Gr.+	Saposin-like	48
Lactocyclicin Q^*a*^	*Lactococcus* sp.	C	61	+4	br. Gr.+	Saposin-like	49
Garvicin ML^*d*^	*Lactococcus garvieae*	C	60	+5	br. Gr.+	Saposin-like	46
Carnocyclin A^*e*^	*Carnobacterium maltaromaticum*	C	60	+4	br. Gr.+ *nw. Gr.–	Saposin-like	43
Amylocyclicin^*d*^	*Bacillus amyloliquefaciens*	C	60	+3	Gr.+	Saposin-like	47
Aureocyclicin^*d*^	*Streptococcus aureus*	C	60	+3	N/A	Saposin-like	75
Weissellicin Y^*e*^	*Weissella hellenica*	L	42	+4	br. Gr.+	Saposin-like	76
Weissellicin M^*e*^	*Weissella hellenica*	L	43	+4	br. Gr.+	Saposin-like	76
Lacticin Q^*f*^	*Lactococcus lactis*	L	52	+6	br. Gr.+	Saposin-like	66
Aureocin A53^*f*^	*Staphylococcus aureus*	L	51	+8	br. Gr.+	Saposin-like	77
Epidermicin NI01^*e*^	*Stayphylococcus epidermis*	L	51	+8	br. Gr.+	Saposin-like	78
Mutacin BhtB^*d*^	*Staphylococcus ratti*	L	44	+4	br. Gr.+	Saposin-like	79
Enterocin 7A^*g*^	*Enterococcus faecalis*	L	44	+7	br. Gr.+	Saposin-like	37, 80
Enterocin L50A^*e*^	*Enterococcus faecalis*	L	44	+7	br. Gr.+ **nw. Gr.–	Saposin-like	81
Enterocin 7B^*g*^	*Enterococcus faecalis*	L	43	+7	br. Gr.+	Saposin-like	37, 80
Enterocin L50B^*e*^	*Enterococcus faecalis*	L	43	+7	br. Gr.+ **nw. Gr.–	Saposin-like	81
Acidocin B^*h*^	*Lactobacillus acidophilus*	C	59	+1	br. Gr.+	Helical-bundle	82
Gassericin A^*i*^	*Lactobacillus gasseri*	C	59	+1	br. Gr.+	Helical-bundle	50
Butyrivibriocin AR10^*i*^	*Butyrivibrio fibriosolvens*	C	58	–2	br. Gr.+	Helical-bundle	51

^*a*^Structure modelled and can be found in the main text of ref. 39.

^*b*^Structure experimentally determined by NMR and X-ray crystallography and can be found in ref. 36 and 58.

^*c*^Structure experimentally determined by NMR and can be found in ref. 40.

^*d*^Structure modelled and can be found in the supplementary information attached to this review.

^*e*^Structure modelled and can be found in main text of ref. 38.

^*f*^Structure experimentally determined by NMR and can be found in the main text of ref. 38.

^*g*^Structure experimentally determined by NMR and can be found in the main text of ref. 37.

^*h*^Structure experimentally determined by NMR and can be found in the main text of ref. 41.

^*i*^Structure modelled and can be found in main text of ref. 41.

### Leaderless bacteriocins

Leaderless bacteriocins are characterized by their lack of N-terminal leader sequence during biosynthesis. Without the presence of the N-terminal leader sequence, leaderless bacteriocins do not undergo post-translational modifications that are found in many other bacteriocins, and accordingly, they contain an N-terminal formylmethionine.^4^ Typically these peptides display broad-spectrum activity displayed against Gram-positive bacteria, and occasionally they have been found to display some activity against Gram-negative activity if the outer membrane of the bacteria is disrupted. There have been approximately 20 leaderless bacteriocins identified, some of which are listed in Table 1.^37^

### Common structural features

**Fig. 3:**
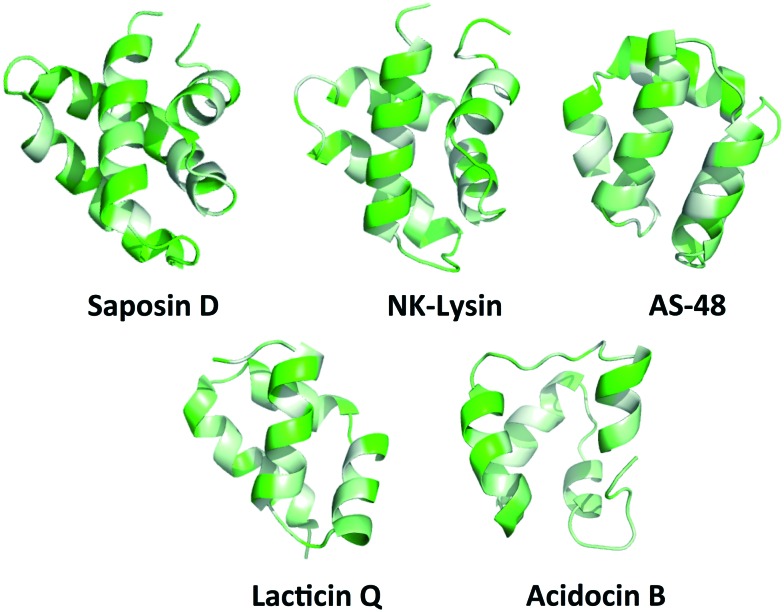
PyMOL ribbon structures of saposin D, NK-lysin, AS-48, lacticin Q, and acidocin B depicting the amphipathic helices that pack in such a way to give a hydrophobic core.^73^ Green indicates hydrophilic residues and white indicates hydrophobic residues. The intensity of the green or white colours indicates the hydrophilicity or hydrophobicity of each amino acid residue, respectively.

While the majority of the circular and leaderless bacteriocins contain this saposin-like fold or the α-helical bundle, depending on the length of the bacteriocin there may be additional α-helices present in these peptides that do not participate in the overall saposin-like fold. In the linear leaderless bacteriocins that contain the saposin like fold, there are typically 3–4 α-helices whereas in the circular bacteriocins there are typically 4–5 α-helices, packed in a saposin-like fold or an α-helical bundle.^37^,^39^,^41^,^53^ It is also worth noting that not all leaderless bacteriocins assume a saposin-like fold. There are some that assume a beta-sheet structure such as laterosporulin and others, such as LsbB, which assume a single α-helix.^18^,^19^ The directionality of the α-helices is quite different between circular and linear bacteriocins that do contain this saposin-like fold, resulting in the ‘v-shape’ being formed by different helices in each case (Fig. 2A/B). The circular bacteriocins that contain four α-helices which associate to create a α-helical bundle have a similar directionality to the circular bacteriocins, which form a true saposin-like fold (Fig. 2C). Some bacteriocins that do not have a saposin-like fold contain structural features (α-helical bundles) that are similar to those that do contain it, and those features will also be discussed here. In all cases, the overall fold of the amphipathic helices results in packing of hydrophobic residues to produce a hydrophobic core (Fig. 3).

### Modes of action

As mentioned above, the circular bacteriocins appear to be active against a broad-spectrum of Gram-positive bacteria, and some even display activity against some Gram-negative bacteria at significantly higher concentrations or when EDTA disrupts the outer membrane.^54^,^55^ Until recently, it has been long thought that the antimicrobial behaviour exhibited by circular bacteriocins was receptor independent due to the broad-spectrum activity observed. Recent studies have suggested that the mode of action of these bacteriocins may, in fact, be much more complicated than originally thought.^42^

**Fig. 4:**
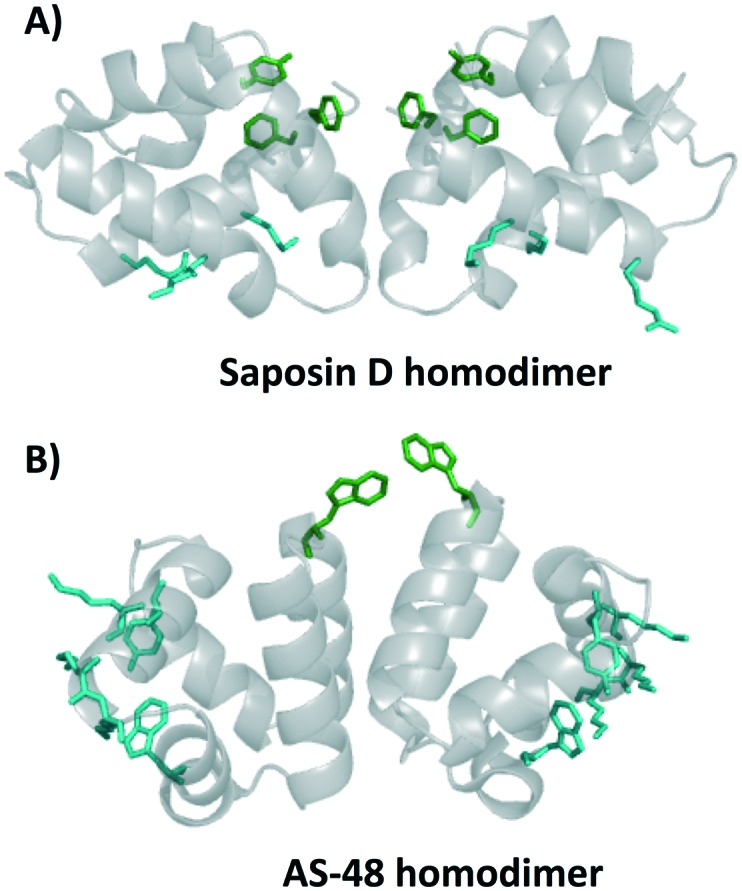
Cartoon representation of homodimer of A) saposin D and B) AS-48. Residues in green indicate surface exposed hydrophobic anchoring amino acids and residues in cyan indicate residues that bind sulphate in the crystal structure.

The first circular bacteriocin to have its structure elucidated was enterocin AS-48, commonly referred to as simply AS-48. AS-48 has garnered much attention due to its broad-spectrum activity against Gram-positive bacteria including *Listeria monocytogenes*, *Clostiridum tyrobutyricum*, *Enterococcus faecalis*, and some strains of *Staphylococcus aureus*. Early kinetic models of bacterial inhibition by AS-48 suggested that multiple molecules of AS-48 were needed to inactivate one bacterial cell.^56^ X-ray crystallography of AS-48 showed that AS-48 forms dimers in solution, though the formation of these dimers appears to be pH dependent.^57^ At a pH of 3 or lower AS-48 is proposed to be monomeric. This is in large part thought to be due to the protonation of glutamic acid residues, which lends additional stability to the monomeric form. Between pH 4.5 and pH 8.5, AS-48 is a dimer, suggesting that under physiological conditions AS-48 exists primarily as a dimer.^57^,^58^ Further studies on the interaction between these AS-48 dimers and lipid membranes showed a conformational alteration upon binding to membranes, going from a closed to open state.^58^,^59^ Interestingly, the saposins have also been shown to form a dimer upon interaction with lipids, in particular, the homodimer of saposin D, which preferentially binds to anionic lipids, appears to be similar to that found in bacteriocins containing the saposin-like fold (Fig. 4A).^60^ In the homodimer of saposin D, it has been proposed that residues that bind sulfate in the crystal structure may bind the anionic lipid in biological environments; these residues are colored cyan. Residues colored green create a hydrophobic section and are proposed to be important in membrane association and potentially membrane anchoring.^60^ Similar characteristics are found in the homodimer of AS-48 (Fig. 4B). Certain SAPLIPs have also been shown form dimers at physiological pH, and it has been proposed that they also undergo a conformational change from open to closed upon interaction of lipids.^25^,^33^ Interestingly, the mode of action of AS-48 on Gram-negative bacteria appears to be concentration dependent. Studies investigating the mode of action of AS-48 against Gram-negative bacteria indicate that at low concentrations the bacterial cells showed a negligible decrease in surviving fractions. A proportional decrease in the percentage of viable bacterial cells was observed upon treatment with increasing amounts of AS-48. Finally, at high concentrations there was very little cell viability.^54^ Some studies suggest that at very low concentrations AS-48 acts in a receptor dependent mechanism, and at high concentrations it acts independently of a receptor. However, other work indicates that even at low concentrations AS-48 can cause leakage in artificial membrane vesicles, suggesting an ability to form pores even in low amounts.^42^,^61^

Extensive studies have been done on the effect of AS-48 on Gram-positive bacteria. It has been shown that relatively low concentrations of bacteriocin can have a detrimental effect on the bacteria. It has been proposed that the primary mode of action for AS-48 is through pore formation. Indeed, it has been shown that small molecules such as leucine, uridine, thymidine and acetate failed to accumulate within the cell and that the concentration of potassium ions within the cytoplasm dropped dramatically within 5 minutes of exposure to AS-48.^56^ Based on the rapid effux of radiolabeled rubidium and slightly slower diffusion of radiolabeled dextran, it has been proposed that AS-48 forms ion channels and pores (∼0.7 nm).^56^

Carnocyclin A displays activity against a wide range of Gram-positive bacteria including a number of strains of *Listeria monocytogenes*, various *Enterococcus* sp., *Lactococcus* sp., *Brocothrix* sp. and some strains of *Staphylococcus aureus.*^43^ Interestingly, disruption of the outer membrane of Gram-negative with EDTA sensitizes some Gram- negative bacteria to carnocyclin A, specifically, *Escherichia coli* and *Pseudomonas aeruginosa.*^62^ Carnocyclin A has also been shown to form small pores or temporary channels in bacterial membranes, but these are anion specific, and unlike AS-48, the ion channels are voltage-dependent.^63^ The pH does not appear to play a role in the fundamental ability to form the channels. However, there is a pronounced difference in the conductivity observed. At a more acidic pH there is a larger conductance observed.^63^ Both carnocyclin A and AS-48 display the ability to permeate liposomes and/or lipid bilayers. This has led to the suggestion that the circular bacteriocins act in a receptor-independent mechanism.^42^ However, recent studies have shown that garvicin ML interacts with the maltose ABC transporter on target cells to illicit antimicrobial activity.^64^ Despite its receptor-dependent mode of action, garvicin ML nevertheless displays a broad spectrum of activity against Gram-positive bacteria. Specifically, garvicin ML appears to be active against various *Enterococcus* sp., *Lactococcus* sp., *Lactobacillus* sp., *Listeria sp*., *Clostoridium sp*., and *Streptococcus* sp.^46^ The recent studies on enterocin NKR-5-3B also indicate general membrane permeation through insertion in the lipid membrane, but the authors suggest that its activity may be due to a combination of electrostatic and hydrophobic interactions along with recognition of a target docking molecule as in the case for garvicin ML.^40^ Enterocin NKR-5-3B is similar to AS-48 carnocyclin A and garvicin ML in that it displays activity against a broad spectrum of Gram-positive bacteria. Specifically, NKR-5-3B is effective against a variety of *Enterococcus* sp., *Bacillus* sp., *Lactococcus* sp, *Lactobacillus* sp. and *Staphylococcus epidermidis.*^40^

Much less work has been done on the mode of action of bacteriocins in subgroup ii. The most well studied circular bacteriocin of subgroup ii is gassericin A, which has a relatively broad spectrum of activity against a variety of food born pathogens such as *Listeria monocytogenes*, *Bacillus cereus*, and *S. aureus.*^65^ It has been suggested that gassericin A causes bacterial cell death through the permeation of the cell membrane and specifically through the efflux of potassium ions.^65^

Similar mode of action and spectrum of activity studies have been performed with leaderless bacteriocins. Lacticin Q is broad spectrum active against a number of Gram-positive bacteria including a number of *Lactobacillus* sp., *Lactococcus* sp., *Enterococcus* sp., *Bacillus* sp., *Listeria* sp., and *Staphylococcus aureus.*^66^ The first mode of action studies on lacticin Q suggested the formation of a huge torroidal pore (4.6–6.6 nm in diameter), significantly larger than the pore size predicted for AS-48 (0.7 nm).^67^ Several features of antimicrobial mode of action make lacticin Q unique with respect to other bacteriocins. Lacticin Q is active at nanomolar concentrations, whereas many of the other bacteriocins described in this review are active at micromolar concentrations. The addition of lacticin Q surprisingly does not create morphological changes in vesicles, unlike AS-48, which creates significant alterations in artificial membrane vesicles. Lacticin Q was also found to translocate and cause lipid flip-flop. The ratios of translocated lacticin Q and lipid flip-flip flop are closely related to the pore formation.^67^,^68^

Not all of the bacteriocins with this backbone structure appear to form pores. Aureocin A53 has been suggested to permeate the membrane but not form discrete pores.^69^ Interestingly, it has been found that aureocin A53 interacts more strongly with neutral membranes than negatively charged membranes, which brings into question the role of negatively charged lipids in the initial electrostatic attraction of this peptides to the membrane.^69^ It appears that even without pore formation, aureocin A53 has broad spectrum activity against a number of notable pathogens, specifically, vancomycin resistant *Enterococcus*, *Listeria innocua* and methicillin resistant and methicillin susceptible strains of *Staphylococcus aureus.*^69^

## Surface properties

### Hydrophobic surfaces

**Fig. 5:**
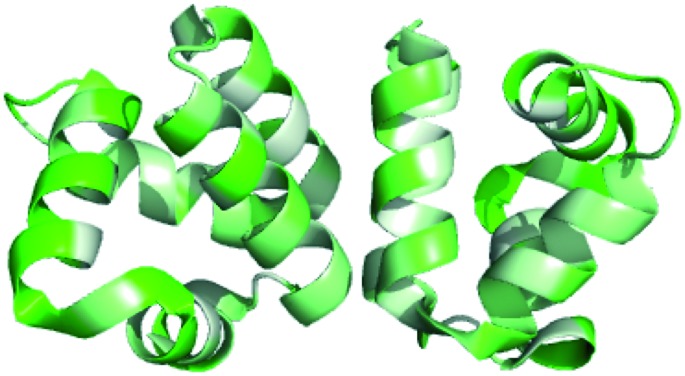
PyMOL cartoon representation of homodimer of AS-48 and the hydrophobic interaction at the dimer face.^73^ Green indicates hydrophilic residues and white indicates hydrophobic residues. The intensity of the green or white colours indicates the hydrophilicity or hydrophobicity of each amino acid residue, respectively.

Although the amphipathic helices pack to form a hydrophobic core, the hydrophobic surface maps reveal large patches of hydrophobicity in the aforementioned bacteriocins. Not surprisingly, in the dimeric structure of AS-48, the patch of hydrophobicity on the surface of the monomer becomes the interacting face of the dimer (Fig. 5). It may be that similar interactions are present in the other bacteriocins with this three dimensional structure, however as seen with the SAPLIPs, there can be many other stabilizing features that favour dimer formation.^31^ In addition, it is possible that the small globular structure of the saposin-like fold could undergo a transition to a more open structure upon interaction with the cell membrane, favoured by greater hydrophobic interactions between the lipid membrane and the hydrophobic core of these bacteriocins. Similar structural transitions have been suggested for SAPLIPs.^33^ Furthermore, each bacteriocin has surface exposed aromatic tryptophan or tyrosine residues, which is an unusual feature for globular folded peptides.

Recently, a connection between the surface exposed tryptophans and a peptide's ability to insert into cell membranes has been suggested.^70^ Previous work has investigated the role of tryptophan in transmembrane helices and has shown that this residue plays an important role in anchoring the peptide in the membrane that can overcome slight hydrophobic mismatches in length between peptide and lipid.^70^ It was later shown that tyrosine, and to some extent, phenylalanine have similar anchoring effects in the peptide–lipid interaction when located at the N-terminus.^71^,^72^ It has been proposed that the anchoring effect is related to the propensity of the indole ring in tryptophan or the hydroxylphenyl ring in tyrosine to be located near the carboxyl group of the lipid, facilitating the peptide–lipid interaction.^72^ Although this model emphasizes how single α-helical transmembrane peptides may anchor and insert into lipid bilayers, it is possible that tyrosine and tryptophan play similar roles in the globular peptide–lipid interactions proposed for circular and leaderless bacteriocins. Upon examination of the locations of tyrosine and tryptophan residues, it is clear that all the circular bacteriocins contain a solvent exposed tryptophan or tyrosine near the N and C-terminal. Similarly, each of the leaderless bacteriocins also appears to have a solvent exposed tyrosine or tryptophan near the N-terminus. It seems likely that these solvent exposed residues play a critical role in initiating membrane permeation by such bacteriocins.

**Fig. 6:**
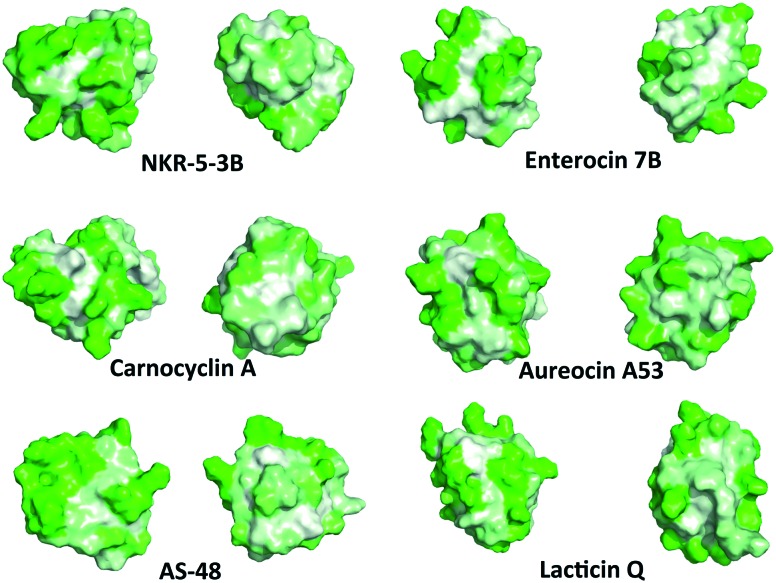
Representative hydrophobic surface structures of various circular and leaderless bacteriocins created with PyMOL.^73^ Green indicates hydrophilic residues and white indicates hydrophobic residues. The intensity of the green or white colours indicates the hydrophilicity or hydrophobicity of each amino acid residue, respectively. Each bacteriocin has one face that has a hydrophobic patch sandwiched between two hydrophilic patches and one predominantly hydrophobic face.

There are several distinct differences between the bacteriocins that contain a saposin-like fold, and acidocin B, gassericin A and butyrivibriocin AR10, which contain an α-helical bundle. Namely, the α-helical bundle has significantly more surface exposed hydrophobic patches. The amphipathic helices in the bacteriocins with the α-helical bundle are loose, resulting in channels straight through the entire peptide. In contrast the amphipathic helices in the bacteriocins with the saposin-like fold are tighter, with no visible channels through the peptide. All of these bacteriocins, regardless of the saposin-like fold or the α-helical bundle, have an interesting commonality, as mentioned earlier there is one primarily hydrophobic side of the peptide. On the opposite side of the peptides there is a hydrophobic patch sandwiched between two hydrophilic patches (Fig. 6). Although its function is uncertain, the patch may bind lipid or a hydrophobic receptor.

## Electrostatic surface of bacteriocins

### Electrostatic potential surface

**Fig. 7:**
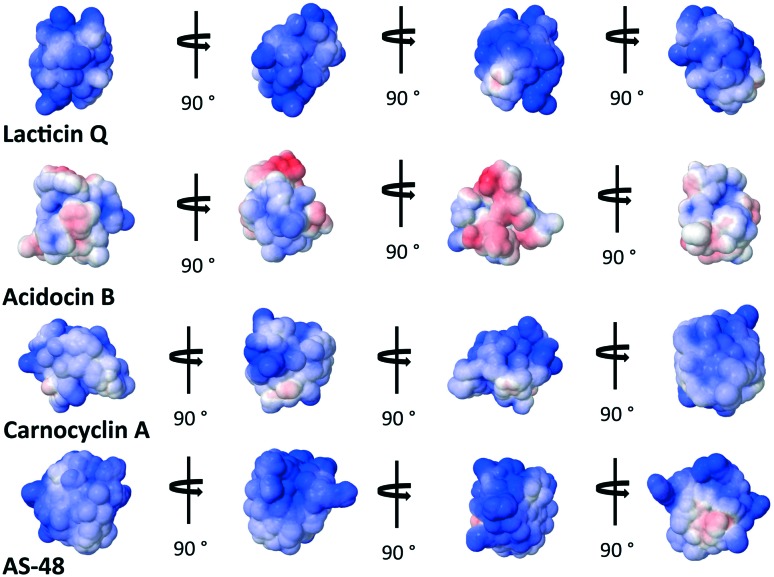
Electrostatic potential maps of select bacteriocins representative of bacteriocins with the saposin-like fold and the α-helical bundle. Cationic regions are blue and anionic regions are red.^83^

The overall cationic charge of many of these bacteriocins has long been thought to play an important role in attracting the bacteriocins to the generally anionic cell membrane.^35^ It is interesting to note that lacticin Q, active at nanomolar concentrations, is actually less cationic at pH = 7 than aureocin A53, active at micromolar concentrations.^69^ This suggests that the mode of action is reliant on more than just the initial electrostatic attraction of the bacteriocin to the bacterial cell membrane. It is curious to note that the bacteriocins that assume a overall α-helical bundle have significantly less overall cationic charge on the surface than those with the saposin-like fold. In fact, acidocin B appears to have a strip of cationic surface charge through the middle of the peptide, capped on either side by anionic surface charge (Fig. 7).

**Fig. 8:**
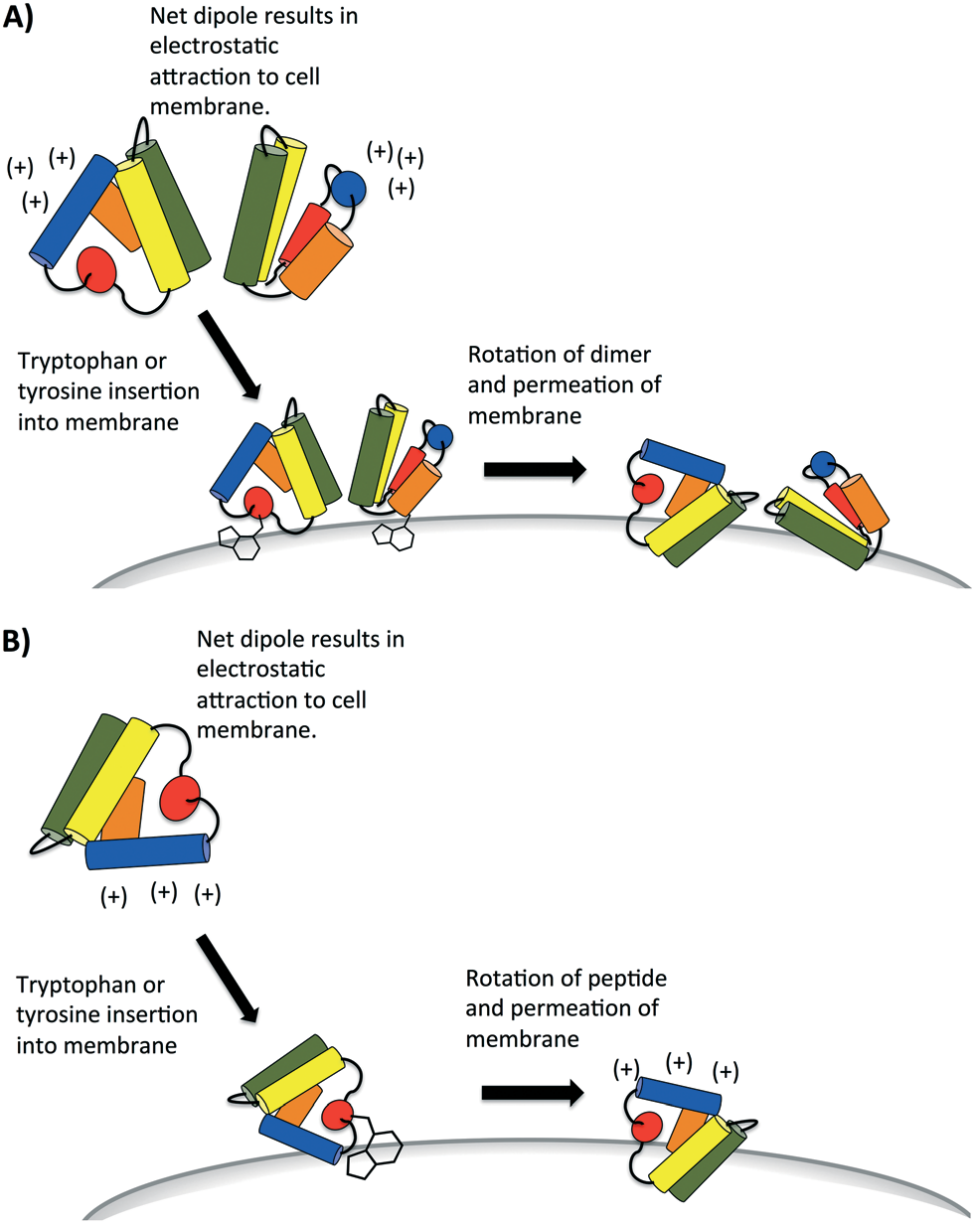
Proposed mechanism of attraction of bacteriocins that contain the saposin-like fold. Some of these bacteriocins (*e.g.* AS-48) are known to form dimers (A) but others remain monomers (B), as they initially interact with the cell membrane of bacteria. They may aggregate later to form pores. Diagrams are recreated from diagrams which have been proposed for some SAPLIP peptides as seen in reference.^25^

A recent study has proposed the initial attraction of the AS-48 dimer occurs through electrostatics; specifically the large dipole moment created in the dimer attracts the dimer to the cell wall.^59^ Upon approach of the dimer, the same study suggests that hydrophobic interactions dominate in the interaction between the peptide and the cell wall. It may be the insertion and anchoring of the surface exposed tryptophan and tyrosines dominate this hydrophobic interaction, which has also been proposed in certain SAPLIPs models.^25^ Finally, accumulation of these peptides compromises the membrane, either by forming pores through aggregation or by general permeation and destabilization (Fig. 8A). It is presently uncertain whether or not all bacteriocins that have a saposin like fold can form dimers. Indeed there are some SAPLIPs which remain as monomers though their proposed mechanism of membrane permeation remains the same (Fig. 8B).^25^ The bacteriocins which form an α-helical bundle and whose overall surface charge is low may rely on hydrophobic interactions if dimers or aggregates are indeed formed in the membrane pore.

## Summary and outlook

Both NMR analyses and molecular modeling show that most circular and many leaderless bacteriocins have a three-dimensional structure that is a saposin-like fold or a closely related α-helical bundle. The helices (typically 4 or 5) all pack to form a hydrophobic core. This then generates an outside surface that has one predominantly hydrophobic face, with the opposite side having two hydrophilic patches that sandwich a hydrophobic channel. All of these peptides have solvent-exposed tryptophan or tyrosine residues near the N- or C-termini. Despite the fact that these bacteriocins display considerable variation in sequence, size and charge, this common motif is repeated in a large number of these compounds. Such antimicrobial peptides are all believed to disrupt the membrane of target bacteria and create holes or pores that leak its cellular contents and cause death of the organism. The requirement for a potential membrane-bound receptor molecule is presently uncertain for most of these bacteriocins, and may not be at all necessary for some. However, it is likely that the initial interaction with the membrane and its subsequent disruption depends on the key structural features, especially the aromatic side chains of the terminal residues and the hydrophobic regions on the surfaces. Future studies may reveal not only possible receptor–bacteriocin interactions in the membrane, but also details of peptide binding to the lipid bilayer. Ultimately, the stoichiometry of pore formation and the physical characteristics of the channels that may be created by clustering of bacteriocins or bacteriocin–receptor complexes should provide valuable insight into the detailed mechanisms of their activity. This will help address the global need for the development of new antibiotics that do not readily develop resistance and are active against a wide variety of pathogens.

## Supplementary Material

Supplementary information
