# Supplementary material for: Structural features of many circular and leaderless bacteriocins are similar to those in saposins and saposin-like peptides
Source: Medchemcomm. 2017 Jan 11;8(2):276–85. doi: 10.1039/c6md00607h (PMC6072434; doi:10.1039/c6md00607h)
Supplement: Supplementary file 1 [file MD-008-C6MD00607H-s001.pdf]

Electronic Supplementary Material (ESI) for MedChemComm.
This journal is © The Royal Society of Chemistry 2017

Supporting information

SWISS-MODEL is an automated structure-homology modeling server that is available through the ExPASy web server.[84] The sequence for each bacteriocin was uploaded to the server followed by an automated search through the Research Collaboratory Structural Bioinformatics' (RCSB) Protein Data Bank (PDB) for a homologous peptide with an experimentally determined structure.[85] PDB coordinates of the peptide with the highest sequence-homology were used to create a structural model of each bacteriocin of interest.

1. Pneumocyclicin

A) PDB file for model of pneumocyclicin. Structure modeled after NKR-5-3B using SWISS-MODEL[84]

```
TITLE     SWISS-MODEL SERVER (https://swissmodel.expasy.org)
TITLE    2 Untitled Project
EXPDTA    THEORETICAL MODEL (SWISS-MODEL SERVER)
AUTHOR    SWISS-MODEL SERVER (SEE REFERENCE IN JRNL
Records)
REVDAT   1   31-OCT-16 1MOD    1       15:24
JRNL        AUTH
M.BIASINI,S.BIENERT,A.WATERHOUSE,K.ARNOLD,G.STUDER,
JRNL        AUTH 2 T.SCHMIDT,F.KIEFER,T.GALLO
CASSARINO,M.BERTONI,L.BORDOLI,
JRNL        AUTH 3 T.SCHWEDE
JRNL        TITL   SWISS-MODEL: MODELLING PROTEIN TERTIARY
AND QUATERNARY
JRNL        TITL 2 STRUCTURE USING EVOLUTIONARY
INFORMATION
JRNL        REF    NUCLEIC.ACIDS.RES                       2014
JRNL        REFN                   ISSN 0305-1048
JRNL        PMID   24782522
JRNL        DOI    10.1093/nar/gku340
REMARK   3 MODEL INFORMATION
REMARK   3  ENGIN   PROMOD3
REMARK   3  VERSN   1.0.1
REMARK   3  OSTAT   MONOMER
REMARK   3  OSRSN   USER
REMARK   3  GMQE    0.79
REMARK   3  QMN4    -1.18
REMARK   3  MODT    FALSE
REMARK   3
REMARK   3 TEMPLATE 1
REMARK   3  PDBID   2mp8
REMARK   3  CHAIN   A
REMARK   3  MMCIF   A
REMARK   3  PDBV    2016-10-14
REMARK   3  SMTLE   2mp8.1.A
REMARK   3  SMTLV   2016-10-19
REMARK   3  MTHD    SOLUTION NMR 0.00 A
REMARK   3  FOUND   HHblits
REMARK   3  GMQE    0.79
REMARK   3  SIM     0.40
REMARK   3  SID     43.75
REMARK   3  OSTAT   monomer
ATOM      1  N   LEU A   1       5.377  -3.179   1.001  1.00  0.75           N
ATOM      2  CA  LEU A   1       4.201  -4.013   0.852  1.00  0.75           C
ATOM      3  C   LEU A   1       4.594  -5.415   0.498  1.00  0.75           C
ATOM      4  O   LEU A   1       4.214  -6.343   1.171  1.00  0.75           O
ATOM      5  CB  LEU A   1       3.366  -3.247  -0.183  1.00  0.75           C
ATOM      6  CG  LEU A   1       2.923  -1.834   0.250  1.00  0.75           C
ATOM      7  CD1 LEU A   1       2.299  -1.181  -0.987  1.00  0.75           C
ATOM      8  CD2 LEU A   1       1.992  -1.884   1.476  1.00  0.75           C
ATOM      9  N   ALA A   2       5.395  -5.633  -0.563  1.00  0.74           N
ATOM     10  CA  ALA A   2       5.744  -6.972  -0.972  1.00  0.74           C
ATOM     11  C   ALA A   2       6.430  -7.903   0.044  1.00  0.74           C
ATOM     12  O   ALA A   2       6.047  -9.051   0.194  1.00  0.74           O
ATOM     13  CB  ALA A   2       6.627  -6.844  -2.216  1.00  0.74           C
ATOM     14  N   GLY A   3       7.421  -7.382   0.793  1.00  0.70           N
ATOM     15  CA  GLY A   3       8.158  -8.029   1.864  1.00  0.70           C
ATOM     16  C   GLY A   3       7.369  -8.184   3.141  1.00  0.70           C
ATOM     17  O   GLY A   3       7.422  -9.245   3.754  1.00  0.70           O
ATOM     18  N   THR A   4       6.617  -7.154   3.605  1.00  0.68           N
ATOM     19  CA  THR A   4       5.835  -7.268   4.852  1.00  0.68           C
ATOM     20  C   THR A   4       4.537  -8.022   4.715  1.00  0.68           C
ATOM     21  O   THR A   4       4.109  -8.717   5.636  1.00  0.68           O
ATOM     22  CB  THR A   4       5.498  -5.972   5.580  1.00  0.68           C
ATOM     23  OG1 THR A   4       4.723  -5.061   4.795  1.00  0.68           O
ATOM     24  CG2 THR A   4       6.810  -5.286   5.969  1.00  0.68           C
ATOM     25  N   LEU A   5       3.857  -7.886   3.565  1.00  0.69           N
ATOM     26  CA  LEU A   5       2.564  -8.491   3.335  1.00  0.69           C
ATOM     27  C   LEU A   5       2.699  -9.831   2.644  1.00  0.69           C
ATOM     28  O   LEU A   5       1.785 -10.649   2.684  1.00  0.69           O
ATOM     29  CB  LEU A   5       1.717  -7.595   2.398  1.00  0.69           C
ATOM     30  CG  LEU A   5       1.561  -6.136   2.860  1.00  0.69           C
ATOM     31  CD1 LEU A   5       0.936  -5.323   1.725  1.00  0.69           C
ATOM     32  CD2 LEU A   5       0.732  -5.999   4.137  1.00  0.69           C
ATOM     33  N   GLY A   6       3.851 -10.082   1.982  1.00  0.76           N
ATOM     34  CA  GLY A   6       4.101 -11.315   1.245  1.00  0.76           C
ATOM     35  C   GLY A   6       3.325 -11.381  -0.040  1.00  0.76           C
ATOM     36  O   GLY A   6       2.784 -12.419  -0.417  1.00  0.76           O
ATOM     37  N   ILE A   7       3.238 -10.241  -0.743  1.00  0.72           N
ATOM     38  CA  ILE A   7       2.450 -10.082  -1.948  1.00  0.72           C
ATOM     39  C   ILE A   7       3.369  -9.882  -3.126  1.00  0.72           C
ATOM     40  O   ILE A   7       4.571  -9.657  -3.002  1.00  0.72           O
ATOM     41  CB  ILE A   7       1.434  -8.933  -1.898  1.00  0.72           C
ATOM     42  CG1 ILE A   7       2.105  -7.556  -1.667  1.00  0.72           C
ATOM     43  CG2 ILE A   7       0.377  -9.288  -0.829  1.00  0.72           C
ATOM     44  CD1 ILE A   7       1.134  -6.374  -1.743  1.00  0.72           C
ATOM     45  N   SER A   8       2.811  -9.971  -4.343  1.00  0.81           N
ATOM     46  CA  SER A   8       3.535  -9.703  -5.569  1.00  0.81           C
ATOM     47  C   SER A   8       3.850  -8.230  -5.753  1.00  0.81           C
ATOM     48  O   SER A   8       3.212  -7.349  -5.178  1.00  0.81           O
ATOM     49  CB  SER A   8       2.779 -10.222  -6.822  1.00  0.81           C
ATOM     50  OG  SER A   8       1.522  -9.560  -7.025  1.00  0.81           O
ATOM     51  N   THR A   9       4.847  -7.908  -6.598  1.00  0.76           N
ATOM     52  CA  THR A   9       5.165  -6.537  -6.986  1.00  0.76           C
ATOM     53  C   THR A   9       4.026  -5.881  -7.745  1.00  0.76           C
ATOM     54  O   THR A   9       3.783  -4.683  -7.608  1.00  0.76           O
ATOM     55  CB  THR A   9       6.427  -6.438  -7.829  1.00  0.76           C
ATOM     56  OG1 THR A   9       6.356  -7.284  -8.969  1.00  0.76           O
ATOM     57  CG2 THR A   9       7.624  -6.929  -7.009  1.00  0.76           C
ATOM     58  N   LYS A  10       3.278  -6.679  -8.541  1.00  0.75           N
ATOM     59  CA  LYS A  10       2.065  -6.280  -9.231  1.00  0.75           C
ATOM     60  C   LYS A  10       0.979  -5.816  -8.284  1.00  0.75           C
ATOM     61  O   LYS A  10       0.424  -4.733  -8.437  1.00  0.75           O
ATOM     62  CB  LYS A  10       1.475  -7.489 -10.008  1.00  0.75           C
ATOM     63  CG  LYS A  10       0.201  -7.170 -10.816  1.00  0.75           C
ATOM     64  CD  LYS A  10      -0.345  -8.403 -11.560  1.00  0.75           C
ATOM     65  CE  LYS A  10      -1.617  -8.103 -12.365  1.00  0.75           C
ATOM     66  NZ  LYS A  10      -2.091  -9.315 -13.077  1.00  0.75           N
```

ATOM 67 N ALA A 11 0.683 -6.620 -7.239 1.00 0.83 N
ATOM 68 CA ALA A 11 -0.249 -6.250 -6.206 1.00 0.83 C
ATOM 69 C ALA A 11 0.267 -5.066 -5.418 1.00 0.83 C
ATOM 70 O ALA A 11 -0.469 -4.118 -5.190 1.00 0.83 O
ATOM 71 CB ALA A 11 -0.520 -7.441 -5.268 1.00 0.83 C
ATOM 72 N ALA A 12 1.563 -5.045 -5.042 1.00 0.82 N
ATOM 73 CA ALA A 12 2.157 -3.940 -4.322 1.00 0.82 C
ATOM 74 C ALA A 12 2.048 -2.602 -5.045 1.00 0.82 C
ATOM 75 O ALA A 12 1.603 -1.629 -4.450 1.00 0.82 O
ATOM 76 CB ALA A 12 3.640 -4.241 -4.032 1.00 0.82 C
ATOM 77 N ALA A 13 2.365 -2.534 -6.354 1.00 0.83 N
ATOM 78 CA ALA A 13 2.195 -1.357 -7.194 1.00 0.83 C
ATOM 79 C ALA A 13 0.747 -0.912 -7.314 1.00 0.83 C
ATOM 80 O ALA A 13 0.409 0.278 -7.328 1.00 0.83 O
ATOM 81 CB ALA A 13 2.736 -1.660 -8.603 1.00 0.83 C
ATOM 82 N THR A 14 -0.174 -1.871 -7.377 1.00 0.82 N
ATOM 83 CA THR A 14 -1.591 -1.613 -7.284 1.00 0.82 C
ATOM 84 C THR A 14 -2.015 -1.032 -5.938 1.00 0.82 C
ATOM 85 O THR A 14 -2.759 -0.056 -5.895 1.00 0.82 O
ATOM 86 CB THR A 14 -2.375 -2.856 -7.592 1.00 0.82 C
ATOM 87 OG1 THR A 14 -2.158 -3.285 -8.924 1.00 0.82 O
ATOM 88 CG2 THR A 14 -3.849 -2.546 -7.539 1.00 0.82 C
ATOM 89 N VAL A 15 -1.507 -1.562 -4.797 1.00 0.82 N
ATOM 90 CA VAL A 15 -1.705 -0.996 -3.457 1.00 0.82 C
ATOM 91 C VAL A 15 -1.162 0.421 -3.402 1.00 0.82 C
ATOM 92 O VAL A 15 -1.830 1.312 -2.878 1.00 0.82 O
ATOM 93 CB VAL A 15 -1.014 -1.776 -2.331 1.00 0.82 C
ATOM 94 CG1 VAL A 15 -1.220 -1.107 -0.947 1.00 0.82 C
ATOM 95 CG2 VAL A 15 -1.489 -3.234 -2.232 1.00 0.82 C
ATOM 96 N VAL A 16 0.029 0.670 -3.998 1.00 0.81 N
ATOM 97 CA VAL A 16 0.653 1.983 -4.145 1.00 0.81 C
ATOM 98 C VAL A 16 -0.275 2.981 -4.808 1.00 0.81 C
ATOM 99 O VAL A 16 -0.485 4.082 -4.301 1.00 0.81 O
ATOM 100 CB VAL A 16 1.950 1.935 -4.953 1.00 0.81 C
ATOM 101 CG1 VAL A 16 2.437 3.331 -5.386 1.00 0.81 C
ATOM 102 CG2 VAL A 16 3.065 1.233 -4.159 1.00 0.81 C
ATOM 103 N ASN A 17 -0.914 2.626 -5.934 1.00 0.79 N
ATOM 104 CA ASN A 17 -1.861 3.512 -6.590 1.00 0.79 C
ATOM 105 C ASN A 17 -3.042 3.877 -5.713 1.00 0.79 C
ATOM 106 O ASN A 17 -3.482 5.023 -5.674 1.00 0.79 O
ATOM 107 CB ASN A 17 -2.429 2.875 -7.872 1.00 0.79 C
ATOM 108 CG ASN A 17 -1.359 2.837 -8.948 1.00 0.79 C
ATOM 109 OD1 ASN A 17 -0.439 3.655 -8.994 1.00 0.79
O
ATOM 110 ND2 ASN A 17 -1.511 1.884 -9.894 1.00 0.79
N
ATOM 111 N LEU A 18 -3.578 2.899 -4.973 1.00 0.77 N
ATOM 112 CA LEU A 18 -4.656 3.130 -4.047 1.00 0.77 C
ATOM 113 C LEU A 18 -4.326 3.958 -2.838 1.00 0.77 C
ATOM 114 O LEU A 18 -5.012 4.924 -2.531 1.00 0.77 O
ATOM 115 CB LEU A 18 -5.147 1.785 -3.535 1.00 0.77 C
ATOM 116 CG LEU A 18 -5.679 0.876 -4.631 1.00 0.77 C
ATOM 117 CD1 LEU A 18 -6.041 -0.415 -3.933 1.00 0.77 C
ATOM 118 CD2 LEU A 18 -6.911 1.495 -5.276 1.00 0.77 C
ATOM 119 N ILE A 19 -3.238 3.681 -2.122 1.00 0.74 N
ATOM 120 CA ILE A 19 -2.875 4.478 -0.970 1.00 0.74 C
ATOM 121 C ILE A 19 -2.499 5.906 -1.350 1.00 0.74 C
ATOM 122 O ILE A 19 -2.643 6.822 -0.555 1.00 0.74 O
ATOM 123 CB ILE A 19 -1.735 3.832 -0.222 1.00 0.74 C
ATOM 124 CG1 ILE A 19 -0.507 3.763 -1.149 1.00 0.74 C
ATOM 125 CG2 ILE A 19 -2.204 2.438 0.265 1.00 0.74 C
ATOM 126 CD1 ILE A 19 0.722 3.194 -0.495 1.00 0.74 C
ATOM 127 N SER A 20 -1.979 6.094 -2.587 1.00 0.78 N
ATOM 128 CA SER A 20 -1.607 7.377 -3.157 1.00 0.78 C
ATOM 129 C SER A 20 -2.798 8.206 -3.571 1.00 0.78 C
ATOM 130 O SER A 20 -2.793 9.425 -3.421 1.00 0.78 O
ATOM 131 CB SER A 20 -0.737 7.242 -4.434 1.00 0.78 C
ATOM 132 OG SER A 20 0.600 6.819 -4.148 1.00 0.78 O
ATOM 133 N ALA A 21 -3.827 7.575 -4.175 1.00 0.74 N
ATOM 134 CA ALA A 21 -4.995 8.260 -4.692 1.00 0.74 C
ATOM 135 C ALA A 21 -6.026 8.600 -3.641 1.00 0.74 C
ATOM 136 O ALA A 21 -6.664 9.649 -3.666 1.00 0.74 O
ATOM 137 CB ALA A 21 -5.690 7.370 -5.742 1.00 0.74 C
ATOM 138 N TYR A 22 -6.242 7.664 -2.711 1.00 0.69 N
ATOM 139 CA TYR A 22 -7.238 7.764 -1.686 1.00 0.69 C
ATOM 140 C TYR A 22 -6.706 8.543 -0.515 1.00 0.69 C
ATOM 141 O TYR A 22 -5.517 8.547 -0.226 1.00 0.69 O
ATOM 142 CB TYR A 22 -7.576 6.371 -1.133 1.00 0.69 C
ATOM 143 CG TYR A 22 -8.435 5.564 -2.034 1.00 0.69 C
ATOM 144 CD1 TYR A 22 -7.989 5.135 -3.287 1.00 0.69 C
ATOM 145 CD2 TYR A 22 -9.715 5.190 -1.619 1.00 0.69 C
ATOM 146 CE1 TYR A 22 -8.858 4.516 -4.179 1.00 0.69 C
ATOM 147 CE2 TYR A 22 -10.598 4.563 -2.507 1.00 0.69
C
ATOM 148 CZ TYR A 22 -10.178 4.290 -3.813 1.00 0.69 C
ATOM 149 OH TYR A 22 -11.011 3.718 -4.783 1.00 0.69
O
ATOM 150 N SER A 23 -7.623 9.195 0.213 1.00 0.66 N
ATOM 151 CA SER A 23 -7.266 10.130 1.263 1.00 0.66 C
ATOM 152 C SER A 23 -7.506 9.530 2.624 1.00 0.66 C
ATOM 153 O SER A 23 -6.588 9.223 3.388 1.00 0.66 O
ATOM 154 CB SER A 23 -8.132 11.409 1.143 1.00 0.66 C
ATOM 155 OG SER A 23 -7.847 12.060 -0.092 1.00 0.66 O
ATOM 156 N THR A 24 -8.786 9.355 2.990 1.00 0.68 N
ATOM 157 CA THR A 24 -9.224 8.781 4.253 1.00 0.68 C
ATOM 158 C THR A 24 -8.760 7.358 4.431 1.00 0.68 C
ATOM 159 O THR A 24 -8.978 6.525 3.558 1.00 0.68 O
ATOM 160 CB THR A 24 -10.741 8.794 4.359 1.00 0.68 C
ATOM 161 OG1 THR A 24 -11.182 10.140 4.304 1.00 0.68
O
ATOM 162 CG2 THR A 24 -11.265 8.198 5.674 1.00 0.68
C
ATOM 163 N VAL A 25 -8.165 7.015 5.597 1.00 0.71 N
ATOM 164 CA VAL A 25 -7.587 5.701 5.847 1.00 0.71 C
ATOM 165 C VAL A 25 -8.601 4.582 5.658 1.00 0.71 C
ATOM 166 O VAL A 25 -8.359 3.625 4.936 1.00 0.71 O
ATOM 167 CB VAL A 25 -7.017 5.624 7.260 1.00 0.71 C
ATOM 168 CG1 VAL A 25 -6.364 4.249 7.516 1.00 0.71 C
ATOM 169 CG2 VAL A 25 -5.966 6.735 7.448 1.00 0.71 C
ATOM 170 N THR A 26 -9.813 4.725 6.220 1.00 0.70 N
ATOM 171 CA THR A 26 -10.935 3.794 6.066 1.00 0.70 C
ATOM 172 C THR A 26 -11.333 3.558 4.630 1.00 0.70 C
ATOM 173 O THR A 26 -11.600 2.427 4.214 1.00 0.70 O
ATOM 174 CB THR A 26 -12.149 4.289 6.835 1.00 0.70 C
ATOM 175 OG1 THR A 26 -11.798 4.409 8.205 1.00 0.70
O
ATOM 176 CG2 THR A 26 -13.340 3.323 6.743 1.00 0.70
C
ATOM 177 N ALA A 27 -11.342 4.621 3.808 1.00 0.74 N
ATOM 178 CA ALA A 27 -11.603 4.546 2.389 1.00 0.74 C
ATOM 179 C ALA A 27 -10.510 3.767 1.660 1.00 0.74 C
ATOM 180 O ALA A 27 -10.801 2.930 0.803 1.00 0.74 O
ATOM 181 CB ALA A 27 -11.723 5.971 1.815 1.00 0.74 C
ATOM 182 N VAL A 28 -9.226 4.009 2.033 1.00 0.75 N
ATOM 183 CA VAL A 28 -8.062 3.249 1.578 1.00 0.75 C
ATOM 184 C VAL A 28 -8.154 1.784 1.906 1.00 0.75 C
ATOM 185 O VAL A 28 -8.015 0.928 1.047 1.00 0.75 O
ATOM 186 CB VAL A 28 -6.723 3.712 2.138 1.00 0.75 C
ATOM 187 CG1 VAL A 28 -5.575 2.947 1.470 1.00 0.75 C
ATOM 188 CG2 VAL A 28 -6.475 5.149 1.728 1.00 0.75 C
ATOM 189 N ILE A 29 -8.454 1.421 3.154 1.00 0.70 N
ATOM 190 CA ILE A 29 -8.473 0.026 3.559 1.00 0.70 C
ATOM 191 C ILE A 29 -9.472 -0.801 2.767 1.00 0.70 C
ATOM 192 O ILE A 29 -9.167 -1.893 2.276 1.00 0.70 O
ATOM 193 CB ILE A 29 -8.832 -0.062 5.025 1.00 0.70 C
ATOM 194 CG1 ILE A 29 -7.771 0.637 5.895 1.00 0.70 C
ATOM 195 CG2 ILE A 29 -8.978 -1.536 5.461 1.00 0.70 C
ATOM 196 CD1 ILE A 29 -8.316 0.898 7.297 1.00 0.70 C

ATOM 197 N SER A 30 -10.686 -0.250 2.580 1.00 0.77 N
ATOM 198 CA SER A 30 -11.761 -0.880 1.832 1.00 0.77 C
ATOM 199 C SER A 30 -11.417 -1.109 0.391 1.00 0.77 C
ATOM 200 O SER A 30 -11.705 -2.169 -0.170 1.00 0.77 O
ATOM 201 CB SER A 30 -13.046 -0.030 1.849 1.00 0.77 C
ATOM 202 OG SER A 30 -13.559 0.025 3.179 1.00 0.77 O
ATOM 203 N ILE A 31 -10.777 -0.125 -0.263 1.00 0.73 N
ATOM 204 CA ILE A 31 -10.334 -0.311 -1.625 1.00 0.73 C
ATOM 205 C ILE A 31 -9.185 -1.302 -1.761 1.00 0.73 C
ATOM 206 O ILE A 31 -9.217 -2.178 -2.621 1.00 0.73 O
ATOM 207 CB ILE A 31 -10.034 0.994 -2.337 1.00 0.73 C
ATOM 208 CG1 ILE A 31 -9.939 0.766 -3.857 1.00 0.73 C
ATOM 209 CG2 ILE A 31 -8.790 1.691 -1.751 1.00 0.73 C
ATOM 210 CD1 ILE A 31 -11.276 0.537 -4.562 1.00 0.73 C
ATOM 211 N VAL A 32 -8.147 -1.240 -0.893 1.00 0.78 N
ATOM 212 CA VAL A 32 -6.986 -2.107 -1.005 1.00 0.78 C
ATOM 213 C VAL A 32 -7.378 -3.544 -0.794 1.00 0.78 C
ATOM 214 O VAL A 32 -6.976 -4.430 -1.554 1.00 0.78 O
ATOM 215 CB VAL A 32 -5.833 -1.742 -0.078 1.00 0.78 C
ATOM 216 CG1 VAL A 32 -4.619 -2.582 -0.483 1.00 0.78
C
ATOM 217 CG2 VAL A 32 -5.381 -0.279 -0.231 1.00 0.78
C
ATOM 218 N GLY A 33 -8.249 -3.792 0.197 1.00 0.76 N
ATOM 219 CA GLY A 33 -8.784 -5.112 0.474 1.00 0.76 C
ATOM 220 C GLY A 33 -9.632 -5.702 -0.626 1.00 0.76 C
ATOM 221 O GLY A 33 -9.616 -6.911 -0.843 1.00 0.76 O
ATOM 222 N ALA A 34 -10.368 -4.853 -1.374 1.00 0.76 N
ATOM 223 CA ALA A 34 -11.126 -5.227 -2.554 1.00 0.76
C
ATOM 224 C ALA A 34 -10.250 -5.649 -3.720 1.00 0.76 C
ATOM 225 O ALA A 34 -10.588 -6.547 -4.488 1.00 0.76 O
ATOM 226 CB ALA A 34 -12.033 -4.057 -2.997 1.00 0.76
C
ATOM 227 N ILE A 35 -9.103 -4.980 -3.889 1.00 0.72 N
ATOM 228 CA ILE A 35 -8.169 -5.275 -4.947 1.00 0.72 C
ATOM 229 C ILE A 35 -7.242 -6.443 -4.658 1.00 0.72 C
ATOM 230 O ILE A 35 -7.026 -7.307 -5.509 1.00 0.72 O
ATOM 231 CB ILE A 35 -7.339 -4.047 -5.188 1.00 0.72 C
ATOM 232 CG1 ILE A 35 -8.143 -2.848 -5.753 1.00 0.72 C
ATOM 233 CG2 ILE A 35 -6.197 -4.350 -6.150 1.00 0.72 C
ATOM 234 CD1 ILE A 35 -9.004 -3.078 -7.000 1.00 0.72 C
ATOM 235 N THR A 36 -6.631 -6.510 -3.458 1.00 0.74 N
ATOM 236 CA THR A 36 -5.709 -7.595 -3.143 1.00 0.74 C
ATOM 237 C THR A 36 -6.442 -8.860 -2.755 1.00 0.74 C
ATOM 238 O THR A 36 -5.874 -9.949 -2.817 1.00 0.74 O
ATOM 239 CB THR A 36 -4.712 -7.275 -2.032 1.00 0.74 C
ATOM 240 OG1 THR A 36 -5.359 -6.851 -0.841 1.00 0.74
O
ATOM 241 CG2 THR A 36 -3.790 -6.127 -2.459 1.00 0.74
C
ATOM 242 N GLY A 37 -7.732 -8.748 -2.361 1.00 0.77 N
ATOM 243 CA GLY A 37 -8.578 -9.876 -1.996 1.00 0.77 C
ATOM 244 C GLY A 37 -8.225 -10.445 -0.650 1.00 0.77 C
ATOM 245 O GLY A 37 -8.328 -11.640 -0.409 1.00 0.77 O
ATOM 246 N VAL A 38 -7.764 -9.577 0.267 1.00 0.66 N
ATOM 247 CA VAL A 38 -7.295 -9.989 1.572 1.00 0.66 C
ATOM 248 C VAL A 38 -7.358 -8.784 2.498 1.00 0.66 C
ATOM 249 O VAL A 38 -7.213 -7.641 2.077 1.00 0.66 O
ATOM 250 CB VAL A 38 -5.882 -10.605 1.507 1.00 0.66 C
ATOM 251 CG1 VAL A 38 -4.841 -9.587 0.995 1.00 0.66 C
ATOM 252 CG2 VAL A 38 -5.462 -11.287 2.827 1.00 0.66
C
ATOM 253 N GLY A 39 -7.543 -9.001 3.819 1.00 0.68 N
ATOM 254 CA GLY A 39 -7.537 -7.952 4.844 1.00 0.68 C
ATOM 255 C GLY A 39 -6.158 -7.700 5.397 1.00 0.68 C
ATOM 256 O GLY A 39 -5.999 -7.288 6.540 1.00 0.68 O
ATOM 257 N SER A 40 -5.122 -7.997 4.588 1.00 0.67 N
ATOM 258 CA SER A 40 -3.705 -7.844 4.914 1.00 0.67 C
ATOM 259 C SER A 40 -3.321 -6.382 5.073 1.00 0.67 C
ATOM 260 O SER A 40 -2.645 -5.977 6.021 1.00 0.67 O
ATOM 261 CB SER A 40 -2.815 -8.507 3.822 1.00 0.67 C
ATOM 262 OG SER A 40 -1.435 -8.518 4.180 1.00 0.67 O
ATOM 263 N ILE A 41 -3.815 -5.512 4.167 1.00 0.71 N
ATOM 264 CA ILE A 41 -3.589 -4.080 4.234 1.00 0.71 C
ATOM 265 C ILE A 41 -4.645 -3.491 5.141 1.00 0.71 C
ATOM 266 O ILE A 41 -5.737 -3.105 4.731 1.00 0.71 O
ATOM 267 CB ILE A 41 -3.554 -3.359 2.886 1.00 0.71 C
ATOM 268 CG1 ILE A 41 -2.420 -3.915 1.984 1.00 0.71 C
ATOM 269 CG2 ILE A 41 -3.340 -1.835 3.117 1.00 0.71 C
ATOM 270 CD1 ILE A 41 -2.741 -5.207 1.212 1.00 0.71 C
ATOM 271 N GLY A 42 -4.319 -3.449 6.443 1.00 0.80 N
ATOM 272 CA GLY A 42 -5.126 -2.805 7.460 1.00 0.80 C
ATOM 273 C GLY A 42 -4.808 -1.343 7.575 1.00 0.80 C
ATOM 274 O GLY A 42 -4.010 -0.783 6.827 1.00 0.80 O
ATOM 275 N SER A 43 -5.388 -0.683 8.593 1.00 0.76 N
ATOM 276 CA SER A 43 -5.141 0.722 8.903 1.00 0.76 C
ATOM 277 C SER A 43 -3.691 1.025 9.207 1.00 0.76 C
ATOM 278 O SER A 43 -3.152 2.046 8.773 1.00 0.76 O
ATOM 279 CB SER A 43 -5.996 1.206 10.107 1.00 0.76 C
ATOM 280 OG SER A 43 -5.731 0.438 11.284 1.00 0.76 O
ATOM 281 N GLY A 44 -3.011 0.112 9.927 1.00 0.80 N
ATOM 282 CA GLY A 44 -1.590 0.204 10.230 1.00 0.80 C
ATOM 283 C GLY A 44 -0.724 0.082 9.013 1.00 0.80 C
ATOM 284 O GLY A 44 0.266 0.794 8.871 1.00 0.80 O
ATOM 285 N ILE A 45 -1.085 -0.791 8.059 1.00 0.73 N
ATOM 286 CA ILE A 45 -0.381 -0.882 6.793 1.00 0.73 C
ATOM 287 C ILE A 45 -0.599 0.363 5.958 1.00 0.73 C
ATOM 288 O ILE A 45 0.378 0.924 5.480 1.00 0.73 O
ATOM 289 CB ILE A 45 -0.735 -2.140 6.013 1.00 0.73 C
ATOM 290 CG1 ILE A 45 -0.477 -3.418 6.854 1.00 0.73 C
ATOM 291 CG2 ILE A 45 0.041 -2.198 4.673 1.00 0.73 C
ATOM 292 CD1 ILE A 45 0.979 -3.616 7.304 1.00 0.73 C
ATOM 293 N ALA A 46 -1.855 0.859 5.832 1.00 0.80 N
ATOM 294 CA ALA A 46 -2.228 2.062 5.101 1.00 0.80 C
ATOM 295 C ALA A 46 -1.597 3.364 5.593 1.00 0.80 C
ATOM 296 O ALA A 46 -1.137 4.182 4.812 1.00 0.80 O
ATOM 297 CB ALA A 46 -3.748 2.270 5.131 1.00 0.80 C
ATOM 298 N ALA A 47 -1.551 3.569 6.927 1.00 0.79 N
ATOM 299 CA ALA A 47 -0.885 4.707 7.518 1.00 0.79 C
ATOM 300 C ALA A 47 0.618 4.648 7.318 1.00 0.79 C
ATOM 301 O ALA A 47 1.243 5.617 6.875 1.00 0.79 O
ATOM 302 CB ALA A 47 -1.215 4.727 9.025 1.00 0.79 C
ATOM 303 N THR A 48 1.244 3.483 7.574 1.00 0.78 N
ATOM 304 CA THR A 48 2.681 3.298 7.401 1.00 0.78 C
ATOM 305 C THR A 48 3.103 3.454 5.967 1.00 0.78 C
ATOM 306 O THR A 48 4.037 4.181 5.655 1.00 0.78 O
ATOM 307 CB THR A 48 3.159 1.938 7.873 1.00 0.78 C
ATOM 308 OG1 THR A 48 2.900 1.798 9.259 1.00 0.78 O
ATOM 309 CG2 THR A 48 4.675 1.764 7.754 1.00 0.78 C
ATOM 310 N VAL A 49 2.384 2.815 5.030 1.00 0.80 N
ATOM 311 CA VAL A 49 2.704 2.860 3.625 1.00 0.80 C
ATOM 312 C VAL A 49 2.613 4.250 3.012 1.00 0.80 C
ATOM 313 O VAL A 49 3.543 4.678 2.336 1.00 0.80 O
ATOM 314 CB VAL A 49 1.899 1.823 2.873 1.00 0.80 C
ATOM 315 CG1 VAL A 49 0.424 2.193 2.756 1.00 0.80 C
ATOM 316 CG2 VAL A 49 2.543 1.524 1.530 1.00 0.80 C
ATOM 317 N LEU A 50 1.558 5.051 3.296 1.00 0.75 N
ATOM 318 CA LEU A 50 1.455 6.406 2.779 1.00 0.75 C
ATOM 319 C LEU A 50 2.543 7.295 3.351 1.00 0.75 C
ATOM 320 O LEU A 50 3.072 8.212 2.714 1.00 0.75 O
ATOM 321 CB LEU A 50 0.057 7.009 3.048 1.00 0.75 C
ATOM 322 CG LEU A 50 -0.175 8.389 2.390 1.00 0.75 C
ATOM 323 CD1 LEU A 50 0.067 8.353 0.871 1.00 0.75 C
ATOM 324 CD2 LEU A 50 -1.595 8.898 2.676 1.00 0.75 C
ATOM 325 N TYR A 51 2.942 7.020 4.600 1.00 0.71 N
ATOM 326 CA TYR A 51 4.029 7.710 5.250 1.00 0.71 C
ATOM 327 C TYR A 51 5.370 7.366 4.664 1.00 0.71 C

ATOM 328 O TYR A 51 6.197 8.262 4.536 1.00 0.71 O
ATOM 329 CB TYR A 51 4.039 7.494 6.775 1.00 0.71 C
ATOM 330 CG TYR A 51 2.860 8.147 7.454 1.00 0.71 C
ATOM 331 CD1 TYR A 51 1.832 8.854 6.788 1.00 0.71 C
ATOM 332 CD2 TYR A 51 2.766 7.999 8.845 1.00 0.71 C
ATOM 333 CE1 TYR A 51 0.736 9.357 7.492 1.00 0.71 C
ATOM 334 CE2 TYR A 51 1.681 8.532 9.555 1.00 0.71 C
ATOM 335 CZ TYR A 51 0.666 9.208 8.871 1.00 0.71 C
ATOM 336 OH TYR A 51 -0.444 9.747 9.543 1.00 0.71 O
ATOM 337 N ILE A 52 5.612 6.091 4.291 1.00 0.72 N
ATOM 338 CA ILE A 52 6.763 5.620 3.525 1.00 0.72 C
ATOM 339 C ILE A 52 6.829 6.290 2.165 1.00 0.72 C
ATOM 340 O ILE A 52 7.891 6.753 1.760 1.00 0.72 O
ATOM 341 CB ILE A 52 6.773 4.099 3.369 1.00 0.72 C
ATOM 342 CG1 ILE A 52 6.997 3.418 4.736 1.00 0.72 C
ATOM 343 CG2 ILE A 52 7.856 3.634 2.363 1.00 0.72 C
ATOM 344 CD1 ILE A 52 6.730 1.912 4.683 1.00 0.72 C
ATOM 345 N LEU A 53 5.680 6.421 1.463 1.00 0.74 N
ATOM 346 CA LEU A 53 5.570 7.117 0.182 1.00 0.74 C
ATOM 347 C LEU A 53 5.933 8.548 0.224 1.00 0.74 C
ATOM 348 O LEU A 53 6.474 9.108 -0.725 1.00 0.74 O
ATOM 349 CB LEU A 53 4.166 7.049 -0.471 1.00 0.74 C
ATOM 350 CG LEU A 53 4.154 5.825 -1.378 1.00 0.74 C
ATOM 351 CD1 LEU A 53 4.430 4.518 -0.672 1.00 0.74 C
ATOM 352 CD2 LEU A 53 2.961 5.556 -2.279 1.00 0.74 C
ATOM 353 N LYS A 54 5.597 9.180 1.336 1.00 0.72 N
ATOM 354 CA LYS A 54 5.916 10.551 1.548 1.00 0.72 C
ATOM 355 C LYS A 54 7.403 10.833 1.730 1.00 0.72 C
ATOM 356 O LYS A 54 7.921 11.798 1.182 1.00 0.72 O
ATOM 357 CB LYS A 54 5.123 11.017 2.783 1.00 0.72 C
ATOM 358 CG LYS A 54 5.310 12.501 3.118 1.00 0.72 C
ATOM 359 CD LYS A 54 4.863 13.444 1.984 1.00 0.72 C
ATOM 360 CE LYS A 54 3.367 13.407 1.662 1.00 0.72 C
ATOM 361 NZ LYS A 54 2.611 13.931 2.819 1.00 0.72 N
ATOM 362 N LYS A 55 8.115 10.007 2.530 1.00 0.69 N
ATOM 363 CA LYS A 55 9.534 10.218 2.783 1.00 0.69 C
ATOM 364 C LYS A 55 10.447 9.556 1.777 1.00 0.69 C
ATOM 365 O LYS A 55 11.380 10.170 1.270 1.00 0.69 O
ATOM 366 CB LYS A 55 9.968 9.768 4.214 1.00 0.69 C
ATOM 367 CG LYS A 55 9.386 8.432 4.712 1.00 0.69 C
ATOM 368 CD LYS A 55 9.691 8.090 6.183 1.00 0.69 C
ATOM 369 CE LYS A 55 9.109 9.079 7.206 1.00 0.69 C
ATOM 370 NZ LYS A 55 7.627 9.075 7.161 1.00 0.69 N
ATOM 371 N LYS A 56 10.223 8.269 1.485 1.00 0.68 N
ATOM 372 CA LYS A 56 11.139 7.494 0.692 1.00 0.68 C
ATOM 373 C LYS A 56 10.633 7.310 -0.727 1.00 0.68 C
ATOM 374 O LYS A 56 11.413 7.284 -1.675 1.00 0.68 O
ATOM 375 CB LYS A 56 11.278 6.108 1.348 1.00 0.68 C
ATOM 376 CG LYS A 56 11.985 6.125 2.719 1.00 0.68 C
ATOM 377 CD LYS A 56 12.084 4.706 3.309 1.00 0.68 C
ATOM 378 CE LYS A 56 12.877 4.537 4.611 1.00 0.68 C
ATOM 379 NZ LYS A 56 12.877 3.120 5.078 1.00 0.68 N
ATOM 380 N GLY A 57 9.300 7.197 -0.906 1.00 0.79 N
ATOM 381 CA GLY A 57 8.677 7.161 -2.222 1.00 0.79 C
ATOM 382 C GLY A 57 7.874 5.920 -2.520 1.00 0.79 C
ATOM 383 O GLY A 57 7.926 4.903 -1.821 1.00 0.79 O
ATOM 384 N ALA A 58 7.097 5.978 -3.620 1.00 0.80 N
ATOM 385 CA ALA A 58 6.190 4.944 -4.096 1.00 0.80 C
ATOM 386 C ALA A 58 6.826 3.604 -4.378 1.00 0.80 C
ATOM 387 O ALA A 58 6.342 2.550 -3.964 1.00 0.80 O
ATOM 388 CB ALA A 58 5.426 5.513 -5.308 1.00 0.80 C
ATOM 389 N ALA A 59 7.990 3.627 -5.022 1.00 0.77 N
ATOM 390 CA ALA A 59 8.762 2.448 -5.289 1.00 0.77 C
ATOM 391 C ALA A 59 9.342 1.818 -4.030 1.00 0.77 C
ATOM 392 O ALA A 59 9.486 0.594 -3.934 1.00 0.77 O
ATOM 393 CB ALA A 59 9.853 2.866 -6.280 1.00 0.77 C
ATOM 394 N LYS A 60 9.670 2.621 -2.999 1.00 0.71 N
ATOM 395 CA LYS A 60 10.187 2.089 -1.761 1.00 0.71 C
ATOM 396 C LYS A 60 9.185 1.267 -0.994 1.00 0.71 C
ATOM 397 O LYS A 60 9.487 0.199 -0.471 1.00 0.71 O
ATOM 398 CB LYS A 60 10.604 3.198 -0.782 1.00 0.71 C
ATOM 399 CG LYS A 60 11.137 2.640 0.553 1.00 0.71 C
ATOM 400 CD LYS A 60 12.485 1.918 0.383 1.00 0.71 C
ATOM 401 CE LYS A 60 13.134 1.463 1.690 1.00 0.71 C
ATOM 402 NZ LYS A 60 14.401 0.735 1.439 1.00 0.71 N
ATOM 403 N ALA A 61 7.955 1.780 -0.876 1.00 0.80 N
ATOM 404 CA ALA A 61 6.961 1.014 -0.188 1.00 0.80 C
ATOM 405 C ALA A 61 6.417 -0.074 -1.034 1.00 0.80 C
ATOM 406 O ALA A 61 5.898 -1.062 -0.443 1.00 0.80 O
ATOM 407 CB ALA A 61 5.747 1.837 0.097 1.00 0.80 C
ATOM 408 N ALA A 62 6.503 -0.065 -2.351 1.00 0.80 N
ATOM 409 CA ALA A 62 6.152 -1.200 -3.159 1.00 0.80 C
ATOM 410 C ALA A 62 6.983 -2.415 -2.717 1.00 0.80 C
ATOM 411 O ALA A 62 6.445 -3.489 -2.429 1.00 0.80 O
ATOM 412 CB ALA A 62 6.384 -0.873 -4.645 1.00 0.80 C
ATOM 413 N LEU A 63 8.298 -2.191 -2.521 1.00 0.65 N
ATOM 414 CA LEU A 63 9.246 -3.154 -1.982 1.00 0.65 C
ATOM 415 C LEU A 63 9.161 -3.576 -0.513 1.00 0.65 C
ATOM 416 O LEU A 63 9.526 -4.705 -0.225 1.00 0.65 O
ATOM 417 CB LEU A 63 10.702 -2.673 -2.121 1.00 0.65 C
ATOM 418 CG LEU A 63 11.239 -2.545 -3.550 1.00 0.65 C
ATOM 419 CD1 LEU A 63 12.637 -1.915 -3.469 1.00 0.65
C
ATOM 420 CD2 LEU A 63 11.307 -3.913 -4.251 1.00 0.65
C
ATOM 421 N TRP A 64 8.758 -2.647 0.405 1.00 0.64 N
ATOM 422 CA TRP A 64 8.711 -2.768 1.865 1.00 0.64 C
ATOM 423 C TRP A 64 7.821 -3.938 2.334 1.00 0.64 C
ATOM 424 O TRP A 64 7.686 -4.945 1.627 1.00 0.64 O
ATOM 425 CB TRP A 64 8.147 -1.426 2.467 1.00 0.64 C
ATOM 426 CG TRP A 64 7.687 -1.325 3.941 1.00 0.64 C
ATOM 427 CD1 TRP A 64 8.484 -1.476 5.033 1.00 0.64 C
ATOM 428 CD2 TRP A 64 6.318 -1.341 4.449 1.00 0.64 C
ATOM 429 NE1 TRP A 64 7.733 -1.546 6.187 1.00 0.64 N
ATOM 430 CE2 TRP A 64 6.402 -1.504 5.848 1.00 0.64 C
ATOM 431 CE3 TRP A 64 5.065 -1.266 3.827 1.00 0.64 C
ATOM 432 CZ2 TRP A 64 5.261 -1.624 6.635 1.00 0.64 C
ATOM 433 CZ3 TRP A 64 3.914 -1.365 4.629 1.00 0.64 C
ATOM 434 CH2 TRP A 64 4.009 -1.552 6.011 1.00 0.64 C
ATOM 435 OXT TRP A 64 7.076 -3.793 3.329 1.00 0.64 O
TER 436 TRP A 64
END

B)

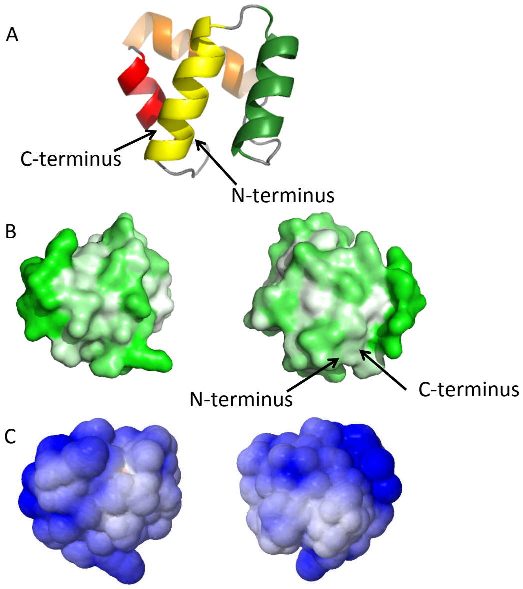

Figure S1- Structural models of pneumocyclicin. A) Ribbon diagram depicting the saposin-like fold. The C- and N- termini are explicitly shown. Hydrophobic surface (B) and electrostatic potential surface (C) maps of pneumocyclicin. Orientation peptide backbone in surface filling diagrams, B and C, correlate to one another.

2. Aureocyclicin

A) PDB file for model of aureocyclicin. Structure modeled after NKR-5-3B using SWISS-MODEL.[84]

```
TITLE     SWISS-MODEL SERVER (https://swissmodel.expasy.org)
TITLE    2 Untitled Project
EXPDTA    THEORETICAL MODEL (SWISS-MODEL SERVER)
AUTHOR    SWISS-MODEL SERVER (SEE REFERENCE IN JRNL
Records)
REVDAT   1   31-OCT-16 1MOD    1       15:49
JRNL       AUTH
M.BIASINI,S.BIENERT,A.WATERHOUSE,K.ARNOLD,G.STUDER,
JRNL       AUTH 2 T.SCHMIDT,F.KIEFER,T.GALLO
CASSARINO,M.BERTONI,L.BORDOLI,
JRNL       AUTH 3 T.SCHWEDE
JRNL       TITL   SWISS-MODEL: MODELLING PROTEIN TERTIARY
AND QUATERNARY
JRNL       TITL 2 STRUCTURE USING EVOLUTIONARY
INFORMATION
JRNL       REF    NUCLEIC.ACIDS.RES                    2014
JRNL       REFN              ISSN 0305-1048
JRNL       PMID   24782522
JRNL       DOI    10.1093/nar/gku340
REMARK   3 MODEL INFORMATION
REMARK   3  ENGIN   PROMOD3
REMARK   3  VERSN   1.0.1
REMARK   3  OSTAT   MONOMER
REMARK   3  OSRSN   USER
REMARK   3  GMQE    0.68
REMARK   3  QMN4    -1.58
REMARK   3  MODT    FALSE
REMARK   3
REMARK   3 TEMPLATE 1
REMARK   3  PDBID   2mp8
REMARK   3  CHAIN   A
REMARK   3  MMCIF   A
REMARK   3  PDBV    2016-10-14
REMARK   3  SMTLE   2mp8.1.A
REMARK   3  SMTLV   2016-10-19
REMARK   3  MTHD    SOLUTION NMR 0.00 A
REMARK   3  FOUND   HHblits
REMARK   3  GMQE    0.68
REMARK   3  SIM     0.38
REMARK   3  SID     36.84
REMARK   3  OSTAT   monomer
ATOM      1  N   LEU A   4       4.118  -7.799   3.715  1.00  0.49           N
ATOM      2  CA  LEU A   4       2.872  -8.607   3.431  1.00  0.49           C
ATOM      3  C   LEU A   4       2.972  -9.947   2.742  1.00  0.49           C
ATOM      4  O   LEU A   4       2.131 -10.807   2.958  1.00  0.49           O
ATOM      5  CB  LEU A   4       1.992  -7.724   2.568  1.00  0.49           C
ATOM      6  CG  LEU A   4       1.872  -6.310   3.139  1.00  0.49           C
ATOM      7  CD1 LEU A   4       1.204  -5.517   2.071  1.00  0.49           C
ATOM      8  CD2 LEU A   4       1.086  -6.190   4.446  1.00  0.49           C
ATOM      9  N   GLY A   5       3.996 -10.151   1.888  1.00  0.58           N
ATOM     10  CA  GLY A   5       4.157 -11.403   1.146  1.00  0.58           C
ATOM     11  C   GLY A   5       3.320 -11.453  -0.109  1.00  0.58           C
ATOM     12  O   GLY A   5       2.771 -12.486  -0.469  1.00  0.58           O
ATOM     13  N   ILE A   6       3.197 -10.304  -0.799  1.00  0.54           N
```

ATOM 14 CA ILE A 6 2.385 -10.148 -1.987 1.00 0.54 C
ATOM 15 C ILE A 6 3.318 -9.942 -3.162 1.00 0.54 C
ATOM 16 O ILE A 6 4.507 -9.684 -2.991 1.00 0.54 O
ATOM 17 CB ILE A 6 1.405 -8.977 -1.903 1.00 0.54 C
ATOM 18 CG1 ILE A 6 2.101 -7.610 -1.685 1.00 0.54 C
ATOM 19 CG2 ILE A 6 0.360 -9.320 -0.822 1.00 0.54 C
ATOM 20 CD1 ILE A 6 1.140 -6.425 -1.804 1.00 0.54 C
ATOM 21 N GLY A 7 2.809 -10.075 -4.405 1.00 0.70 N
ATOM 22 CA GLY A 7 3.583 -9.762 -5.604 1.00 0.70 C
ATOM 23 C GLY A 7 3.844 -8.281 -5.799 1.00 0.70 C
ATOM 24 O GLY A 7 3.115 -7.432 -5.298 1.00 0.70 O
ATOM 25 N THR A 8 4.851 -7.928 -6.622 1.00 0.64 N
ATOM 26 CA THR A 8 5.173 -6.552 -7.002 1.00 0.64 C
ATOM 27 C THR A 8 4.046 -5.864 -7.749 1.00 0.64 C
ATOM 28 O THR A 8 3.789 -4.679 -7.567 1.00 0.64 O
ATOM 29 CB THR A 8 6.436 -6.457 -7.847 1.00 0.64 C
ATOM 30 OG1 THR A 8 6.383 -7.315 -8.984 1.00 0.64 O
ATOM 31 CG2 THR A 8 7.624 -6.924 -6.998 1.00 0.64 C
ATOM 32 N GLY A 9 3.339 -6.627 -8.612 1.00 0.73 N
ATOM 33 CA GLY A 9 2.092 -6.245 -9.272 1.00 0.73 C
ATOM 34 C GLY A 9 0.999 -5.821 -8.337 1.00 0.73 C
ATOM 35 O GLY A 9 0.437 -4.740 -8.468 1.00 0.73 O
ATOM 36 N MET A 10 0.682 -6.663 -7.328 1.00 0.65 N
ATOM 37 CA MET A 10 -0.220 -6.273 -6.264 1.00 0.65 C
ATOM 38 C MET A 10 0.311 -5.066 -5.517 1.00 0.65 C
ATOM 39 O MET A 10 -0.381 -4.066 -5.429 1.00 0.65 O
ATOM 40 CB MET A 10 -0.471 -7.419 -5.263 1.00 0.65 C
ATOM 41 CG MET A 10 -1.242 -8.607 -5.861 1.00 0.65 C
ATOM 42 SD MET A 10 -1.285 -10.058 -4.766 1.00 0.65 S
ATOM 43 CE MET A 10 -2.453 -9.332 -3.584 1.00 0.65 C
ATOM 44 N ALA A 11 1.584 -5.076 -5.072 1.00 0.73 N
ATOM 45 CA ALA A 11 2.165 -3.953 -4.365 1.00 0.73 C
ATOM 46 C ALA A 11 2.070 -2.612 -5.114 1.00 0.73 C
ATOM 47 O ALA A 11 1.596 -1.625 -4.574 1.00 0.73 O
ATOM 48 CB ALA A 11 3.627 -4.276 -4.022 1.00 0.73 C
ATOM 49 N ALA A 12 2.408 -2.549 -6.412 1.00 0.75 N
ATOM 50 CA ALA A 12 2.209 -1.371 -7.244 1.00 0.75 C
ATOM 51 C ALA A 12 0.763 -0.905 -7.406 1.00 0.75 C
ATOM 52 O ALA A 12 0.451 0.286 -7.411 1.00 0.75 O
ATOM 53 CB ALA A 12 2.773 -1.667 -8.637 1.00 0.75 C
ATOM 54 N THR A 13 -0.173 -1.856 -7.527 1.00 0.74 N
ATOM 55 CA THR A 13 -1.603 -1.590 -7.516 1.00 0.74 C
ATOM 56 C THR A 13 -2.084 -0.990 -6.218 1.00 0.74 C
ATOM 57 O THR A 13 -2.882 -0.051 -6.181 1.00 0.74 O
ATOM 58 CB THR A 13 -2.394 -2.849 -7.748 1.00 0.74 C
ATOM 59 OG1 THR A 13 -2.185 -3.329 -9.065 1.00 0.74 O
ATOM 60 CG2 THR A 13 -3.876 -2.534 -7.668 1.00 0.74 C
ATOM 61 N ILE A 14 -1.576 -1.516 -5.099 1.00 0.73 N
ATOM 62 CA ILE A 14 -1.814 -0.951 -3.797 1.00 0.73 C
ATOM 63 C ILE A 14 -1.330 0.493 -3.692 1.00 0.73 C
ATOM 64 O ILE A 14 -2.054 1.377 -3.238 1.00 0.73 O
ATOM 65 CB ILE A 14 -1.045 -1.701 -2.747 1.00 0.73 C
ATOM 66 CG1 ILE A 14 -1.377 -3.201 -2.560 1.00 0.73 C
ATOM 67 CG2 ILE A 14 -1.234 -0.935 -1.445 1.00 0.73 C
ATOM 68 CD1 ILE A 14 -2.747 -3.509 -1.981 1.00 0.73 C
ATOM 69 N ILE A 15 -0.087 0.768 -4.147 1.00 0.71 N
ATOM 70 CA ILE A 15 0.518 2.092 -4.156 1.00 0.71 C
ATOM 71 C ILE A 15 -0.336 3.105 -4.852 1.00 0.71 C
ATOM 72 O ILE A 15 -0.457 4.245 -4.407 1.00 0.71 O
ATOM 73 CB ILE A 15 1.817 2.085 -4.917 1.00 0.71 C
ATOM 74 CG1 ILE A 15 2.913 1.305 -4.204 1.00 0.71 C
ATOM 75 CG2 ILE A 15 2.348 3.464 -5.371 1.00 0.71 C
ATOM 76 CD1 ILE A 15 3.286 1.653 -2.771 1.00 0.71 C
ATOM 77 N ASN A 16 -0.959 2.710 -5.976 1.00 0.74 N
ATOM 78 CA ASN A 16 -1.916 3.565 -6.635 1.00 0.74 C
ATOM 79 C ASN A 16 -3.065 3.960 -5.716 1.00 0.74 C
ATOM 80 O ASN A 16 -3.348 5.134 -5.529 1.00 0.74 O
ATOM 81 CB ASN A 16 -2.467 2.888 -7.905 1.00 0.74 C
ATOM 82 CG ASN A 16 -1.362 2.856 -8.955 1.00 0.74 C
ATOM 83 OD1 ASN A 16 -0.448 3.683 -8.966 1.00 0.74 O
ATOM 84 ND2 ASN A 16 -1.467 1.897 -9.901 1.00 0.74 N
ATOM 85 N ALA A 17 -3.691 2.988 -5.046 1.00 0.77 N
ATOM 86 CA ALA A 17 -4.770 3.208 -4.122 1.00 0.77 C
ATOM 87 C ALA A 17 -4.468 3.986 -2.838 1.00 0.77 C
ATOM 88 O ALA A 17 -5.254 4.845 -2.447 1.00 0.77 O
ATOM 89 CB ALA A 17 -5.375 1.830 -3.852 1.00 0.77 C
ATOM 90 N ILE A 18 -3.323 3.731 -2.165 1.00 0.70 N
ATOM 91 CA ILE A 18 -2.878 4.501 -0.996 1.00 0.70 C
ATOM 92 C ILE A 18 -2.452 5.922 -1.332 1.00 0.70 C
ATOM 93 O ILE A 18 -2.525 6.820 -0.496 1.00 0.70 O
ATOM 94 CB ILE A 18 -1.738 3.864 -0.191 1.00 0.70 C
ATOM 95 CG1 ILE A 18 -0.446 3.710 -1.015 1.00 0.70 C
ATOM 96 CG2 ILE A 18 -2.181 2.504 0.363 1.00 0.70 C
ATOM 97 CD1 ILE A 18 0.744 3.136 -0.248 1.00 0.70 C
ATOM 98 N SER A 19 -1.945 6.120 -2.566 1.00 0.72 N
ATOM 99 CA SER A 19 -1.619 7.417 -3.141 1.00 0.72 C
ATOM 100 C SER A 19 -2.824 8.231 -3.581 1.00 0.72 C
ATOM 101 O SER A 19 -2.831 9.449 -3.457 1.00 0.72 O
ATOM 102 CB SER A 19 -0.777 7.277 -4.442 1.00 0.72 C
ATOM 103 OG SER A 19 0.523 6.709 -4.255 1.00 0.72 O
ATOM 104 N VAL A 20 -3.837 7.582 -4.190 1.00 0.63 N
ATOM 105 CA VAL A 20 -5.060 8.228 -4.666 1.00 0.63 C
ATOM 106 C VAL A 20 -6.043 8.490 -3.539 1.00 0.63 C
ATOM 107 O VAL A 20 -6.636 9.562 -3.420 1.00 0.63 O
ATOM 108 CB VAL A 20 -5.749 7.390 -5.753 1.00 0.63 C
ATOM 109 CG1 VAL A 20 -7.114 7.977 -6.165 1.00 0.63 C
ATOM 110 CG2 VAL A 20 -4.862 7.367 -7.012 1.00 0.63 C
ATOM 111 N GLY A 21 -6.269 7.485 -2.672 1.00 0.68 N
ATOM 112 CA GLY A 21 -7.202 7.605 -1.570 1.00 0.68 C
ATOM 113 C GLY A 21 -6.700 8.483 -0.444 1.00 0.68 C
ATOM 114 O GLY A 21 -5.519 8.533 -0.117 1.00 0.68 O
ATOM 115 N LEU A 22 -7.627 9.177 0.239 1.00 0.51 N
ATOM 116 CA LEU A 22 -7.282 10.136 1.272 1.00 0.51 C
ATOM 117 C LEU A 22 -7.477 9.541 2.657 1.00 0.51 C
ATOM 118 O LEU A 22 -6.519 9.218 3.364 1.00 0.51 O
ATOM 119 CB LEU A 22 -8.162 11.402 1.142 1.00 0.51 C
ATOM 120 CG LEU A 22 -7.900 12.224 -0.132 1.00 0.51 C
ATOM 121 CD1 LEU A 22 -8.936 13.354 -0.221 1.00 0.51
C
ATOM 122 CD2 LEU A 22 -6.469 12.781 -0.187 1.00 0.51
C
ATOM 123 N SER A 23 -8.752 9.391 3.073 1.00 0.59 N
ATOM 124 CA SER A 23 -9.172 8.796 4.341 1.00 0.59 C
ATOM 125 C SER A 23 -8.710 7.347 4.472 1.00 0.59 C
ATOM 126 O SER A 23 -8.851 6.556 3.544 1.00 0.59 O
ATOM 127 CB SER A 23 -10.720 8.839 4.505 1.00 0.59 C
ATOM 128 OG SER A 23 -11.167 8.248 5.729 1.00 0.59 O
ATOM 129 N ALA A 24 -8.155 6.952 5.638 1.00 0.70 N
ATOM 130 CA ALA A 24 -7.600 5.631 5.863 1.00 0.70 C
ATOM 131 C ALA A 24 -8.601 4.486 5.716 1.00 0.70 C
ATOM 132 O ALA A 24 -8.313 3.470 5.093 1.00 0.70 O
ATOM 133 CB ALA A 24 -6.973 5.608 7.268 1.00 0.70 C
ATOM 134 N ALA A 25 -9.828 4.657 6.245 1.00 0.72 N
ATOM 135 CA ALA A 25 -10.919 3.709 6.114 1.00 0.72 C
ATOM 136 C ALA A 25 -11.326 3.485 4.659 1.00 0.72 C
ATOM 137 O ALA A 25 -11.548 2.359 4.218 1.00 0.72 O
ATOM 138 CB ALA A 25 -12.126 4.222 6.925 1.00 0.72 C
ATOM 139 N THR A 26 -11.379 4.581 3.867 1.00 0.68 N
ATOM 140 CA THR A 26 -11.651 4.550 2.428 1.00 0.68 C
ATOM 141 C THR A 26 -10.598 3.768 1.672 1.00 0.68 C
ATOM 142 O THR A 26 -10.906 2.894 0.866 1.00 0.68 O
ATOM 143 CB THR A 26 -11.725 5.949 1.811 1.00 0.68 C
ATOM 144 OG1 THR A 26 -12.708 6.738 2.467 1.00 0.68
O
ATOM 145 CG2 THR A 26 -12.120 5.936 0.327 1.00 0.68
C
ATOM 146 N ILE A 27 -9.304 4.025 1.961 1.00 0.67 N
ATOM 147 CA ILE A 27 -8.198 3.291 1.373 1.00 0.67 C

ATOM 148 C ILE A 27 -8.231 1.812 1.771 1.00 0.67 C
ATOM 149 O ILE A 27 -8.084 0.949 0.916 1.00 0.67 O
ATOM 150 CB ILE A 27 -6.833 3.904 1.695 1.00 0.67 C
ATOM 151 CG1 ILE A 27 -6.705 5.399 1.350 1.00 0.67 C
ATOM 152 CG2 ILE A 27 -5.745 3.202 0.873 1.00 0.67 C
ATOM 153 CD1 ILE A 27 -5.533 6.083 2.066 1.00 0.67 C
ATOM 154 N LEU A 28 -8.501 1.461 3.053 1.00 0.72 N
ATOM 155 CA LEU A 28 -8.553 0.065 3.507 1.00 0.72 C
ATOM 156 C LEU A 28 -9.498 -0.809 2.705 1.00 0.72 C
ATOM 157 O LEU A 28 -9.149 -1.892 2.242 1.00 0.72 O
ATOM 158 CB LEU A 28 -9.081 -0.034 4.959 1.00 0.72 C
ATOM 159 CG LEU A 28 -8.994 -1.432 5.617 1.00 0.72 C
ATOM 160 CD1 LEU A 28 -7.615 -2.109 5.539 1.00 0.72 C
ATOM 161 CD2 LEU A 28 -9.469 -1.337 7.072 1.00 0.72 C
ATOM 162 N SER A 29 -10.723 -0.294 2.509 1.00 0.79 N
ATOM 163 CA SER A 29 -11.780 -0.887 1.711 1.00 0.79 C
ATOM 164 C SER A 29 -11.454 -1.055 0.240 1.00 0.79 C
ATOM 165 O SER A 29 -11.790 -2.062 -0.375 1.00 0.79 O
ATOM 166 CB SER A 29 -13.068 -0.045 1.803 1.00 0.79 C
ATOM 167 OG SER A 29 -13.572 -0.046 3.140 1.00 0.79 O
ATOM 168 N LEU A 30 -10.793 -0.061 -0.386 1.00 0.73 N
ATOM 169 CA LEU A 30 -10.335 -0.189 -1.760 1.00 0.73 C
ATOM 170 C LEU A 30 -9.206 -1.179 -1.910 1.00 0.73 C
ATOM 171 O LEU A 30 -9.209 -2.041 -2.784 1.00 0.73 O
ATOM 172 CB LEU A 30 -9.866 1.164 -2.327 1.00 0.73 C
ATOM 173 CG LEU A 30 -10.981 2.220 -2.380 1.00 0.73 C
ATOM 174 CD1 LEU A 30 -10.411 3.556 -2.870 1.00 0.73
C
ATOM 175 CD2 LEU A 30 -12.202 1.776 -3.203 1.00 0.73
C
ATOM 176 N ILE A 31 -8.218 -1.107 -1.010 1.00 0.71 N
ATOM 177 CA ILE A 31 -7.112 -2.045 -0.983 1.00 0.71 C
ATOM 178 C ILE A 31 -7.556 -3.483 -0.780 1.00 0.71 C
ATOM 179 O ILE A 31 -7.117 -4.403 -1.475 1.00 0.71 O
ATOM 180 CB ILE A 31 -6.154 -1.693 0.132 1.00 0.71 C
ATOM 181 CG1 ILE A 31 -5.413 -0.368 -0.065 1.00 0.71 C
ATOM 182 CG2 ILE A 31 -5.143 -2.826 0.333 1.00 0.71 C
ATOM 183 CD1 ILE A 31 -4.679 -0.354 -1.388 1.00 0.71 C
ATOM 184 N SER A 32 -8.463 -3.716 0.179 1.00 0.77 N
ATOM 185 CA SER A 32 -8.951 -5.043 0.483 1.00 0.77 C
ATOM 186 C SER A 32 -9.691 -5.696 -0.666 1.00 0.77 C
ATOM 187 O SER A 32 -9.499 -6.880 -0.942 1.00 0.77 O
ATOM 188 CB SER A 32 -9.793 -5.064 1.784 1.00 0.77 C
ATOM 189 OG SER A 32 -11.033 -4.375 1.640 1.00 0.77 O
ATOM 190 N GLY A 33 -10.501 -4.890 -1.385 1.00 0.76 N
ATOM 191 CA GLY A 33 -11.214 -5.274 -2.596 1.00 0.76
C
ATOM 192 C GLY A 33 -10.350 -5.622 -3.781 1.00 0.76 C
ATOM 193 O GLY A 33 -10.717 -6.446 -4.610 1.00 0.76 O
ATOM 194 N VAL A 34 -9.174 -4.987 -3.898 1.00 0.71 N
ATOM 195 CA VAL A 34 -8.213 -5.277 -4.944 1.00 0.71 C
ATOM 196 C VAL A 34 -7.286 -6.456 -4.651 1.00 0.71 C
ATOM 197 O VAL A 34 -7.078 -7.334 -5.485 1.00 0.71 O
ATOM 198 CB VAL A 34 -7.358 -4.049 -5.154 1.00 0.71 C
ATOM 199 CG1 VAL A 34 -6.233 -4.343 -6.144 1.00 0.71
C
ATOM 200 CG2 VAL A 34 -8.187 -2.906 -5.763 1.00 0.71
C
ATOM 201 N ALA A 35 -6.667 -6.504 -3.451 1.00 0.73 N
ATOM 202 CA ALA A 35 -5.713 -7.544 -3.108 1.00 0.73 C
ATOM 203 C ALA A 35 -6.373 -8.851 -2.667 1.00 0.73 C
ATOM 204 O ALA A 35 -5.713 -9.875 -2.521 1.00 0.73 O
ATOM 205 CB ALA A 35 -4.765 -7.053 -1.993 1.00 0.73 C
ATOM 206 N SER A 36 -7.701 -8.819 -2.426 1.00 0.60 N
ATOM 207 CA SER A 36 -8.521 -9.954 -2.014 1.00 0.60 C
ATOM 208 C SER A 36 -8.133 -10.504 -0.655 1.00 0.60 C
ATOM 209 O SER A 36 -8.057 -11.705 -0.418 1.00 0.60 O
ATOM 210 CB SER A 36 -8.662 -11.081 -3.069 1.00 0.60 C
ATOM 211 OG SER A 36 -9.266 -10.587 -4.270 1.00 0.60 O
ATOM 212 N GLY A 37 -7.901 -9.597 0.311 1.00 0.61 N
ATOM 213 CA GLY A 37 -7.475 -10.018 1.637 1.00 0.61 C
ATOM 214 C GLY A 37 -7.354 -8.877 2.595 1.00 0.61 C
ATOM 215 O GLY A 37 -7.553 -9.034 3.791 1.00 0.61 O
ATOM 216 N GLY A 38 -7.015 -7.662 2.106 1.00 0.69 N
ATOM 217 CA GLY A 38 -6.928 -6.482 2.973 1.00 0.69 C
ATOM 218 C GLY A 38 -5.725 -6.473 3.853 1.00 0.69 C
ATOM 219 O GLY A 38 -5.593 -5.664 4.764 1.00 0.69 O
ATOM 220 N ALA A 39 -4.793 -7.387 3.559 1.00 0.65 N
ATOM 221 CA ALA A 39 -3.497 -7.447 4.168 1.00 0.65 C
ATOM 222 C ALA A 39 -2.645 -6.339 3.588 1.00 0.65 C
ATOM 223 O ALA A 39 -1.895 -6.544 2.628 1.00 0.65 O
ATOM 224 CB ALA A 39 -2.892 -8.858 4.003 1.00 0.65 C
ATOM 225 N TRP A 40 -2.801 -5.112 4.113 1.00 0.68 N
ATOM 226 CA TRP A 40 -2.002 -3.974 3.732 1.00 0.68 C
ATOM 227 C TRP A 40 -1.921 -2.976 4.880 1.00 0.68 C
ATOM 228 O TRP A 40 -2.880 -2.749 5.614 1.00 0.68 O
ATOM 229 CB TRP A 40 -2.506 -3.277 2.443 1.00 0.68 C
ATOM 230 CG TRP A 40 -1.622 -2.121 2.018 1.00 0.68 C
ATOM 231 CD1 TRP A 40 -1.788 -0.792 2.298 1.00 0.68 C
ATOM 232 CD2 TRP A 40 -0.386 -2.225 1.325 1.00 0.68 C
ATOM 233 NE1 TRP A 40 -0.671 -0.091 1.927 1.00 0.68 N
ATOM 234 CE2 TRP A 40 0.209 -0.940 1.338 1.00 0.68 C
ATOM 235 CE3 TRP A 40 0.193 -3.242 0.574 1.00 0.68 C
ATOM 236 CZ2 TRP A 40 1.415 -0.711 0.704 1.00 0.68 C
ATOM 237 CZ3 TRP A 40 1.378 -2.980 -0.133 1.00 0.68 C
ATOM 238 CH2 TRP A 40 2.001 -1.735 -0.053 1.00 0.68 C
ATOM 239 N VAL A 41 -0.751 -2.326 5.034 1.00 0.76 N
ATOM 240 CA VAL A 41 -0.508 -1.298 6.036 1.00 0.76 C
ATOM 241 C VAL A 41 -0.789 0.088 5.482 1.00 0.76 C
ATOM 242 O VAL A 41 -0.023 0.632 4.706 1.00 0.76 O
ATOM 243 CB VAL A 41 0.960 -1.257 6.442 1.00 0.76 C
ATOM 244 CG1 VAL A 41 1.253 -0.021 7.330 1.00 0.76 C
ATOM 245 CG2 VAL A 41 1.304 -2.555 7.186 1.00 0.76 C
ATOM 246 N LEU A 42 -1.869 0.770 5.885 1.00 0.72 N
ATOM 247 CA LEU A 42 -2.168 2.024 5.220 1.00 0.72 C
ATOM 248 C LEU A 42 -1.383 3.255 5.608 1.00 0.72 C
ATOM 249 O LEU A 42 -0.790 3.945 4.782 1.00 0.72 O
ATOM 250 CB LEU A 42 -3.596 2.354 5.600 1.00 0.72 C
ATOM 251 CG LEU A 42 -4.567 1.315 5.072 1.00 0.72 C
ATOM 252 CD1 LEU A 42 -5.897 1.636 5.711 1.00 0.72 C
ATOM 253 CD2 LEU A 42 -4.695 1.459 3.561 1.00 0.72 C
ATOM 254 N ALA A 43 -1.403 3.547 6.918 1.00 0.78 N
ATOM 255 CA ALA A 43 -0.800 4.702 7.535 1.00 0.78 C
ATOM 256 C ALA A 43 0.712 4.707 7.367 1.00 0.78 C
ATOM 257 O ALA A 43 1.315 5.716 7.016 1.00 0.78 O
ATOM 258 CB ALA A 43 -1.210 4.718 9.021 1.00 0.78 C
ATOM 259 N GLY A 44 1.341 3.528 7.552 1.00 0.82 N
ATOM 260 CA GLY A 44 2.772 3.343 7.357 1.00 0.82 C
ATOM 261 C GLY A 44 3.212 3.373 5.912 1.00 0.82 C
ATOM 262 O GLY A 44 4.246 3.944 5.590 1.00 0.82 O
ATOM 263 N ALA A 45 2.435 2.780 4.981 1.00 0.79 N
ATOM 264 CA ALA A 45 2.753 2.863 3.574 1.00 0.79 C
ATOM 265 C ALA A 45 2.650 4.273 3.007 1.00 0.79 C
ATOM 266 O ALA A 45 3.576 4.748 2.361 1.00 0.79 O
ATOM 267 CB ALA A 45 1.851 1.913 2.790 1.00 0.79 C
ATOM 268 N LYS A 46 1.565 5.032 3.287 1.00 0.74 N
ATOM 269 CA LYS A 46 1.465 6.409 2.821 1.00 0.74 C
ATOM 270 C LYS A 46 2.548 7.342 3.374 1.00 0.74 C
ATOM 271 O LYS A 46 3.019 8.252 2.694 1.00 0.74 O
ATOM 272 CB LYS A 46 0.054 7.023 3.003 1.00 0.74 C
ATOM 273 CG LYS A 46 -0.377 7.320 4.446 1.00 0.74 C
ATOM 274 CD LYS A 46 -1.561 8.300 4.508 1.00 0.74 C
ATOM 275 CE LYS A 46 -2.854 7.778 3.874 1.00 0.74 C
ATOM 276 NZ LYS A 46 -3.837 8.876 3.776 1.00 0.74 N
ATOM 277 N GLN A 47 2.971 7.118 4.637 1.00 0.74 N
ATOM 278 CA GLN A 47 4.105 7.783 5.254 1.00 0.74 C
ATOM 279 C GLN A 47 5.442 7.415 4.610 1.00 0.74 C
ATOM 280 O GLN A 47 6.256 8.277 4.286 1.00 0.74 O

ATOM 281 CB GLN A 47 4.127 7.493 6.774 1.00 0.74 C
ATOM 282 CG GLN A 47 5.126 8.355 7.579 1.00 0.74 C
ATOM 283 CD GLN A 47 4.814 9.843 7.414 1.00 0.74 C
ATOM 284 OE1 GLN A 47 3.660 10.277 7.469 1.00 0.74
O
ATOM 285 NE2 GLN A 47 5.862 10.665 7.187 1.00 0.74
N
ATOM 286 N ALA A 48 5.666 6.113 4.320 1.00 0.81 N
ATOM 287 CA ALA A 48 6.814 5.628 3.568 1.00 0.81 C
ATOM 288 C ALA A 48 6.899 6.227 2.168 1.00 0.81 C
ATOM 289 O ALA A 48 7.963 6.580 1.664 1.00 0.81 O
ATOM 290 CB ALA A 48 6.771 4.088 3.465 1.00 0.81 C
ATOM 291 N LEU A 49 5.744 6.388 1.516 1.00 0.74 N
ATOM 292 CA LEU A 49 5.593 7.077 0.252 1.00 0.74 C
ATOM 293 C LEU A 49 5.887 8.542 0.254 1.00 0.74 C
ATOM 294 O LEU A 49 6.332 9.107 -0.742 1.00 0.74 O
ATOM 295 CB LEU A 49 4.188 6.863 -0.292 1.00 0.74 C
ATOM 296 CG LEU A 49 4.196 5.547 -1.081 1.00 0.74 C
ATOM 297 CD1 LEU A 49 4.631 4.221 -0.421 1.00 0.74 C
ATOM 298 CD2 LEU A 49 2.915 5.398 -1.872 1.00 0.74 C
ATOM 299 N LYS A 50 5.589 9.194 1.372 1.00 0.77 N
ATOM 300 CA LYS A 50 5.925 10.566 1.580 1.00 0.77 C
ATOM 301 C LYS A 50 7.420 10.835 1.754 1.00 0.77 C
ATOM 302 O LYS A 50 7.946 11.785 1.183 1.00 0.77 O
ATOM 303 CB LYS A 50 5.131 11.038 2.810 1.00 0.77 C
ATOM 304 CG LYS A 50 5.307 12.524 3.128 1.00 0.77 C
ATOM 305 CD LYS A 50 4.861 13.463 1.995 1.00 0.77 C
ATOM 306 CE LYS A 50 3.373 13.424 1.660 1.00 0.77 C
ATOM 307 NZ LYS A 50 2.632 13.923 2.831 1.00 0.77 N
ATOM 308 N GLU A 51 8.126 10.016 2.560 1.00 0.75 N
ATOM 309 CA GLU A 51 9.550 10.196 2.802 1.00 0.75 C
ATOM 310 C GLU A 51 10.480 9.509 1.807 1.00 0.75 C
ATOM 311 O GLU A 51 11.404 10.119 1.275 1.00 0.75 O
ATOM 312 CB GLU A 51 9.888 9.699 4.220 1.00 0.75 C
ATOM 313 CG GLU A 51 9.269 10.607 5.305 1.00 0.75 C
ATOM 314 CD GLU A 51 9.524 10.061 6.706 1.00 0.75 C
ATOM 315 OE1 GLU A 51 10.714 9.844 7.045 1.00 0.75
O
ATOM 316 OE2 GLU A 51 8.517 9.862 7.439 1.00 0.75 O
ATOM 317 N GLY A 52 10.273 8.201 1.540 1.00 0.87 N
ATOM 318 CA GLY A 52 11.160 7.402 0.692 1.00 0.87 C
ATOM 319 C GLY A 52 10.670 7.229 -0.729 1.00 0.87 C
ATOM 320 O GLY A 52 11.456 7.115 -1.667 1.00 0.87 O
ATOM 321 N GLY A 53 9.334 7.190 -0.921 1.00 0.84 N
ATOM 322 CA GLY A 53 8.691 7.201 -2.236 1.00 0.84 C
ATOM 323 C GLY A 53 7.866 5.986 -2.579 1.00 0.84 C
ATOM 324 O GLY A 53 7.922 4.945 -1.928 1.00 0.84 O
ATOM 325 N LYS A 54 7.079 6.086 -3.683 1.00 0.69 N
ATOM 326 CA LYS A 54 6.192 5.037 -4.187 1.00 0.69 C
ATOM 327 C LYS A 54 6.879 3.707 -4.357 1.00 0.69 C
ATOM 328 O LYS A 54 6.434 2.690 -3.846 1.00 0.69 O
ATOM 329 CB LYS A 54 5.494 5.493 -5.488 1.00 0.69 C
ATOM 330 CG LYS A 54 4.505 6.624 -5.168 1.00 0.69 C
ATOM 331 CD LYS A 54 3.761 7.159 -6.403 1.00 0.69 C
ATOM 332 CE LYS A 54 2.825 8.335 -6.103 1.00 0.69 C
ATOM 333 NZ LYS A 54 2.072 8.722 -7.318 1.00 0.69 N
ATOM 334 N LYS A 55 8.052 3.708 -4.994 1.00 0.64 N
ATOM 335 CA LYS A 55 8.842 2.524 -5.188 1.00 0.64 C
ATOM 336 C LYS A 55 9.469 1.896 -3.927 1.00 0.64 C
ATOM 337 O LYS A 55 9.719 0.695 -3.881 1.00 0.64 O
ATOM 338 CB LYS A 55 9.889 2.900 -6.236 1.00 0.64 C
ATOM 339 CG LYS A 55 10.609 1.686 -6.811 1.00 0.64 C
ATOM 340 CD LYS A 55 11.589 2.083 -7.915 1.00 0.64 C
ATOM 341 CE LYS A 55 12.708 2.986 -7.397 1.00 0.64 C
ATOM 342 NZ LYS A 55 13.658 3.280 -8.485 1.00 0.64 N
ATOM 343 N ALA A 56 9.735 2.683 -2.860 1.00 0.69 N
ATOM 344 CA ALA A 56 10.171 2.160 -1.577 1.00 0.69 C
ATOM 345 C ALA A 56 9.072 1.367 -0.864 1.00 0.69 C
ATOM 346 O ALA A 56 9.297 0.283 -0.335 1.00 0.69 O
ATOM 347 CB ALA A 56 10.647 3.332 -0.697 1.00 0.69 C
ATOM 348 N GLY A 57 7.828 1.894 -0.873 1.00 0.73 N
ATOM 349 CA GLY A 57 6.690 1.213 -0.262 1.00 0.73 C
ATOM 350 C GLY A 57 6.098 0.061 -1.070 1.00 0.73 C
ATOM 351 O GLY A 57 5.291 -0.688 -0.547 1.00 0.73 O
ATOM 352 N ILE A 58 6.474 -0.073 -2.376 1.00 0.60 N
ATOM 353 CA ILE A 58 6.275 -1.259 -3.244 1.00 0.60 C
ATOM 354 C ILE A 58 7.048 -2.472 -2.765 1.00 0.60 C
ATOM 355 O ILE A 58 6.581 -3.605 -2.809 1.00 0.60 O
ATOM 356 CB ILE A 58 6.688 -1.036 -4.725 1.00 0.60 C
ATOM 357 CG1 ILE A 58 5.666 -0.091 -5.355 1.00 0.60 C
ATOM 358 CG2 ILE A 58 6.668 -2.340 -5.557 1.00 0.60 C
ATOM 359 CD1 ILE A 58 5.847 0.438 -6.782 1.00 0.60 C
ATOM 360 N ALA A 59 8.310 -2.225 -2.375 1.00 0.62 N
ATOM 361 CA ALA A 59 9.219 -3.234 -1.890 1.00 0.62 C
ATOM 362 C ALA A 59 8.973 -3.659 -0.431 1.00 0.62 C
ATOM 363 O ALA A 59 9.284 -4.783 -0.050 1.00 0.62 O
ATOM 364 CB ALA A 59 10.656 -2.705 -2.079 1.00 0.62 C
ATOM 365 N PHE A 60 8.430 -2.738 0.393 1.00 0.53 N
ATOM 366 CA PHE A 60 8.005 -2.952 1.771 1.00 0.53 C
ATOM 367 C PHE A 60 6.703 -3.830 1.953 1.00 0.53 C
ATOM 368 O PHE A 60 5.922 -4.011 0.980 1.00 0.53 O
ATOM 369 CB PHE A 60 7.914 -1.519 2.392 1.00 0.53 C
ATOM 370 CG PHE A 60 7.623 -1.510 3.865 1.00 0.53 C
ATOM 371 CD1 PHE A 60 8.612 -1.813 4.817 1.00 0.53 C
ATOM 372 CD2 PHE A 60 6.315 -1.253 4.298 1.00 0.53 C
ATOM 373 CE1 PHE A 60 8.272 -1.935 6.173 1.00 0.53 C
ATOM 374 CE2 PHE A 60 5.973 -1.377 5.648 1.00 0.53 C
ATOM 375 CZ PHE A 60 6.950 -1.729 6.586 1.00 0.53 C
ATOM 376 OXT PHE A 60 6.499 -4.371 3.082 1.00 0.53 O
TER 377 PHE A 60
END

B)

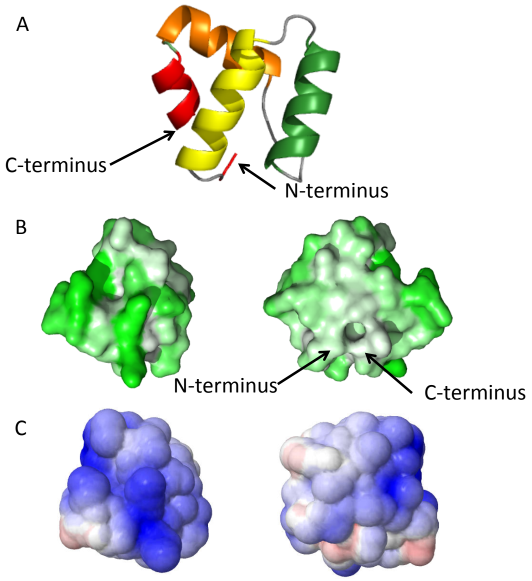

Figure S2- Structural models of aureocyclicin. A) Ribbon diagram depicting the saposin-like fold. The C- and N- termini are explicitly shown. Hydrophobic surface (B) and electrostatic potential surface (C) maps of aureocyclicin. Orientation peptide backbone in the space filling diagrams, B and C, correlate to one another.

3) Amylocyclicin

A) PDB file for model of amylocyclicin. Structure modeled after NKR-5-3B using SWISS-MODEL[84]

```
TITLE     SWISS-MODEL SERVER (https://swissmodel.expasy.org)
TITLE    2 Untitled Project
EXPDTA    THEORETICAL MODEL (SWISS-MODEL SERVER)
AUTHOR    SWISS-MODEL SERVER (SEE REFERENCE IN JRNL
Records)
REVDAT   1   31-OCT-16 1MOD    1       16:08
JRNL        AUTH
M.BIASINI,S.BIENERT,A.WATERHOUSE,K.ARNOLD,G.STUDER,
JRNL        AUTH 2 T.SCHMIDT,F.KIEFER,T.GALLO
CASSARINO,M.BERTONI,L.BORDOLI,
JRNL        AUTH 3 T.SCHWEDE
JRNL        TITL   SWISS-MODEL: MODELLING PROTEIN TERTIARY
AND QUATERNARY
JRNL        TITL 2 STRUCTURE USING EVOLUTIONARY
INFORMATION
JRNL        REF    NUCLEIC.ACIDS.RES                        2014
JRNL        REFN                  ISSN 0305-1048
JRNL        PMID   24782522
JRNL        DOI    10.1093/nar/gku340
REMARK   3
REMARK   3 MODEL INFORMATION
REMARK   3  ENGIN   PROMOD3
REMARK   3  VERSN   1.0.1
REMARK   3  OSTAT   MONOMER
REMARK   3  OSRSN   USER
REMARK   3  GMQE    0.83
REMARK   3  QMN4    -0.46
REMARK   3  MODT    FALSE
REMARK   3
REMARK   3 TEMPLATE 1
REMARK   3  PDBID   2mp8
REMARK   3  CHAIN   A
REMARK   3  MMCIF   A
REMARK   3  PDBV    2016-10-14
REMARK   3  SMTLE   2mp8.1.A
REMARK   3  SMTLV   2016-10-19
REMARK   3  MTHD    SOLUTION NMR 0.00 A
REMARK   3  FOUND   HHblits
REMARK   3  GMQE    0.83
REMARK   3  SIM     0.47
REMARK   3  SID     60.94
REMARK   3  OSTAT   monomer
```

```
ATOM      1 N   LEU A   1       5.371  -3.112   0.968  1.00  0.75           N
ATOM      2 CA  LEU A   1       4.212  -3.996   0.837  1.00  0.75           C
ATOM      3 C   LEU A   1       4.587  -5.410   0.480  1.00  0.75           C
ATOM      4 O   LEU A   1       4.199  -6.345   1.174  1.00  0.75           O
ATOM      5 CB  LEU A   1       3.359  -3.235  -0.189  1.00  0.75           C
ATOM      6 CG  LEU A   1       2.915  -1.821   0.249  1.00  0.75           C
ATOM      7 CD1 LEU A   1       2.267  -1.163  -0.971  1.00  0.75           C
ATOM      8 CD2 LEU A   1       1.987  -1.831   1.477  1.00  0.75           C
ATOM      9 N   ALA A   2       5.401  -5.638  -0.558  1.00  0.74           N
ATOM     10 CA  ALA A   2       5.742  -6.974  -0.966  1.00  0.74           C
ATOM     11 C   ALA A   2       6.433  -7.882   0.062  1.00  0.74           C
ATOM     12 O   ALA A   2       6.006  -9.032   0.246  1.00  0.74           O
ATOM     13 CB  ALA A   2       6.619  -6.845  -2.215  1.00  0.74           C
ATOM     14 N   SER A   3       7.459  -7.379   0.768  1.00  0.72           N
ATOM     15 CA  SER A   3       8.200  -8.002   1.862  1.00  0.72           C
ATOM     16 C   SER A   3       7.372  -8.176   3.127  1.00  0.72           C
ATOM     17 O   SER A   3       7.418  -9.235   3.742  1.00  0.72           O
ATOM     18 CB  SER A   3       9.465  -7.206   2.311  1.00  0.72           C
ATOM     19 OG  SER A   3      10.534  -7.167   1.355  1.00  0.72           O
ATOM     20 N   THR A   4       6.606  -7.148   3.586  1.00  0.71           N
ATOM     21 CA  THR A   4       5.831  -7.267   4.836  1.00  0.71           C
ATOM     22 C   THR A   4       4.529  -8.020   4.714  1.00  0.71           C
ATOM     23 O   THR A   4       4.101  -8.708   5.639  1.00  0.71           O
ATOM     24 CB  THR A   4       5.500  -5.973   5.573  1.00  0.71           C
ATOM     25 OG1 THR A   4       4.725  -5.054   4.797  1.00  0.71           O
ATOM     26 CG2 THR A   4       6.809  -5.290   5.972  1.00  0.71           C
ATOM     27 N   LEU A   5       3.852  -7.887   3.564  1.00  0.71           N
ATOM     28 CA  LEU A   5       2.565  -8.502   3.331  1.00  0.71           C
ATOM     29 C   LEU A   5       2.705  -9.846   2.651  1.00  0.71           C
ATOM     30 O   LEU A   5       1.805 -10.680   2.710  1.00  0.71           O
ATOM     31 CB  LEU A   5       1.709  -7.611   2.400  1.00  0.71           C
ATOM     32 CG  LEU A   5       1.529  -6.160   2.872  1.00  0.71           C
ATOM     33 CD1 LEU A   5       0.927  -5.332   1.734  1.00  0.71           C
ATOM     34 CD2 LEU A   5       0.683  -6.068   4.144  1.00  0.71           C
ATOM     35 N   GLY A   6       3.851 -10.088   1.981  1.00  0.78           N
ATOM     36 CA  GLY A   6       4.099 -11.320   1.242  1.00  0.78           C
ATOM     37 C   GLY A   6       3.324 -11.389  -0.045  1.00  0.78           C
ATOM     38 O   GLY A   6       2.790 -12.428  -0.423  1.00  0.78           O
ATOM     39 N   ILE A   7       3.240 -10.246  -0.746  1.00  0.73           N
ATOM     40 CA  ILE A   7       2.450 -10.081  -1.948  1.00  0.73           C
ATOM     41 C   ILE A   7       3.368  -9.880  -3.128  1.00  0.73           C
ATOM     42 O   ILE A   7       4.570  -9.660  -3.004  1.00  0.73           O
ATOM     43 CB  ILE A   7       1.435  -8.933  -1.894  1.00  0.73           C
ATOM     44 CG1 ILE A   7       2.102  -7.556  -1.666  1.00  0.73           C
ATOM     45 CG2 ILE A   7       0.378  -9.290  -0.828  1.00  0.73           C
ATOM     46 CD1 ILE A   7       1.132  -6.374  -1.742  1.00  0.73           C
ATOM     47 N   SER A   8       2.810  -9.966  -4.346  1.00  0.81           N
ATOM     48 CA  SER A   8       3.535  -9.702  -5.573  1.00  0.81           C
ATOM     49 C   SER A   8       3.849  -8.228  -5.758  1.00  0.81           C
ATOM     50 O   SER A   8       3.209  -7.347  -5.187  1.00  0.81           O
ATOM     51 CB  SER A   8       2.770 -10.218  -6.823  1.00  0.81           C
ATOM     52 OG  SER A   8       1.511  -9.559  -7.009  1.00  0.81           O
ATOM     53 N   THR A   9       4.848  -7.905  -6.599  1.00  0.76           N
ATOM     54 CA  THR A   9       5.168  -6.533  -6.984  1.00  0.76           C
ATOM     55 C   THR A   9       4.030  -5.873  -7.746  1.00  0.76           C
ATOM     56 O   THR A   9       3.795  -4.670  -7.637  1.00  0.76           O
ATOM     57 CB  THR A   9       6.430  -6.440  -7.828  1.00  0.76           C
ATOM     58 OG1 THR A   9       6.357  -7.276  -8.977  1.00  0.76           O
ATOM     59 CG2 THR A   9       7.628  -6.929  -7.007  1.00  0.76           C
ATOM     60 N   ALA A  10       3.273  -6.678  -8.525  1.00  0.82           N
ATOM     61 CA  ALA A  10       2.067  -6.271  -9.208  1.00  0.82           C
ATOM     62 C   ALA A  10       0.986  -5.768  -8.261  1.00  0.82           C
ATOM     63 O   ALA A  10       0.487  -4.653  -8.411  1.00  0.82           O
ATOM     64 CB  ALA A  10       1.494  -7.470  -9.995  1.00  0.82           C
ATOM     65 N   ALA A  11       0.659  -6.567  -7.221  1.00  0.84           N
ATOM     66 CA  ALA A  11      -0.270  -6.225  -6.168  1.00  0.84           C
ATOM     67 C   ALA A  11       0.226  -5.083  -5.310  1.00  0.84           C
ATOM     68 O   ALA A  11      -0.547  -4.207  -4.925  1.00  0.84           O
ATOM     69 CB  ALA A  11      -0.534  -7.444  -5.269  1.00  0.84           C
ATOM     70 N   ALA A  12       1.539  -5.046  -5.005  1.00  0.83           N
ATOM     71 CA  ALA A  12       2.150  -3.948  -4.294  1.00  0.83           C
ATOM     72 C   ALA A  12       2.018  -2.626  -5.031  1.00  0.83           C
ATOM     73 O   ALA A  12       1.532  -1.651  -4.471  1.00  0.83           O
ATOM     74 CB  ALA A  12       3.638  -4.246  -4.029  1.00  0.83           C
ATOM     75 N   LYS A  13       2.348  -2.574  -6.333  1.00  0.78           N
ATOM     76 CA  LYS A  13       2.183  -1.397  -7.169  1.00  0.78           C
ATOM     77 C   LYS A  13       0.743  -0.947  -7.312  1.00  0.78           C
ATOM     78 O   LYS A  13       0.435   0.238  -7.416  1.00  0.78           O
ATOM     79 CB  LYS A  13       2.733  -1.657  -8.581  1.00  0.78           C
ATOM     80 CG  LYS A  13       2.854  -0.387  -9.438  1.00  0.78           C
ATOM     81 CD  LYS A  13       3.412  -0.675 -10.839  1.00  0.78           C
ATOM     82 CE  LYS A  13       2.499  -1.495 -11.753  1.00  0.78           C
ATOM     83 NZ  LYS A  13       1.281  -0.718 -12.058  1.00  0.78           N
ATOM     84 N   LYS A  14      -0.191  -1.910  -7.347  1.00  0.80           N
ATOM     85 CA  LYS A  14      -1.596  -1.608  -7.229  1.00  0.80           C
ATOM     86 C   LYS A  14      -1.995  -1.024  -5.890  1.00  0.80           C
ATOM     87 O   LYS A  14      -2.712  -0.035  -5.853  1.00  0.80           O
ATOM     88 CB  LYS A  14      -2.452  -2.840  -7.544  1.00  0.80           C
ATOM     89 CG  LYS A  14      -2.402  -3.216  -9.030  1.00  0.80           C
ATOM     90 CD  LYS A  14      -3.562  -4.125  -9.454  1.00  0.80           C
ATOM     91 CE  LYS A  14      -4.941  -3.456  -9.418  1.00  0.80           C
ATOM     92 NZ  LYS A  14      -4.985  -2.274 -10.304  1.00  0.80           N
ATOM     93 N   ALA A  15      -1.501  -1.565  -4.762  1.00  0.85           N
ATOM     94 CA  ALA A  15      -1.711  -0.982  -3.454  1.00  0.85           C
ATOM     95 C   ALA A  15      -1.161   0.439  -3.360  1.00  0.85           C
ATOM     96 O   ALA A  15      -1.798   1.310  -2.775  1.00  0.85           O
ATOM     97 CB  ALA A  15      -1.052  -1.848  -2.373  1.00  0.85           C
ATOM     98 N   ILE A  16       0.009   0.697  -3.993  1.00  0.78           N
ATOM     99 CA  ILE A  16       0.617   2.018  -4.133  1.00  0.78           C
ATOM    100 C   ILE A  16      -0.289   3.040  -4.788  1.00  0.78           C
ATOM    101 O   ILE A  16      -0.435   4.141  -4.267  1.00  0.78           O
ATOM    102 CB  ILE A  16       1.937   2.005  -4.903  1.00  0.78           C
ATOM    103 CG1 ILE A  16       3.040   1.193  -4.195  1.00  0.78           C
ATOM    104 CG2 ILE A  16       2.471   3.426  -5.185  1.00  0.78           C
ATOM    105 CD1 ILE A  16       3.381   1.661  -2.785  1.00  0.78           C
ATOM    106 N   ASP A  17      -0.954   2.705  -5.911  1.00  0.80           N
ATOM    107 CA  ASP A  17      -1.905   3.587  -6.559  1.00  0.80           C
ATOM    108 C   ASP A  17      -3.056   3.931  -5.614  1.00  0.80           C
ATOM    109 O   ASP A  17      -3.422   5.088  -5.425  1.00  0.80           O
ATOM    110 CB  ASP A  17      -2.384   2.874  -7.847  1.00  0.80           C
ATOM    111 CG  ASP A  17      -3.412   3.677  -8.627  1.00  0.80           C
ATOM    112 OD1 ASP A  17      -3.004   4.662  -9.288  1.00  0.80           O
ATOM    113 OD2 ASP A  17      -4.595   3.250  -8.612  1.00  0.80           O
ATOM    114 N   ILE A  18      -3.593   2.920  -4.913  1.00  0.76           N
ATOM    115 CA  ILE A  18      -4.713   3.111  -4.014  1.00  0.76           C
ATOM    116 C   ILE A  18      -4.382   3.955  -2.791  1.00  0.76           C
ATOM    117 O   ILE A  18      -5.128   4.874  -2.450  1.00  0.76           O
ATOM    118 CB  ILE A  18      -5.293   1.789  -3.556  1.00  0.76           C
ATOM    119 CG1 ILE A  18      -5.596   0.855  -4.746  1.00  0.76           C
ATOM    120 CG2 ILE A  18      -6.586   2.055  -2.759  1.00  0.76           C
ATOM    121 CD1 ILE A  18      -5.826  -0.591  -4.308  1.00  0.76           C
ATOM    122 N   ILE A  19      -3.248   3.708  -2.106  1.00  0.76           N
ATOM    123 CA  ILE A  19      -2.803   4.494  -0.953  1.00  0.76           C
ATOM    124 C   ILE A  19      -2.416   5.916  -1.317  1.00  0.76           C
ATOM    125 O   ILE A  19      -2.462   6.800  -0.458  1.00  0.76           O
ATOM    126 CB  ILE A  19      -1.625   3.890  -0.172  1.00  0.76           C
ATOM    127 CG1 ILE A  19      -0.357   3.752  -1.036  1.00  0.76           C
ATOM    128 CG2 ILE A  19      -2.026   2.528   0.412  1.00  0.76           C
ATOM    129 CD1 ILE A  19       0.854   3.169  -0.311  1.00  0.76           C
ATOM    130 N   ASP A  20      -1.988   6.134  -2.579  1.00  0.77           N
ATOM    131 CA  ASP A  20      -1.671   7.428  -3.139  1.00  0.77           C
ATOM    132 C   ASP A  20      -2.917   8.235  -3.477  1.00  0.77           C
ATOM    133 O   ASP A  20      -3.041   9.408  -3.120  1.00  0.77           O
ATOM    134 CB  ASP A  20      -0.819   7.207  -4.415  1.00  0.77           C
ATOM    135 CG  ASP A  20      -0.208   8.490  -4.929  1.00  0.77           C
ATOM    136 OD1 ASP A  20       0.542   9.137  -4.156  1.00  0.77           O
ATOM    137 OD2 ASP A  20      -0.314   8.768  -6.155  1.00  0.77           O
ATOM    138 N   ALA A  21      -3.879   7.605  -4.180  1.00  0.75           N
```

ATOM 139 CA ALA A 21 -5.051 8.269 -4.693 1.00 0.75 C
ATOM 140 C ALA A 21 -6.083 8.592 -3.640 1.00 0.75 C
ATOM 141 O ALA A 21 -6.717 9.647 -3.623 1.00 0.75 O
ATOM 142 CB ALA A 21 -5.705 7.364 -5.752 1.00 0.75 C
ATOM 143 N ALA A 22 -6.301 7.625 -2.744 1.00 0.77 N
ATOM 144 CA ALA A 22 -7.265 7.722 -1.699 1.00 0.77 C
ATOM 145 C ALA A 22 -6.736 8.481 -0.498 1.00 0.77 C
ATOM 146 O ALA A 22 -5.552 8.459 -0.168 1.00 0.77 O
ATOM 147 CB ALA A 22 -7.679 6.297 -1.330 1.00 0.77 C
ATOM 148 N SER A 23 -7.646 9.169 0.217 1.00 0.68 N
ATOM 149 CA SER A 23 -7.284 10.123 1.253 1.00 0.68 C
ATOM 150 C SER A 23 -7.472 9.526 2.628 1.00 0.68 C
ATOM 151 O SER A 23 -6.520 9.203 3.343 1.00 0.68 O
ATOM 152 CB SER A 23 -8.142 11.407 1.142 1.00 0.68 C
ATOM 153 OG SER A 23 -7.820 12.074 -0.075 1.00 0.68 O
ATOM 154 N THR A 24 -8.745 9.369 3.031 1.00 0.71 N
ATOM 155 CA THR A 24 -9.197 8.800 4.292 1.00 0.71 C
ATOM 156 C THR A 24 -8.743 7.376 4.471 1.00 0.71 C
ATOM 157 O THR A 24 -8.958 6.547 3.589 1.00 0.71 O
ATOM 158 CB THR A 24 -10.714 8.815 4.384 1.00 0.71 C
ATOM 159 OG1 THR A 24 -11.170 10.156 4.309 1.00 0.71
O
ATOM 160 CG2 THR A 24 -11.261 8.208 5.684 1.00 0.71
C
ATOM 161 N ILE A 25 -8.147 7.026 5.634 1.00 0.67 N
ATOM 162 CA ILE A 25 -7.600 5.698 5.890 1.00 0.67 C
ATOM 163 C ILE A 25 -8.612 4.585 5.661 1.00 0.67 C
ATOM 164 O ILE A 25 -8.351 3.647 4.917 1.00 0.67 O
ATOM 165 CB ILE A 25 -7.039 5.627 7.308 1.00 0.67 C
ATOM 166 CG1 ILE A 25 -5.755 6.478 7.430 1.00 0.67 C
ATOM 167 CG2 ILE A 25 -6.777 4.172 7.756 1.00 0.67 C
ATOM 168 CD1 ILE A 25 -5.305 6.663 8.884 1.00 0.67 C
ATOM 169 N ALA A 26 -9.829 4.716 6.222 1.00 0.76 N
ATOM 170 CA ALA A 26 -10.908 3.762 6.075 1.00 0.76 C
ATOM 171 C ALA A 26 -11.319 3.524 4.631 1.00 0.76 C
ATOM 172 O ALA A 26 -11.567 2.399 4.210 1.00 0.76 O
ATOM 173 CB ALA A 26 -12.116 4.238 6.901 1.00 0.76 C
ATOM 174 N SER A 27 -11.342 4.605 3.826 1.00 0.76 N
ATOM 175 CA SER A 27 -11.611 4.548 2.401 1.00 0.76 C
ATOM 176 C SER A 27 -10.554 3.744 1.669 1.00 0.76 C
ATOM 177 O SER A 27 -10.863 2.896 0.841 1.00 0.76 O
ATOM 178 CB SER A 27 -11.710 5.959 1.773 1.00 0.76 C
ATOM 179 OG SER A 27 -12.727 6.732 2.423 1.00 0.76 O
ATOM 180 N ILE A 28 -9.267 3.968 2.013 1.00 0.71 N
ATOM 181 CA ILE A 28 -8.122 3.252 1.457 1.00 0.71 C
ATOM 182 C ILE A 28 -8.142 1.772 1.748 1.00 0.71 C
ATOM 183 O ILE A 28 -7.953 0.950 0.847 1.00 0.71 O
ATOM 184 CB ILE A 28 -6.790 3.772 1.990 1.00 0.71 C
ATOM 185 CG1 ILE A 28 -6.633 5.263 1.699 1.00 0.71 C
ATOM 186 CG2 ILE A 28 -5.661 3.069 1.241 1.00 0.71 C
ATOM 187 CD1 ILE A 28 -5.447 6.043 2.270 1.00 0.71 C
ATOM 188 N ILE A 29 -8.403 1.399 3.014 1.00 0.70 N
ATOM 189 CA ILE A 29 -8.423 0.022 3.492 1.00 0.70 C
ATOM 190 C ILE A 29 -9.438 -0.784 2.722 1.00 0.70 C
ATOM 191 O ILE A 29 -9.157 -1.878 2.230 1.00 0.70 O
ATOM 192 CB ILE A 29 -8.811 -0.052 4.967 1.00 0.70 C
ATOM 193 CG1 ILE A 29 -7.784 0.644 5.875 1.00 0.70 C
ATOM 194 CG2 ILE A 29 -8.974 -1.520 5.426 1.00 0.70 C
ATOM 195 CD1 ILE A 29 -8.326 0.892 7.284 1.00 0.70 C
ATOM 196 N SER A 30 -10.645 -0.205 2.555 1.00 0.77 N
ATOM 197 CA SER A 30 -11.705 -0.831 1.794 1.00 0.77 C
ATOM 198 C SER A 30 -11.302 -1.068 0.357 1.00 0.77 C
ATOM 199 O SER A 30 -11.434 -2.174 -0.159 1.00 0.77 O
ATOM 200 CB SER A 30 -13.020 -0.015 1.837 1.00 0.77 C
ATOM 201 OG SER A 30 -13.552 0.008 3.164 1.00 0.77 O
ATOM 202 N LEU A 31 -10.727 -0.061 -0.321 1.00 0.74 N
ATOM 203 CA LEU A 31 -10.292 -0.203 -1.695 1.00 0.74 C
ATOM 204 C LEU A 31 -9.155 -1.178 -1.898 1.00 0.74 C
ATOM 205 O LEU A 31 -9.181 -1.991 -2.819 1.00 0.74 O
ATOM 206 CB LEU A 31 -9.880 1.150 -2.290 1.00 0.74 C
ATOM 207 CG LEU A 31 -10.993 2.205 -2.349 1.00 0.74 C
ATOM 208 CD1 LEU A 31 -10.411 3.532 -2.857 1.00 0.74
C
ATOM 209 CD2 LEU A 31 -12.206 1.755 -3.176 1.00 0.74
C
ATOM 210 N ILE A 32 -8.131 -1.147 -1.029 1.00 0.73 N
ATOM 211 CA ILE A 32 -7.032 -2.090 -1.094 1.00 0.73 C
ATOM 212 C ILE A 32 -7.465 -3.516 -0.876 1.00 0.73 C
ATOM 213 O ILE A 32 -7.085 -4.407 -1.630 1.00 0.73 O
ATOM 214 CB ILE A 32 -5.950 -1.742 -0.111 1.00 0.73 C
ATOM 215 CG1 ILE A 32 -5.300 -0.429 -0.534 1.00 0.73 C
ATOM 216 CG2 ILE A 32 -4.875 -2.832 -0.092 1.00 0.73 C
ATOM 217 CD1 ILE A 32 -4.321 0.075 0.508 1.00 0.73 C
ATOM 218 N GLY A 33 -8.314 -3.753 0.141 1.00 0.76 N
ATOM 219 CA GLY A 33 -8.805 -5.087 0.445 1.00 0.76 C
ATOM 220 C GLY A 33 -9.653 -5.700 -0.641 1.00 0.76 C
ATOM 221 O GLY A 33 -9.634 -6.914 -0.844 1.00 0.76 O
ATOM 222 N ILE A 34 -10.386 -4.849 -1.392 1.00 0.68 N
ATOM 223 CA ILE A 34 -11.117 -5.195 -2.608 1.00 0.68 C
ATOM 224 C ILE A 34 -10.182 -5.638 -3.721 1.00 0.68 C
ATOM 225 O ILE A 34 -10.451 -6.608 -4.429 1.00 0.68 O
ATOM 226 CB ILE A 34 -12.022 -4.043 -3.068 1.00 0.68 C
ATOM 227 CG1 ILE A 34 -13.192 -3.848 -2.077 1.00 0.68 C
ATOM 228 CG2 ILE A 34 -12.578 -4.262 -4.495 1.00 0.68 C
ATOM 229 CD1 ILE A 34 -13.941 -2.524 -2.278 1.00 0.68 C
ATOM 230 N VAL A 35 -9.050 -4.931 -3.903 1.00 0.76 N
ATOM 231 CA VAL A 35 -8.087 -5.215 -4.951 1.00 0.76 C
ATOM 232 C VAL A 35 -7.189 -6.404 -4.670 1.00 0.76 C
ATOM 233 O VAL A 35 -6.974 -7.248 -5.538 1.00 0.76 O
ATOM 234 CB VAL A 35 -7.229 -3.991 -5.231 1.00 0.76 C
ATOM 235 CG1 VAL A 35 -6.064 -4.305 -6.192 1.00 0.76
C
ATOM 236 CG2 VAL A 35 -8.141 -2.903 -5.834 1.00 0.76
C
ATOM 237 N THR A 36 -6.610 -6.504 -3.458 1.00 0.76 N
ATOM 238 CA THR A 36 -5.709 -7.604 -3.137 1.00 0.76 C
ATOM 239 C THR A 36 -6.447 -8.871 -2.764 1.00 0.76 C
ATOM 240 O THR A 36 -5.891 -9.966 -2.839 1.00 0.76 O
ATOM 241 CB THR A 36 -4.714 -7.301 -2.017 1.00 0.76 C
ATOM 242 OG1 THR A 36 -5.352 -6.916 -0.805 1.00 0.76
O
ATOM 243 CG2 THR A 36 -3.802 -6.140 -2.435 1.00 0.76
C
ATOM 244 N GLY A 37 -7.733 -8.753 -2.366 1.00 0.75 N
ATOM 245 CA GLY A 37 -8.574 -9.882 -1.994 1.00 0.75 C
ATOM 246 C GLY A 37 -8.211 -10.452 -0.653 1.00 0.75 C
ATOM 247 O GLY A 37 -8.321 -11.655 -0.409 1.00 0.75 O
ATOM 248 N ALA A 38 -7.755 -9.585 0.261 1.00 0.68 N
ATOM 249 CA ALA A 38 -7.313 -9.989 1.563 1.00 0.68 C
ATOM 250 C ALA A 38 -7.343 -8.789 2.490 1.00 0.68 C
ATOM 251 O ALA A 38 -7.177 -7.645 2.078 1.00 0.68 O
ATOM 252 CB ALA A 38 -5.884 -10.576 1.487 1.00 0.68 C
ATOM 253 N GLY A 39 -7.510 -9.026 3.809 1.00 0.64 N
ATOM 254 CA GLY A 39 -7.517 -7.978 4.833 1.00 0.64 C
ATOM 255 C GLY A 39 -6.144 -7.710 5.389 1.00 0.64 C
ATOM 256 O GLY A 39 -5.995 -7.268 6.524 1.00 0.64 O
ATOM 257 N ALA A 40 -5.101 -8.022 4.594 1.00 0.67 N
ATOM 258 CA ALA A 40 -3.700 -7.870 4.928 1.00 0.67 C
ATOM 259 C ALA A 40 -3.309 -6.418 5.111 1.00 0.67 C
ATOM 260 O ALA A 40 -2.608 -6.039 6.048 1.00 0.67 O
ATOM 261 CB ALA A 40 -2.835 -8.493 3.813 1.00 0.67 C
ATOM 262 N ILE A 41 -3.806 -5.549 4.217 1.00 0.69 N
ATOM 263 CA ILE A 41 -3.562 -4.135 4.307 1.00 0.69 C
ATOM 264 C ILE A 41 -4.631 -3.534 5.189 1.00 0.69 C
ATOM 265 O ILE A 41 -5.737 -3.186 4.776 1.00 0.69 O
ATOM 266 CB ILE A 41 -3.499 -3.419 2.974 1.00 0.69 C
ATOM 267 CG1 ILE A 41 -2.384 -3.966 2.048 1.00 0.69 C
ATOM 268 CG2 ILE A 41 -3.261 -1.915 3.246 1.00 0.69 C

ATOM 269 CD1 ILE A 41 -2.711 -5.240 1.253 1.00 0.69 C
ATOM 270 N SER A 42 -4.284 -3.420 6.470 1.00 0.72 N
ATOM 271 CA SER A 42 -5.088 -2.831 7.507 1.00 0.72 C
ATOM 272 C SER A 42 -4.767 -1.358 7.631 1.00 0.72 C
ATOM 273 O SER A 42 -3.936 -0.821 6.902 1.00 0.72 O
ATOM 274 CB SER A 42 -4.838 -3.556 8.858 1.00 0.72 C
ATOM 275 OG SER A 42 -3.547 -3.271 9.416 1.00 0.72 O
ATOM 276 N TYR A 43 -5.387 -0.656 8.602 1.00 0.66 N
ATOM 277 CA TYR A 43 -5.124 0.746 8.887 1.00 0.66 C
ATOM 278 C TYR A 43 -3.650 1.052 9.141 1.00 0.66 C
ATOM 279 O TYR A 43 -3.125 2.068 8.693 1.00 0.66 O
ATOM 280 CB TYR A 43 -6.003 1.243 10.084 1.00 0.66 C
ATOM 281 CG TYR A 43 -5.550 0.729 11.430 1.00 0.66 C
ATOM 282 CD1 TYR A 43 -4.620 1.471 12.176 1.00 0.66
C
ATOM 283 CD2 TYR A 43 -5.967 -0.514 11.931 1.00 0.66
C
ATOM 284 CE1 TYR A 43 -4.090 0.964 13.367 1.00 0.66 C
ATOM 285 CE2 TYR A 43 -5.453 -1.004 13.141 1.00 0.66
C
ATOM 286 CZ TYR A 43 -4.510 -0.267 13.858 1.00 0.66 C
ATOM 287 OH TYR A 43 -3.960 -0.760 15.059 1.00 0.66
O
ATOM 288 N ALA A 44 -2.966 0.131 9.857 1.00 0.77 N
ATOM 289 CA ALA A 44 -1.567 0.185 10.202 1.00 0.77 C
ATOM 290 C ALA A 44 -0.704 0.131 8.960 1.00 0.77 C
ATOM 291 O ALA A 44 0.225 0.910 8.785 1.00 0.77 O
ATOM 292 CB ALA A 44 -1.232 -0.989 11.153 1.00 0.77 C
ATOM 293 N ILE A 45 -1.036 -0.775 8.024 1.00 0.73 N
ATOM 294 CA ILE A 45 -0.350 -0.875 6.752 1.00 0.73 C
ATOM 295 C ILE A 45 -0.603 0.333 5.872 1.00 0.73 C
ATOM 296 O ILE A 45 0.333 0.863 5.280 1.00 0.73 O
ATOM 297 CB ILE A 45 -0.705 -2.147 6.009 1.00 0.73 C
ATOM 298 CG1 ILE A 45 -0.464 -3.417 6.859 1.00 0.73 C
ATOM 299 CG2 ILE A 45 0.059 -2.221 4.666 1.00 0.73 C
ATOM 300 CD1 ILE A 45 0.985 -3.621 7.310 1.00 0.73 C
ATOM 301 N VAL A 46 -1.856 0.829 5.782 1.00 0.80 N
ATOM 302 CA VAL A 46 -2.189 2.035 5.027 1.00 0.80 C
ATOM 303 C VAL A 46 -1.482 3.268 5.533 1.00 0.80 C
ATOM 304 O VAL A 46 -0.890 4.022 4.755 1.00 0.80 O
ATOM 305 CB VAL A 46 -3.677 2.357 5.098 1.00 0.80 C
ATOM 306 CG1 VAL A 46 -4.024 3.701 4.422 1.00 0.80 C
ATOM 307 CG2 VAL A 46 -4.472 1.253 4.399 1.00 0.80 C
ATOM 308 N ALA A 47 -1.521 3.501 6.861 1.00 0.83 N
ATOM 309 CA ALA A 47 -0.886 4.632 7.485 1.00 0.83 C
ATOM 310 C ALA A 47 0.616 4.584 7.287 1.00 0.83 C
ATOM 311 O ALA A 47 1.219 5.532 6.810 1.00 0.83 O
ATOM 312 CB ALA A 47 -1.229 4.683 8.989 1.00 0.83 C
ATOM 313 N THR A 48 1.239 3.419 7.568 1.00 0.83 N
ATOM 314 CA THR A 48 2.664 3.230 7.342 1.00 0.83 C
ATOM 315 C THR A 48 3.035 3.368 5.898 1.00 0.83 C
ATOM 316 O THR A 48 3.935 4.132 5.568 1.00 0.83 O
ATOM 317 CB THR A 48 3.177 1.900 7.858 1.00 0.83 C
ATOM 318 OG1 THR A 48 2.913 1.792 9.248 1.00 0.83 O
ATOM 319 CG2 THR A 48 4.701 1.757 7.724 1.00 0.83 C
ATOM 320 N ALA A 49 2.320 2.732 4.958 1.00 0.86 N
ATOM 321 CA ALA A 49 2.645 2.811 3.559 1.00 0.86 C
ATOM 322 C ALA A 49 2.563 4.220 2.991 1.00 0.86 C
ATOM 323 O ALA A 49 3.486 4.669 2.318 1.00 0.86 O
ATOM 324 CB ALA A 49 1.741 1.845 2.784 1.00 0.86 C
ATOM 325 N LYS A 50 1.504 4.996 3.311 1.00 0.79 N
ATOM 326 CA LYS A 50 1.417 6.374 2.867 1.00 0.79 C
ATOM 327 C LYS A 50 2.469 7.287 3.492 1.00 0.79 C
ATOM 328 O LYS A 50 2.895 8.283 2.908 1.00 0.79 O
ATOM 329 CB LYS A 50 0.023 7.009 3.031 1.00 0.79 C
ATOM 330 CG LYS A 50 -0.390 7.329 4.472 1.00 0.79 C
ATOM 331 CD LYS A 50 -1.555 8.320 4.528 1.00 0.79 C
ATOM 332 CE LYS A 50 -2.837 7.782 3.895 1.00 0.79 C
ATOM 333 NZ LYS A 50 -3.829 8.867 3.783 1.00 0.79 N
ATOM 334 N THR A 51 2.920 6.984 4.731 1.00 0.84 N
ATOM 335 CA THR A 51 4.027 7.713 5.344 1.00 0.84 C
ATOM 336 C THR A 51 5.341 7.396 4.684 1.00 0.84 C
ATOM 337 O THR A 51 6.162 8.299 4.522 1.00 0.84 O
ATOM 338 CB THR A 51 4.207 7.641 6.859 1.00 0.84 C
ATOM 339 OG1 THR A 51 4.596 6.371 7.369 1.00 0.84 O
ATOM 340 CG2 THR A 51 2.934 8.101 7.563 1.00 0.84 C
ATOM 341 N MET A 52 5.573 6.126 4.297 1.00 0.80 N
ATOM 342 CA MET A 52 6.719 5.654 3.539 1.00 0.80 C
ATOM 343 C MET A 52 6.806 6.244 2.146 1.00 0.80 C
ATOM 344 O MET A 52 7.881 6.657 1.715 1.00 0.80 O
ATOM 345 CB MET A 52 6.779 4.112 3.415 1.00 0.80 C
ATOM 346 CG MET A 52 6.886 3.294 4.723 1.00 0.80 C
ATOM 347 SD MET A 52 8.332 3.563 5.779 1.00 0.80 S
ATOM 348 CE MET A 52 7.701 4.902 6.842 1.00 0.80 C
ATOM 349 N ILE A 53 5.679 6.353 1.411 1.00 0.77 N
ATOM 350 CA ILE A 53 5.686 6.982 0.097 1.00 0.77 C
ATOM 351 C ILE A 53 5.927 8.476 0.148 1.00 0.77 C
ATOM 352 O ILE A 53 6.391 9.076 -0.816 1.00 0.77 O
ATOM 353 CB ILE A 53 4.459 6.705 -0.771 1.00 0.77 C
ATOM 354 CG1 ILE A 53 3.198 7.417 -0.251 1.00 0.77 C
ATOM 355 CG2 ILE A 53 4.275 5.185 -0.858 1.00 0.77 C
ATOM 356 CD1 ILE A 53 1.925 7.196 -1.071 1.00 0.77 C
ATOM 357 N LYS A 54 5.618 9.112 1.292 1.00 0.77 N
ATOM 358 CA LYS A 54 5.920 10.504 1.520 1.00 0.77 C
ATOM 359 C LYS A 54 7.398 10.813 1.712 1.00 0.77 C
ATOM 360 O LYS A 54 7.902 11.798 1.174 1.00 0.77 O
ATOM 361 CB LYS A 54 5.126 10.999 2.753 1.00 0.77 C
ATOM 362 CG LYS A 54 5.310 12.486 3.097 1.00 0.77 C
ATOM 363 CD LYS A 54 4.865 13.440 1.979 1.00 0.77 C
ATOM 364 CE LYS A 54 3.373 13.413 1.657 1.00 0.77 C
ATOM 365 NZ LYS A 54 2.620 13.945 2.810 1.00 0.77 N
ATOM 366 N LYS A 55 8.116 10.001 2.515 1.00 0.78 N
ATOM 367 CA LYS A 55 9.533 10.205 2.767 1.00 0.78 C
ATOM 368 C LYS A 55 10.445 9.536 1.759 1.00 0.78 C
ATOM 369 O LYS A 55 11.368 10.156 1.238 1.00 0.78 O
ATOM 370 CB LYS A 55 9.956 9.781 4.203 1.00 0.78 C
ATOM 371 CG LYS A 55 9.365 8.460 4.709 1.00 0.78 C
ATOM 372 CD LYS A 55 9.700 8.134 6.173 1.00 0.78 C
ATOM 373 CE LYS A 55 9.117 9.092 7.210 1.00 0.78 C
ATOM 374 NZ LYS A 55 7.640 9.059 7.156 1.00 0.78 N
ATOM 375 N TYR A 56 10.223 8.245 1.469 1.00 0.78 N
ATOM 376 CA TYR A 56 11.145 7.463 0.677 1.00 0.78 C
ATOM 377 C TYR A 56 10.659 7.308 -0.749 1.00 0.78 C
ATOM 378 O TYR A 56 11.431 7.299 -1.707 1.00 0.78 O
ATOM 379 CB TYR A 56 11.264 6.050 1.289 1.00 0.78 C
ATOM 380 CG TYR A 56 11.638 6.043 2.744 1.00 0.78 C
ATOM 381 CD1 TYR A 56 12.679 6.832 3.247 1.00 0.78
C
ATOM 382 CD2 TYR A 56 10.952 5.209 3.635 1.00 0.78
C
ATOM 383 CE1 TYR A 56 12.992 6.822 4.612 1.00 0.78 C
ATOM 384 CE2 TYR A 56 11.327 5.124 4.977 1.00 0.78 C
ATOM 385 CZ TYR A 56 12.316 5.963 5.477 1.00 0.78 C
ATOM 386 OH TYR A 56 12.560 5.963 6.858 1.00 0.78 O
ATOM 387 N GLY A 57 9.329 7.203 -0.917 1.00 0.89 N
ATOM 388 CA GLY A 57 8.694 7.192 -2.221 1.00 0.89 C
ATOM 389 C GLY A 57 7.858 5.975 -2.481 1.00 0.89 C
ATOM 390 O GLY A 57 7.917 4.953 -1.800 1.00 0.89 O
ATOM 391 N LYS A 58 7.031 6.060 -3.535 1.00 0.76 N
ATOM 392 CA LYS A 58 6.108 5.020 -3.949 1.00 0.76 C
ATOM 393 C LYS A 58 6.746 3.673 -4.216 1.00 0.76 C
ATOM 394 O LYS A 58 6.278 2.622 -3.790 1.00 0.76 O
ATOM 395 CB LYS A 58 5.419 5.499 -5.227 1.00 0.76 C
ATOM 396 CG LYS A 58 4.438 6.633 -4.962 1.00 0.76 C
ATOM 397 CD LYS A 58 3.808 7.083 -6.281 1.00 0.76 C
ATOM 398 CE LYS A 58 2.815 8.200 -6.010 1.00 0.76 C
ATOM 399 NZ LYS A 58 2.040 8.615 -7.193 1.00 0.76 N
ATOM 400 N LYS A 59 7.891 3.698 -4.905 1.00 0.74 N

```
ATOM    401  CA  LYS A  59      8.678   2.526  -5.201  1.00  0.74           C
ATOM    402  C   LYS A  59     9.297   1.852  -3.979  1.00  0.74           C
ATOM    403  O   LYS A  59     9.439   0.631  -3.933  1.00  0.74           O
ATOM    404  CB  LYS A  59      9.763   2.913  -6.218  1.00  0.74           C
ATOM    405  CG  LYS A  59     10.534   1.711  -6.775  1.00  0.74           C
ATOM    406  CD  LYS A  59     11.528   2.100  -7.877  1.00  0.74           C
ATOM    407  CE  LYS A  59     12.664   2.993  -7.373  1.00  0.74           C
ATOM    408  NZ  LYS A  59     13.621   3.281  -8.464  1.00  0.74           N
ATOM    409  N   TYR A  60     9.672   2.640  -2.943  1.00  0.73           N
ATOM    410  CA  TYR A  60     10.147   2.103  -1.681  1.00  0.73           C
ATOM    411  C   TYR A  60     9.045   1.316  -1.008  1.00  0.73           C
ATOM    412  O   TYR A  60     9.221   0.177  -0.583  1.00  0.73           O
ATOM    413  CB  TYR A  60     10.582   3.257  -0.735  1.00  0.73           C
ATOM    414  CG  TYR A  60     10.969   2.759   0.636  1.00  0.73           C
ATOM    415  CD1 TYR A  60      9.993   2.560   1.627  1.00  0.73           C
ATOM    416  CD2 TYR A  60     12.291   2.418   0.922  1.00  0.73
C
ATOM    417  CE1 TYR A  60     10.326   1.985   2.857  1.00  0.73           C
ATOM    418  CE2 TYR A  60     12.634   1.883   2.171  1.00  0.73           C
ATOM    419  CZ  TYR A  60     11.650   1.665   3.139  1.00  0.73           C
ATOM    420  OH  TYR A  60     11.978   1.136   4.399  1.00  0.73           O
ATOM    421  N   ALA A  61     7.845   1.928  -0.919  1.00  0.83           N
ATOM    422  CA  ALA A  61      6.727   1.299  -0.271  1.00  0.83           C
ATOM    423  C   ALA A  61     6.279   0.047  -1.009  1.00  0.83           C
ATOM    424  O   ALA A  61     5.865  -0.907  -0.399  1.00  0.83           O
ATOM    425  CB  ALA A  61      5.554   2.258  -0.038  1.00  0.83           C
ATOM    426  N   ALA A  62     6.420   0.008  -2.359  1.00  0.81           N
ATOM    427  CA  ALA A  62      6.133  -1.176  -3.138  1.00  0.81           C
ATOM    428  C   ALA A  62     6.975  -2.370  -2.670  1.00  0.81           C
ATOM    429  O   ALA A  62     6.441  -3.444  -2.388  1.00  0.81           O
ATOM    430  CB  ALA A  62      6.379  -0.867  -4.633  1.00  0.81           C
ATOM    431  N   ALA A  63     8.294  -2.146  -2.479  1.00  0.74           N
ATOM    432  CA  ALA A  63      9.259  -3.120  -1.984  1.00  0.74           C
ATOM    433  C   ALA A  63     9.173  -3.550  -0.517  1.00  0.74           C
ATOM    434  O   ALA A  63     9.517  -4.701  -0.227  1.00  0.74           O
ATOM    435  CB  ALA A  63     10.698  -2.625  -2.198  1.00  0.74           C
ATOM    436  N   TRP A  64     8.777  -2.622   0.402  1.00  0.68           N
ATOM    437  CA  TRP A  64      8.705  -2.737   1.859  1.00  0.68           C
ATOM    438  C   TRP A  64     7.778  -3.883   2.319  1.00  0.68           C
ATOM    439  O   TRP A  64     7.558  -4.851   1.569  1.00  0.68           O
ATOM    440  CB  TRP A  64      8.153  -1.391   2.462  1.00  0.68           C
ATOM    441  CG  TRP A  64      7.682  -1.295   3.939  1.00  0.68           C
ATOM    442  CD1 TRP A  64      8.478  -1.452   5.032  1.00  0.68           C
ATOM    443  CD2 TRP A  64      6.315  -1.328   4.454  1.00  0.68           C
ATOM    444  NE1 TRP A  64      7.729  -1.553   6.184  1.00  0.68           N
ATOM    445  CE2 TRP A  64      6.402  -1.510   5.845  1.00  0.68           C
ATOM    446  CE3 TRP A  64      5.065  -1.252   3.833  1.00  0.68           C
ATOM    447  CZ2 TRP A  64      5.263  -1.632   6.634  1.00  0.68           C
ATOM    448  CZ3 TRP A  64      3.917  -1.357   4.635  1.00  0.68           C
ATOM    449  CH2 TRP A  64      4.013  -1.554   6.012  1.00  0.68           C
ATOM    450  OXT TRP A  64      7.105  -3.771   3.364  1.00  0.68           O
TER     451      TRP A  64
END
```

B)

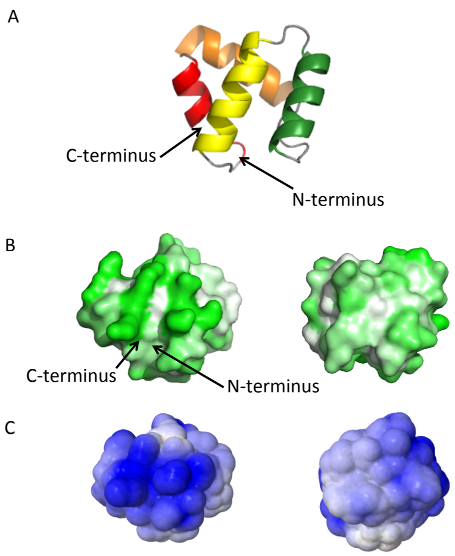

Figure S3- Structural models of amylocyclicin. A) Ribbon diagram depicting the saposin-like fold. The C- and N- termini are explicitly shown. Hydrophobic surface (B) and electrostatic potential surface (C) maps of amylocyclicin. Orientation peptide backbone in surface filling diagrams, B and C, correlate to one another.

4. Leucocyclicin

A) PDB file for model of leucocyclicin. Structure modeled after NKR-5-3B using SWISS-MODEL[84]

```
TITLE     SWISS-MODEL SERVER (https://swissmodel.expasy.org)
TITLE    2 Untitled Project
EXPDTA    THEORETICAL MODEL (SWISS-MODEL SERVER)
```

AUTHOR SWISS-MODEL SERVER (SEE REFERENCE IN JRNL
Records)
REVDAT 1 11-AUG-16 1MOD 1 23:52
JRNL AUTH
M.BIASINI,S.BIENERT,A.WATERHOUSE,K.ARNOLD,G.STUDER,
JRNL AUTH 2 T.SCHMIDT,F.KIEFER,T.GALLO
CASSARINO,M.BERTONI,L.BORDOLI,
JRNL AUTH 3 T.SCHWEDE
JRNL TITL SWISS-MODEL: MODELLING PROTEIN TERTIARY
AND QUATERNARY
JRNL TITL 2 STRUCTURE USING EVOLUTIONARY
INFORMATION
JRNL REF NUCLEIC.ACIDS.RES 2014
JRNL REFN ISSN 0305-1048
JRNL PMID 24782522
JRNL DOI 10.1093/nar/gku340
REMARK 3
REMARK 3 MODEL INFORMATION
REMARK 3 ENGIN PROMOD3
REMARK 3 VERSN 1.0.0
REMARK 3 OSTAT MONOMER
REMARK 3 OSRSN PREDICTION
REMARK 3 GMQE 0.66
REMARK 3 QMN4 -1.92
REMARK 3
REMARK 3 TEMPLATE 1
REMARK 3 PDBID 2mp8
REMARK 3 CHAIN A
REMARK 3 MMCIF A
REMARK 3 PDBV 2016-07-29
REMARK 3 SMTLE 2mp8.1.A
REMARK 3 SMTLV 2016-08-03
REMARK 3 MTHD SOLUTION NMR 0.00 A
REMARK 3 FOUND HHblits
REMARK 3 GMQE 0.66
REMARK 3 SIM 0.36
REMARK 3 SID 31.67
REMARK 3 OSTAT monomer
ATOM 1 N VAL A 4 5.370 -5.541 -0.615 1.00 0.72 N
ATOM 2 CA VAL A 4 5.883 -6.910 -0.994 1.00 0.72 C
ATOM 3 C VAL A 4 6.449 -7.673 0.163 1.00 0.72 C
ATOM 4 O VAL A 4 5.908 -8.714 0.519 1.00 0.72 O
ATOM 5 CB VAL A 4 6.841 -6.860 -2.184 1.00 0.72 C
ATOM 6 CG1 VAL A 4 7.445 -8.224 -2.592 1.00 0.72 C
ATOM 7 CG2 VAL A 4 6.030 -6.366 -3.384 1.00 0.72 C
ATOM 8 N ASN A 5 7.514 -7.163 0.800 1.00 0.70 N
ATOM 9 CA ASN A 5 8.191 -7.839 1.891 1.00 0.70 C
ATOM 10 C ASN A 5 7.354 -8.123 3.135 1.00 0.70 C
ATOM 11 O ASN A 5 7.357 -9.241 3.637 1.00 0.70 O
ATOM 12 CB ASN A 5 9.401 -6.995 2.343 1.00 0.70 C
ATOM 13 CG ASN A 5 10.461 -6.974 1.250 1.00 0.70 C
ATOM 14 OD1 ASN A 5 10.573 -7.870 0.413 1.00 0.70 O
ATOM 15 ND2 ASN A 5 11.301 -5.913 1.258 1.00 0.70 N
ATOM 16 N GLN A 6 6.611 -7.141 3.671 1.00 0.75 N
ATOM 17 CA GLN A 6 5.860 -7.321 4.900 1.00 0.75 C
ATOM 18 C GLN A 6 4.523 -8.021 4.732 1.00 0.75 C
ATOM 19 O GLN A 6 4.044 -8.711 5.627 1.00 0.75 O
ATOM 20 CB GLN A 6 5.616 -5.947 5.560 1.00 0.75 C
ATOM 21 CG GLN A 6 6.912 -5.224 5.994 1.00 0.75 C
ATOM 22 CD GLN A 6 7.666 -6.028 7.049 1.00 0.75 C
ATOM 23 OE1 GLN A 6 7.120 -6.429 8.076 1.00 0.75 O
ATOM 24 NE2 GLN A 6 8.976 -6.271 6.809 1.00 0.75 N
ATOM 25 N LEU A 7 3.878 -7.859 3.564 1.00 0.72 N
ATOM 26 CA LEU A 7 2.585 -8.463 3.302 1.00 0.72 C
ATOM 27 C LEU A 7 2.708 -9.826 2.631 1.00 0.72 C
ATOM 28 O LEU A 7 1.779 -10.627 2.660 1.00 0.72 O
ATOM 29 CB LEU A 7 1.756 -7.553 2.356 1.00 0.72 C
ATOM 30 CG LEU A 7 1.557 -6.099 2.832 1.00 0.72 C
ATOM 31 CD1 LEU A 7 0.942 -5.250 1.707 1.00 0.72 C
ATOM 32 CD2 LEU A 7 0.706 -6.024 4.108 1.00 0.72 C
ATOM 33 N GLY A 8 3.863 -10.123 1.994 1.00 0.84 N
ATOM 34 CA GLY A 8 4.078 -11.358 1.238 1.00 0.84 C
ATOM 35 C GLY A 8 3.291 -11.435 -0.053 1.00 0.84 C
ATOM 36 O GLY A 8 2.777 -12.482 -0.433 1.00 0.84 O
ATOM 37 N ILE A 9 3.178 -10.295 -0.762 1.00 0.74 N
ATOM 38 CA ILE A 9 2.358 -10.123 -1.953 1.00 0.74 C
ATOM 39 C ILE A 9 3.261 -9.955 -3.155 1.00 0.74 C
ATOM 40 O ILE A 9 4.470 -9.792 -3.030 1.00 0.74 O
ATOM 41 CB ILE A 9 1.389 -8.937 -1.886 1.00 0.74 C
ATOM 42 CG1 ILE A 9 2.096 -7.573 -1.668 1.00 0.74 C
ATOM 43 CG2 ILE A 9 0.341 -9.282 -0.809 1.00 0.74 C
ATOM 44 CD1 ILE A 9 1.145 -6.372 -1.740 1.00 0.74 C
ATOM 45 N SER A 10 2.716 -9.983 -4.385 1.00 0.84 N
ATOM 46 CA SER A 10 3.498 -9.716 -5.582 1.00 0.84 C
ATOM 47 C SER A 10 3.818 -8.237 -5.768 1.00 0.84 C
ATOM 48 O SER A 10 3.170 -7.356 -5.205 1.00 0.84 O
ATOM 49 CB SER A 10 2.815 -10.254 -6.872 1.00 0.84 C
ATOM 50 OG SER A 10 1.608 -9.550 -7.183 1.00 0.84 O
ATOM 51 N LYS A 11 4.836 -7.912 -6.598 1.00 0.75 N
ATOM 52 CA LYS A 11 5.141 -6.542 -6.998 1.00 0.75 C
ATOM 53 C LYS A 11 4.009 -5.882 -7.773 1.00 0.75 C
ATOM 54 O LYS A 11 3.780 -4.682 -7.657 1.00 0.75 O
ATOM 55 CB LYS A 11 6.451 -6.462 -7.812 1.00 0.75 C
ATOM 56 CG LYS A 11 7.688 -6.849 -6.989 1.00 0.75 C
ATOM 57 CD LYS A 11 8.973 -6.781 -7.829 1.00 0.75 C
ATOM 58 CE LYS A 11 10.235 -7.150 -7.042 1.00 0.75 C
ATOM 59 NZ LYS A 11 11.427 -7.101 -7.920 1.00 0.75 N
ATOM 60 N SER A 12 3.258 -6.688 -8.555 1.00 0.80 N
ATOM 61 CA SER A 12 2.033 -6.277 -9.237 1.00 0.80 C
ATOM 62 C SER A 12 0.972 -5.789 -8.262 1.00 0.80 C
ATOM 63 O SER A 12 0.479 -4.668 -8.358 1.00 0.80 O
ATOM 64 CB SER A 12 1.435 -7.471 -10.037 1.00 0.80 C
ATOM 65 OG SER A 12 0.272 -7.104 -10.780 1.00 0.80 O
ATOM 66 N LEU A 13 0.665 -6.598 -7.223 1.00 0.74 N
ATOM 67 CA LEU A 13 -0.262 -6.215 -6.176 1.00 0.74 C
ATOM 68 C LEU A 13 0.219 -5.013 -5.384 1.00 0.74 C
ATOM 69 O LEU A 13 -0.549 -4.105 -5.102 1.00 0.74 O
ATOM 70 CB LEU A 13 -0.545 -7.394 -5.216 1.00 0.74 C
ATOM 71 CG LEU A 13 -1.354 -8.558 -5.825 1.00 0.74 C
ATOM 72 CD1 LEU A 13 -1.357 -9.751 -4.856 1.00 0.74 C
ATOM 73 CD2 LEU A 13 -2.793 -8.151 -6.180 1.00 0.74 C
ATOM 74 N ALA A 14 1.510 -4.950 -5.033 1.00 0.80 N
ATOM 75 CA ALA A 14 2.100 -3.826 -4.340 1.00 0.80 C
ATOM 76 C ALA A 14 2.080 -2.489 -5.070 1.00 0.80 C
ATOM 77 O ALA A 14 1.812 -1.452 -4.472 1.00 0.80 O
ATOM 78 CB ALA A 14 3.539 -4.194 -4.023 1.00 0.80 C
ATOM 79 N ASN A 15 2.340 -2.481 -6.388 1.00 0.75 N
ATOM 80 CA ASN A 15 2.139 -1.334 -7.252 1.00 0.75 C
ATOM 81 C ASN A 15 0.660 -0.912 -7.330 1.00 0.75 C
ATOM 82 O ASN A 15 0.310 0.266 -7.333 1.00 0.75 O
ATOM 83 CB ASN A 15 2.721 -1.677 -8.640 1.00 0.75 C
ATOM 84 CG ASN A 15 2.786 -0.425 -9.501 1.00 0.75 C
ATOM 85 OD1 ASN A 15 3.420 0.567 -9.143 1.00 0.75 O
ATOM 86 ND2 ASN A 15 2.100 -0.448 -10.664 1.00 0.75
N
ATOM 87 N THR A 16 -0.264 -1.885 -7.368 1.00 0.75 N
ATOM 88 CA THR A 16 -1.702 -1.639 -7.239 1.00 0.75 C
ATOM 89 C THR A 16 -2.102 -1.044 -5.891 1.00 0.75 C
ATOM 90 O THR A 16 -2.887 -0.096 -5.825 1.00 0.75 O
ATOM 91 CB THR A 16 -2.510 -2.892 -7.528 1.00 0.75 C
ATOM 92 OG1 THR A 16 -2.318 -3.274 -8.883 1.00 0.75 O
ATOM 93 CG2 THR A 16 -4.016 -2.666 -7.365 1.00 0.75 C
ATOM 94 N ILE A 17 -1.528 -1.543 -4.769 1.00 0.69 N
ATOM 95 CA ILE A 17 -1.676 -0.970 -3.424 1.00 0.69 C
ATOM 96 C ILE A 17 -1.184 0.439 -3.400 1.00 0.69 C
ATOM 97 O ILE A 17 -1.841 1.343 -2.892 1.00 0.69 O
ATOM 98 CB ILE A 17 -0.878 -1.709 -2.344 1.00 0.69 C
ATOM 99 CG1 ILE A 17 -1.422 -3.121 -2.103 1.00 0.69 C

ATOM 100 CG2 ILE A 17 -0.855 -0.967 -0.982 1.00 0.69 C
ATOM 101 CD1 ILE A 17 -2.854 -3.128 -1.569 1.00 0.69 C
ATOM 102 N LEU A 18 -0.019 0.654 -4.015 1.00 0.71 N
ATOM 103 CA LEU A 18 0.584 1.939 -4.108 1.00 0.71 C
ATOM 104 C LEU A 18 -0.272 2.977 -4.796 1.00 0.71 C
ATOM 105 O LEU A 18 -0.383 4.117 -4.348 1.00 0.71 O
ATOM 106 CB LEU A 18 1.861 1.830 -4.907 1.00 0.71 C
ATOM 107 CG LEU A 18 2.514 3.186 -5.125 1.00 0.71 C
ATOM 108 CD1 LEU A 18 2.984 3.670 -3.759 1.00 0.71 C
ATOM 109 CD2 LEU A 18 3.571 2.978 -6.199 1.00 0.71 C
ATOM 110 N GLY A 19 -0.909 2.601 -5.918 1.00 0.75 N
ATOM 111 CA GLY A 19 -1.873 3.471 -6.562 1.00 0.75 C
ATOM 112 C GLY A 19 -3.050 3.777 -5.676 1.00 0.75 C
ATOM 113 O GLY A 19 -3.453 4.928 -5.567 1.00 0.75 O
ATOM 114 N ALA A 20 -3.604 2.780 -4.962 1.00 0.66 N
ATOM 115 CA ALA A 20 -4.708 3.019 -4.056 1.00 0.66 C
ATOM 116 C ALA A 20 -4.378 3.942 -2.877 1.00 0.66 C
ATOM 117 O ALA A 20 -5.067 4.941 -2.691 1.00 0.66 O
ATOM 118 CB ALA A 20 -5.274 1.675 -3.559 1.00 0.66 C
ATOM 119 N ILE A 21 -3.272 3.682 -2.132 1.00 0.62 N
ATOM 120 CA ILE A 21 -2.791 4.477 -0.993 1.00 0.62 C
ATOM 121 C ILE A 21 -2.391 5.888 -1.377 1.00 0.62 C
ATOM 122 O ILE A 21 -2.423 6.804 -0.569 1.00 0.62 O
ATOM 123 CB ILE A 21 -1.640 3.825 -0.210 1.00 0.62 C
ATOM 124 CG1 ILE A 21 -0.348 3.702 -1.047 1.00 0.62 C
ATOM 125 CG2 ILE A 21 -2.144 2.470 0.326 1.00 0.62 C
ATOM 126 CD1 ILE A 21 0.855 3.117 -0.309 1.00 0.62 C
ATOM 127 N ALA A 22 -1.962 6.084 -2.635 1.00 0.68 N
ATOM 128 CA ALA A 22 -1.636 7.378 -3.178 1.00 0.68 C
ATOM 129 C ALA A 22 -2.834 8.214 -3.619 1.00 0.68 C
ATOM 130 O ALA A 22 -2.795 9.441 -3.566 1.00 0.68 O
ATOM 131 CB ALA A 22 -0.744 7.154 -4.410 1.00 0.68 C
ATOM 132 N VAL A 23 -3.879 7.561 -4.169 1.00 0.56 N
ATOM 133 CA VAL A 23 -5.071 8.221 -4.688 1.00 0.56 C
ATOM 134 C VAL A 23 -6.101 8.539 -3.629 1.00 0.56 C
ATOM 135 O VAL A 23 -6.694 9.618 -3.617 1.00 0.56 O
ATOM 136 CB VAL A 23 -5.753 7.374 -5.763 1.00 0.56 C
ATOM 137 CG1 VAL A 23 -7.117 7.961 -6.193 1.00 0.56 C
ATOM 138 CG2 VAL A 23 -4.848 7.331 -7.004 1.00 0.56 C
ATOM 139 N GLY A 24 -6.410 7.583 -2.734 1.00 0.51 N
ATOM 140 CA GLY A 24 -7.413 7.838 -1.716 1.00 0.51 C
ATOM 141 C GLY A 24 -6.818 8.583 -0.559 1.00 0.51 C
ATOM 142 O GLY A 24 -5.615 8.576 -0.351 1.00 0.51 O
ATOM 143 N ASN A 25 -7.691 9.234 0.225 1.00 0.40 N
ATOM 144 CA ASN A 25 -7.292 10.152 1.270 1.00 0.40 C
ATOM 145 C ASN A 25 -7.539 9.542 2.635 1.00 0.40 C
ATOM 146 O ASN A 25 -6.645 9.275 3.426 1.00 0.40 O
ATOM 147 CB ASN A 25 -8.158 11.435 1.165 1.00 0.40 C
ATOM 148 CG ASN A 25 -7.773 12.248 -0.066 1.00 0.40 C
ATOM 149 OD1 ASN A 25 -6.626 12.323 -0.488 1.00 0.40
O
ATOM 150 ND2 ASN A 25 -8.774 12.944 -0.658 1.00 0.40
N
ATOM 151 N LEU A 26 -8.824 9.343 2.988 1.00 0.34 N
ATOM 152 CA LEU A 26 -9.196 8.756 4.255 1.00 0.34 C
ATOM 153 C LEU A 26 -8.717 7.318 4.416 1.00 0.34 C
ATOM 154 O LEU A 26 -8.910 6.481 3.538 1.00 0.34 O
ATOM 155 CB LEU A 26 -10.730 8.789 4.434 1.00 0.34 C
ATOM 156 CG LEU A 26 -11.342 10.199 4.522 1.00 0.34
C
ATOM 157 CD1 LEU A 26 -12.874 10.100 4.488 1.00 0.34
C
ATOM 158 CD2 LEU A 26 -10.879 10.946 5.781 1.00 0.34
C
ATOM 159 N ALA A 27 -8.131 6.977 5.583 1.00 0.53 N
ATOM 160 CA ALA A 27 -7.590 5.657 5.844 1.00 0.53 C
ATOM 161 C ALA A 27 -8.612 4.529 5.739 1.00 0.53 C
ATOM 162 O ALA A 27 -8.342 3.459 5.216 1.00 0.53 O
ATOM 163 CB ALA A 27 -6.971 5.634 7.255 1.00 0.53 C
ATOM 164 N SER A 28 -9.845 4.744 6.220 1.00 0.42 N
ATOM 165 CA SER A 28 -10.942 3.799 6.067 1.00 0.42 C
ATOM 166 C SER A 28 -11.363 3.552 4.620 1.00 0.42 C
ATOM 167 O SER A 28 -11.683 2.430 4.243 1.00 0.42 O
ATOM 168 CB SER A 28 -12.169 4.237 6.906 1.00 0.42 C
ATOM 169 OG SER A 28 -12.584 5.559 6.557 1.00 0.42 O
ATOM 170 N TRP A 29 -11.357 4.607 3.778 1.00 0.36 N
ATOM 171 CA TRP A 29 -11.642 4.561 2.351 1.00 0.36 C
ATOM 172 C TRP A 29 -10.618 3.747 1.556 1.00 0.36 C
ATOM 173 O TRP A 29 -10.949 2.965 0.666 1.00 0.36 O
ATOM 174 CB TRP A 29 -11.715 6.013 1.813 1.00 0.36 C
ATOM 175 CG TRP A 29 -12.090 6.136 0.352 1.00 0.36 C
ATOM 176 CD1 TRP A 29 -11.284 6.288 -0.742 1.00 0.36
C
ATOM 177 CD2 TRP A 29 -13.428 6.008 -0.136 1.00 0.36
C
ATOM 178 NE1 TRP A 29 -12.042 6.298 -1.891 1.00 0.36
N
ATOM 179 CE2 TRP A 29 -13.363 6.118 -1.542 1.00 0.36 C
ATOM 180 CE3 TRP A 29 -14.635 5.795 0.519 1.00 0.36 C
ATOM 181 CZ2 TRP A 29 -14.512 6.027 -2.311 1.00 0.36 C
ATOM 182 CZ3 TRP A 29 -15.795 5.711 -0.258 1.00 0.36 C
ATOM 183 CH2 TRP A 29 -15.737 5.830 -1.654 1.00 0.36
C
ATOM 184 N LEU A 30 -9.327 3.903 1.901 1.00 0.47 N
ATOM 185 CA LEU A 30 -8.200 3.183 1.340 1.00 0.47 C
ATOM 186 C LEU A 30 -8.189 1.664 1.577 1.00 0.47 C
ATOM 187 O LEU A 30 -7.350 0.953 1.026 1.00 0.47 O
ATOM 188 CB LEU A 30 -6.889 3.779 1.916 1.00 0.47 C
ATOM 189 CG LEU A 30 -6.500 5.186 1.436 1.00 0.47 C
ATOM 190 CD1 LEU A 30 -5.231 5.713 2.134 1.00 0.47 C
ATOM 191 CD2 LEU A 30 -6.240 5.047 -0.054 1.00 0.47 C
ATOM 192 N LEU A 31 -9.142 1.118 2.367 1.00 0.46 N
ATOM 193 CA LEU A 31 -9.217 -0.293 2.717 1.00 0.46 C
ATOM 194 C LEU A 31 -10.094 -1.078 1.759 1.00 0.46 C
ATOM 195 O LEU A 31 -10.405 -2.243 2.008 1.00 0.46 O
ATOM 196 CB LEU A 31 -9.794 -0.525 4.141 1.00 0.46 C
ATOM 197 CG LEU A 31 -8.912 -0.006 5.288 1.00 0.46 C
ATOM 198 CD1 LEU A 31 -9.661 -0.082 6.625 1.00 0.46 C
ATOM 199 CD2 LEU A 31 -7.615 -0.810 5.400 1.00 0.46 C
ATOM 200 N ALA A 32 -10.505 -0.497 0.607 1.00 0.47 N
ATOM 201 CA ALA A 32 -11.161 -1.251 -0.449 1.00 0.47
C
ATOM 202 C ALA A 32 -10.151 -2.125 -1.182 1.00 0.47 C
ATOM 203 O ALA A 32 -9.722 -1.877 -2.310 1.00 0.47 O
ATOM 204 CB ALA A 32 -11.953 -0.368 -1.434 1.00 0.47
C
ATOM 205 N LEU A 33 -9.711 -3.188 -0.502 1.00 0.48 N
ATOM 206 CA LEU A 33 -8.689 -4.066 -0.987 1.00 0.48 C
ATOM 207 C LEU A 33 -9.102 -4.972 -2.154 1.00 0.48 C
ATOM 208 O LEU A 33 -9.581 -6.091 -1.989 1.00 0.48 O
ATOM 209 CB LEU A 33 -8.044 -4.847 0.181 1.00 0.48 C
ATOM 210 CG LEU A 33 -7.443 -3.963 1.298 1.00 0.48 C
ATOM 211 CD1 LEU A 33 -6.979 -4.889 2.425 1.00 0.48 C
ATOM 212 CD2 LEU A 33 -6.313 -3.028 0.829 1.00 0.48 C
ATOM 213 N VAL A 34 -8.768 -4.506 -3.382 1.00 0.53 N
ATOM 214 CA VAL A 34 -8.574 -5.320 -4.595 1.00 0.53 C
ATOM 215 C VAL A 34 -7.645 -6.514 -4.476 1.00 0.53 C
ATOM 216 O VAL A 34 -7.989 -7.522 -5.094 1.00 0.53 O
ATOM 217 CB VAL A 34 -8.124 -4.559 -5.855 1.00 0.53 C
ATOM 218 CG1 VAL A 34 -8.119 -5.483 -7.108 1.00 0.53
C
ATOM 219 CG2 VAL A 34 -9.082 -3.386 -6.099 1.00 0.53
C
ATOM 220 N PRO A 35 -6.518 -6.550 -3.755 1.00 0.62 N
ATOM 221 CA PRO A 35 -5.822 -7.795 -3.474 1.00 0.62 C
ATOM 222 C PRO A 35 -6.704 -9.049 -3.224 1.00 0.62 C
ATOM 223 O PRO A 35 -6.283 -10.136 -3.609 1.00 0.62 O
ATOM 224 CB PRO A 35 -4.845 -7.374 -2.340 1.00 0.62 C

ATOM 225 CG PRO A 35 -4.561 -5.883 -2.502 1.00 0.62 C
ATOM 226 CD PRO A 35 -5.876 -5.398 -3.088 1.00 0.62 C
ATOM 227 N GLY A 36 -7.890 -8.930 -2.556 1.00 0.51 N
ATOM 228 CA GLY A 36 -8.751 -10.010 -2.058 1.00 0.51
C
ATOM 229 C GLY A 36 -8.529 -10.530 -0.622 1.00 0.51 C
ATOM 230 O GLY A 36 -9.325 -11.366 -0.202 1.00 0.51 O
ATOM 231 N PRO A 37 -7.536 -10.094 0.188 1.00 0.50 N
ATOM 232 CA PRO A 37 -7.497 -10.278 1.635 1.00 0.50 C
ATOM 233 C PRO A 37 -7.485 -8.960 2.428 1.00 0.50 C
ATOM 234 O PRO A 37 -7.319 -7.873 1.875 1.00 0.50 O
ATOM 235 CB PRO A 37 -6.109 -10.903 1.802 1.00 0.50 C
ATOM 236 CG PRO A 37 -5.245 -10.098 0.841 1.00 0.50 C
ATOM 237 CD PRO A 37 -6.197 -9.863 -0.300 1.00 0.50 C
ATOM 238 N GLY A 38 -7.617 -9.068 3.775 1.00 0.54 N
ATOM 239 CA GLY A 38 -7.541 -7.985 4.767 1.00 0.54 C
ATOM 240 C GLY A 38 -6.151 -7.723 5.312 1.00 0.54 C
ATOM 241 O GLY A 38 -6.002 -7.287 6.449 1.00 0.54 O
ATOM 242 N TRP A 39 -5.094 -8.030 4.528 1.00 0.52 N
ATOM 243 CA TRP A 39 -3.688 -7.842 4.897 1.00 0.52 C
ATOM 244 C TRP A 39 -3.283 -6.387 5.079 1.00 0.52 C
ATOM 245 O TRP A 39 -2.550 -6.031 6.001 1.00 0.52 O
ATOM 246 CB TRP A 39 -2.717 -8.463 3.846 1.00 0.52 C
ATOM 247 CG TRP A 39 -2.638 -9.979 3.841 1.00 0.52 C
ATOM 248 CD1 TRP A 39 -2.952 -10.820 2.815 1.00 0.52
C
ATOM 249 CD2 TRP A 39 -2.047 -10.812 4.848 1.00 0.52
C
ATOM 250 NE1 TRP A 39 -2.930 -12.123 3.244 1.00 0.52
N
ATOM 251 CE2 TRP A 39 -2.295 -12.151 4.455 1.00 0.52 C
ATOM 252 CE3 TRP A 39 -1.296 -10.527 5.982 1.00 0.52 C
ATOM 253 CZ2 TRP A 39 -1.823 -13.205 5.201 1.00 0.52 C
ATOM 254 CZ3 TRP A 39 -0.716 -11.604 6.664 1.00 0.52 C
ATOM 255 CH2 TRP A 39 -0.983 -12.927 6.284 1.00 0.52
C
ATOM 256 N ALA A 40 -3.743 -5.491 4.187 1.00 0.68 N
ATOM 257 CA ALA A 40 -3.446 -4.086 4.306 1.00 0.68 C
ATOM 258 C ALA A 40 -4.471 -3.402 5.186 1.00 0.68 C
ATOM 259 O ALA A 40 -5.478 -2.876 4.730 1.00 0.68 O
ATOM 260 CB ALA A 40 -3.373 -3.411 2.928 1.00 0.68 C
ATOM 261 N THR A 41 -4.212 -3.406 6.499 1.00 0.64 N
ATOM 262 CA THR A 41 -5.039 -2.812 7.529 1.00 0.64 C
ATOM 263 C THR A 41 -4.749 -1.336 7.645 1.00 0.64 C
ATOM 264 O THR A 41 -3.927 -0.778 6.923 1.00 0.64 O
ATOM 265 CB THR A 41 -4.832 -3.489 8.878 1.00 0.64 C
ATOM 266 OG1 THR A 41 -3.450 -3.561 9.216 1.00 0.64
O
ATOM 267 CG2 THR A 41 -5.366 -4.918 8.751 1.00 0.64 C
ATOM 268 N LYS A 42 -5.417 -0.632 8.581 1.00 0.63 N
ATOM 269 CA LYS A 42 -5.145 0.770 8.863 1.00 0.63 C
ATOM 270 C LYS A 42 -3.685 1.061 9.199 1.00 0.63 C
ATOM 271 O LYS A 42 -3.139 2.085 8.798 1.00 0.63 O
ATOM 272 CB LYS A 42 -5.993 1.234 10.063 1.00 0.63 C
ATOM 273 CG LYS A 42 -7.494 1.304 9.761 1.00 0.63 C
ATOM 274 CD LYS A 42 -8.283 1.763 10.997 1.00 0.63 C
ATOM 275 CE LYS A 42 -9.787 1.868 10.738 1.00 0.63 C
ATOM 276 NZ LYS A 42 -10.505 2.272 11.969 1.00 0.63 N
ATOM 277 N ALA A 43 -3.029 0.133 9.930 1.00 0.73 N
ATOM 278 CA ALA A 43 -1.613 0.174 10.224 1.00 0.73 C
ATOM 279 C ALA A 43 -0.752 0.107 8.966 1.00 0.73 C
ATOM 280 O ALA A 43 0.170 0.897 8.789 1.00 0.73 O
ATOM 281 CB ALA A 43 -1.252 -0.995 11.169 1.00 0.73 C
ATOM 282 N ALA A 44 -1.061 -0.811 8.024 1.00 0.76 N
ATOM 283 CA ALA A 44 -0.361 -0.901 6.760 1.00 0.76 C
ATOM 284 C ALA A 44 -0.566 0.312 5.868 1.00 0.76 C
ATOM 285 O ALA A 44 0.388 0.826 5.295 1.00 0.76 O
ATOM 286 CB ALA A 44 -0.766 -2.174 6.000 1.00 0.76 C
ATOM 287 N LEU A 45 -1.803 0.828 5.758 1.00 0.69 N
ATOM 288 CA LEU A 45 -2.087 2.040 5.016 1.00 0.69 C
ATOM 289 C LEU A 45 -1.399 3.289 5.523 1.00 0.69 C
ATOM 290 O LEU A 45 -0.764 4.012 4.758 1.00 0.69 O
ATOM 291 CB LEU A 45 -3.575 2.361 5.124 1.00 0.69 C
ATOM 292 CG LEU A 45 -4.513 1.405 4.397 1.00 0.69 C
ATOM 293 CD1 LEU A 45 -5.893 1.955 4.704 1.00 0.69 C
ATOM 294 CD2 LEU A 45 -4.280 1.404 2.886 1.00 0.69 C
ATOM 295 N ALA A 46 -1.487 3.545 6.845 1.00 0.76 N
ATOM 296 CA ALA A 46 -0.866 4.687 7.477 1.00 0.76 C
ATOM 297 C ALA A 46 0.646 4.648 7.343 1.00 0.76 C
ATOM 298 O ALA A 46 1.292 5.631 6.986 1.00 0.76 O
ATOM 299 CB ALA A 46 -1.233 4.710 8.974 1.00 0.76 C
ATOM 300 N THR A 47 1.253 3.469 7.585 1.00 0.81 N
ATOM 301 CA THR A 47 2.683 3.277 7.385 1.00 0.81 C
ATOM 302 C THR A 47 3.089 3.429 5.934 1.00 0.81 C
ATOM 303 O THR A 47 4.021 4.162 5.623 1.00 0.81 O
ATOM 304 CB THR A 47 3.168 1.923 7.880 1.00 0.81 C
ATOM 305 OG1 THR A 47 2.894 1.788 9.265 1.00 0.81 O
ATOM 306 CG2 THR A 47 4.688 1.764 7.756 1.00 0.81 C
ATOM 307 N ALA A 48 2.369 2.792 4.988 1.00 0.82 N
ATOM 308 CA ALA A 48 2.670 2.856 3.576 1.00 0.82 C
ATOM 309 C ALA A 48 2.568 4.257 2.989 1.00 0.82 C
ATOM 310 O ALA A 48 3.484 4.696 2.302 1.00 0.82 O
ATOM 311 CB ALA A 48 1.769 1.870 2.813 1.00 0.82 C
ATOM 312 N GLU A 49 1.508 5.035 3.295 1.00 0.73 N
ATOM 313 CA GLU A 49 1.408 6.421 2.863 1.00 0.73 C
ATOM 314 C GLU A 49 2.523 7.324 3.431 1.00 0.73 C
ATOM 315 O GLU A 49 3.089 8.194 2.765 1.00 0.73 O
ATOM 316 CB GLU A 49 0.011 7.013 3.105 1.00 0.73 C
ATOM 317 CG GLU A 49 -0.139 8.391 2.415 1.00 0.73 C
ATOM 318 CD GLU A 49 -1.492 9.059 2.640 1.00 0.73 C
ATOM 319 OE1 GLU A 49 -2.388 8.421 3.246 1.00 0.73 O
ATOM 320 OE2 GLU A 49 -1.581 10.262 2.268 1.00 0.73
O
ATOM 321 N THR A 50 2.920 7.100 4.695 1.00 0.80 N
ATOM 322 CA THR A 50 4.081 7.746 5.321 1.00 0.80 C
ATOM 323 C THR A 50 5.411 7.377 4.673 1.00 0.80 C
ATOM 324 O THR A 50 6.264 8.234 4.435 1.00 0.80 O
ATOM 325 CB THR A 50 4.129 7.494 6.819 1.00 0.80 C
ATOM 326 OG1 THR A 50 2.978 8.068 7.425 1.00 0.80 O
ATOM 327 CG2 THR A 50 5.315 8.186 7.504 1.00 0.80 C
ATOM 328 N ILE A 51 5.614 6.090 4.315 1.00 0.73 N
ATOM 329 CA ILE A 51 6.752 5.618 3.523 1.00 0.73 C
ATOM 330 C ILE A 51 6.796 6.274 2.155 1.00 0.73 C
ATOM 331 O ILE A 51 7.835 6.780 1.731 1.00 0.73 O
ATOM 332 CB ILE A 51 6.761 4.097 3.366 1.00 0.73 C
ATOM 333 CG1 ILE A 51 7.002 3.415 4.728 1.00 0.73 C
ATOM 334 CG2 ILE A 51 7.856 3.635 2.373 1.00 0.73 C
ATOM 335 CD1 ILE A 51 6.733 1.909 4.684 1.00 0.73 C
ATOM 336 N VAL A 52 5.659 6.365 1.432 1.00 0.75 N
ATOM 337 CA VAL A 52 5.671 6.996 0.117 1.00 0.75 C
ATOM 338 C VAL A 52 5.878 8.492 0.150 1.00 0.75 C
ATOM 339 O VAL A 52 6.277 9.093 -0.844 1.00 0.75 O
ATOM 340 CB VAL A 52 4.466 6.714 -0.771 1.00 0.75 C
ATOM 341 CG1 VAL A 52 4.249 5.207 -0.854 1.00 0.75 C
ATOM 342 CG2 VAL A 52 3.194 7.357 -0.221 1.00 0.75 C
ATOM 343 N LYS A 53 5.601 9.127 1.300 1.00 0.72 N
ATOM 344 CA LYS A 53 5.924 10.510 1.539 1.00 0.72 C
ATOM 345 C LYS A 53 7.411 10.808 1.723 1.00 0.72 C
ATOM 346 O LYS A 53 7.915 11.775 1.160 1.00 0.72 O
ATOM 347 CB LYS A 53 5.134 10.997 2.777 1.00 0.72 C
ATOM 348 CG LYS A 53 5.309 12.488 3.109 1.00 0.72 C
ATOM 349 CD LYS A 53 4.847 13.438 1.988 1.00 0.72 C
ATOM 350 CE LYS A 53 3.352 13.389 1.661 1.00 0.72 C
ATOM 351 NZ LYS A 53 2.576 13.875 2.820 1.00 0.72 N
ATOM 352 N HIS A 54 8.137 10.003 2.532 1.00 0.70 N
ATOM 353 CA HIS A 54 9.556 10.224 2.787 1.00 0.70 C
ATOM 354 C HIS A 54 10.472 9.542 1.775 1.00 0.70 C
ATOM 355 O HIS A 54 11.336 10.165 1.166 1.00 0.70 O

ATOM 356 CB HIS A 54 9.922 9.707 4.205 1.00 0.70 C
ATOM 357 CG HIS A 54 11.365 9.869 4.579 1.00 0.70 C
ATOM 358 ND1 HIS A 54 11.866 11.143 4.737 1.00 0.70 N
ATOM 359 CD2 HIS A 54 12.353 8.950 4.737 1.00 0.70 C
ATOM 360 CE1 HIS A 54 13.149 10.979 4.980 1.00 0.70 C
ATOM 361 NE2 HIS A 54 13.499 9.670 4.993 1.00 0.70 N
ATOM 362 N GLU A 55 10.306 8.221 1.576 1.00 0.69 N
ATOM 363 CA GLU A 55 11.189 7.419 0.745 1.00 0.69 C
ATOM 364 C GLU A 55 10.711 7.350 -0.675 1.00 0.69 C
ATOM 365 O GLU A 55 11.478 7.397 -1.636 1.00 0.69 O
ATOM 366 CB GLU A 55 11.258 5.981 1.285 1.00 0.69 C
ATOM 367 CG GLU A 55 11.956 5.912 2.655 1.00 0.69 C
ATOM 368 CD GLU A 55 12.040 4.499 3.224 1.00 0.69 C
ATOM 369 OE1 GLU A 55 11.447 3.569 2.620 1.00 0.69
O
ATOM 370 OE2 GLU A 55 12.695 4.354 4.287 1.00 0.69
O
ATOM 371 N GLY A 56 9.387 7.244 -0.839 1.00 0.78 N
ATOM 372 CA GLY A 56 8.785 7.281 -2.145 1.00 0.78 C
ATOM 373 C GLY A 56 7.963 6.085 -2.426 1.00 0.78 C
ATOM 374 O GLY A 56 7.970 5.058 -1.752 1.00 0.78 O
ATOM 375 N LYS A 57 7.193 6.218 -3.501 1.00 0.71 N
ATOM 376 CA LYS A 57 6.244 5.237 -3.943 1.00 0.71 C
ATOM 377 C LYS A 57 6.852 3.851 -4.196 1.00 0.71 C
ATOM 378 O LYS A 57 6.369 2.840 -3.693 1.00 0.71 O
ATOM 379 CB LYS A 57 5.578 5.824 -5.204 1.00 0.71 C
ATOM 380 CG LYS A 57 4.579 6.959 -4.925 1.00 0.71 C
ATOM 381 CD LYS A 57 3.755 7.242 -6.192 1.00 0.71 C
ATOM 382 CE LYS A 57 2.664 8.291 -5.978 1.00 0.71 C
ATOM 383 NZ LYS A 57 2.026 8.658 -7.261 1.00 0.71 N
ATOM 384 N ALA A 58 7.998 3.796 -4.904 1.00 0.73 N
ATOM 385 CA ALA A 58 8.769 2.597 -5.168 1.00 0.73 C
ATOM 386 C ALA A 58 9.374 1.902 -3.939 1.00 0.73 C
ATOM 387 O ALA A 58 9.546 0.684 -3.921 1.00 0.73 O
ATOM 388 CB ALA A 58 9.845 2.930 -6.223 1.00 0.73 C
ATOM 389 N ALA A 59 9.703 2.658 -2.872 1.00 0.75 N
ATOM 390 CA ALA A 59 10.153 2.108 -1.609 1.00 0.75 C
ATOM 391 C ALA A 59 9.061 1.310 -0.890 1.00 0.75 C
ATOM 392 O ALA A 59 9.268 0.178 -0.454 1.00 0.75 O
ATOM 393 CB ALA A 59 10.608 3.275 -0.724 1.00 0.75 C
ATOM 394 N ALA A 60 7.832 1.877 -0.829 1.00 0.79 N
ATOM 395 CA ALA A 60 6.671 1.247 -0.221 1.00 0.79 C
ATOM 396 C ALA A 60 6.136 0.059 -1.003 1.00 0.79 C
ATOM 397 O ALA A 60 5.514 -0.831 -0.435 1.00 0.79 O
ATOM 398 CB ALA A 60 5.503 2.232 -0.055 1.00 0.79 C
ATOM 399 N ILE A 61 6.372 0.016 -2.337 1.00 0.70 N
ATOM 400 CA ILE A 61 6.154 -1.176 -3.156 1.00 0.70 C
ATOM 401 C ILE A 61 6.993 -2.345 -2.659 1.00 0.70 C
ATOM 402 O ILE A 61 6.480 -3.439 -2.432 1.00 0.70 O
ATOM 403 CB ILE A 61 6.471 -0.929 -4.644 1.00 0.70 C
ATOM 404 CG1 ILE A 61 5.414 -0.016 -5.300 1.00 0.70 C
ATOM 405 CG2 ILE A 61 6.559 -2.246 -5.461 1.00 0.70 C
ATOM 406 CD1 ILE A 61 5.777 0.388 -6.736 1.00 0.70 C
ATOM 407 N ALA A 62 8.309 -2.139 -2.444 1.00 0.73 N
ATOM 408 CA ALA A 62 9.190 -3.161 -1.921 1.00 0.73 C
ATOM 409 C ALA A 62 8.861 -3.576 -0.487 1.00 0.73 C
ATOM 410 O ALA A 62 8.795 -4.770 -0.188 1.00 0.73 O
ATOM 411 CB ALA A 62 10.654 -2.695 -2.067 1.00 0.73 C
ATOM 412 N TRP A 63 8.591 -2.599 0.400 1.00 0.65 N
ATOM 413 CA TRP A 63 8.145 -2.812 1.767 1.00 0.65 C
ATOM 414 C TRP A 63 6.893 -3.747 1.873 1.00 0.65 C
ATOM 415 O TRP A 63 5.913 -3.583 1.089 1.00 0.65 O
ATOM 416 CB TRP A 63 7.961 -1.418 2.442 1.00 0.65 C
ATOM 417 CG TRP A 63 7.620 -1.450 3.920 1.00 0.65 C
ATOM 418 CD1 TRP A 63 8.446 -1.520 5.006 1.00 0.65 C
ATOM 419 CD2 TRP A 63 6.278 -1.449 4.426 1.00 0.65 C
ATOM 420 NE1 TRP A 63 7.702 -1.589 6.166 1.00 0.65 N
ATOM 421 CE2 TRP A 63 6.368 -1.556 5.831 1.00 0.65 C
ATOM 422 CE3 TRP A 63 5.047 -1.389 3.785 1.00 0.65 C
ATOM 423 CZ2 TRP A 63 5.222 -1.628 6.611 1.00 0.65 C
ATOM 424 CZ3 TRP A 63 3.894 -1.448 4.575 1.00 0.65 C
ATOM 425 CH2 TRP A 63 3.978 -1.571 5.968 1.00 0.65 C
ATOM 426 OXT TRP A 63 6.946 -4.704 2.692 1.00 0.65 O
TER 427 TRP A 63
END

B)

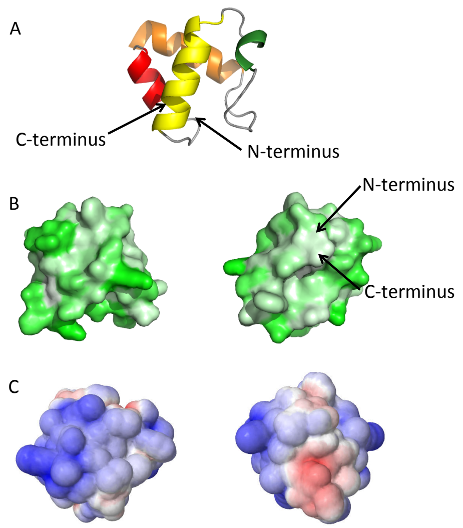

Figure S4- Structural models of leucocyclicin. A) Ribbon diagram depicting the saposin-like fold. The C- and N- termini are explicitly shown. Hydrophobic surface (B) and electrostatic potential surface (C) maps of leucocyclicin. Orientation peptide backbone in surface filling diagrams, B and C, correlate to one another.

5. Garvacin ML

A) PDB file for model of garvacin ML. Structure modeled after AS-48 using SWISS-MODEL[84]

```
TITLE     SWISS-MODEL SERVER (https://swissmodel.expasy.org)
TITLE    2 Untitled Project
EXPDTA    THEORETICAL MODEL (SWISS-MODEL SERVER)
AUTHOR    SWISS-MODEL SERVER (SEE REFERENCE IN JRNL
Records)
REVDAT   1   11-AUG-16 1MOD    1       22:28
JRNL        AUTH
M.BIASINI,S.BIENERT,A.WATERHOUSE,K.ARNOLD,G.STUDER,
JRNL        AUTH 2 T.SCHMIDT,F.KIEFER,T.GALLO
CASSARINO,M.BERTONI,L.BORDOLI,
JRNL        AUTH 3 T.SCHWEDE
JRNL        TITL   SWISS-MODEL: MODELLING PROTEIN TERTIARY
AND QUATERNARY
JRNL        TITL 2 STRUCTURE USING EVOLUTIONARY
INFORMATION
JRNL        REF    NUCLEIC.ACIDS.RES                        2014
JRNL        REFN                   ISSN 0305-1048
JRNL        PMID   24782522
JRNL        DOI    10.1093/nar/gku340
REMARK   3
REMARK   3 MODEL INFORMATION
REMARK   3  ENGIN   PROMOD3
REMARK   3  VERSN   1.0.0
REMARK   3  OSTAT   MONOMER
REMARK   3  OSRSN   PREDICTION
REMARK   3  GMQE    0.65
REMARK   3  QMN4    -1.90
REMARK   3
REMARK   3 TEMPLATE 1
```

```
REMARK   3  PDBID   4rgd
REMARK   3  CHAIN   A
REMARK   3  MMCIF   A
REMARK   3  PDBV    2016-07-29
REMARK   3  SMTLE   4rgd.1.A
REMARK   3  SMTLV   2016-08-03
REMARK   3  MTHD    X-RAY DIFFRACTION 1.20 A
REMARK   3  FOUND   HHblits
REMARK   3  GMQE    0.65
REMARK   3  SIM     0.37
REMARK   3  SID     35.71
REMARK   3  OSTAT   homo-dimer
REMARK   3  LIGND   CIT
ATOM      1  N   THR A   4      55.986  -2.970  89.102  1.00  0.34           N
ATOM      2  CA  THR A   4      55.526  -2.404  87.777  1.00  0.34           C
ATOM      3  C   THR A   4      56.617  -2.286  86.715  1.00  0.34           C
ATOM      4  O   THR A   4      56.298  -2.050  85.596  1.00  0.34           O
ATOM      5  CB  THR A   4      54.855  -1.023  87.967  1.00  0.34           C
ATOM      6  OG1 THR A   4      55.684  -0.173  88.743  1.00  0.34           O
ATOM      7  CG2 THR A   4      53.540  -1.159  88.758  1.00  0.34           C
ATOM      8  N   GLY A   5      57.944  -2.498  87.008  1.00  0.40           N
ATOM      9  CA  GLY A   5      58.934  -2.311  85.933  1.00  0.40           C
ATOM     10  C   GLY A   5      59.203  -0.881  85.550  1.00  0.40           C
ATOM     11  O   GLY A   5      59.780  -0.607  84.506  1.00  0.40           O
ATOM     12  N   MET A   6      58.809   0.081  86.409  1.00  0.53           N
ATOM     13  CA  MET A   6      59.205   1.459  86.267  1.00  0.53           C
ATOM     14  C   MET A   6      60.640   1.570  86.645  1.00  0.53           C
ATOM     15  O   MET A   6      61.020   1.242  87.759  1.00  0.53           O
ATOM     16  CB  MET A   6      58.385   2.407  87.176  1.00  0.53           C
```

ATOM 17 CG MET A 6 56.907 2.378 86.776 1.00 0.53 C
ATOM 18 SD MET A 6 56.614 2.668 85.020 1.00 0.53 S
ATOM 19 CE MET A 6 57.503 4.240 84.995 1.00 0.53 C
ATOM 20 N ALA A 7 61.481 2.006 85.688 1.00 0.66 N
ATOM 21 CA ALA A 7 62.888 2.200 85.943 1.00 0.66 C
ATOM 22 C ALA A 7 63.110 3.205 87.073 1.00 0.66 C
ATOM 23 O ALA A 7 62.441 4.238 87.123 1.00 0.66 O
ATOM 24 CB ALA A 7 63.618 2.670 84.660 1.00 0.66 C
ATOM 25 N ALA A 8 64.047 2.947 88.014 1.00 0.72 N
ATOM 26 CA ALA A 8 64.193 3.736 89.230 1.00 0.72 C
ATOM 27 C ALA A 8 64.433 5.223 88.987 1.00 0.72 C
ATOM 28 O ALA A 8 63.907 6.076 89.688 1.00 0.72 O
ATOM 29 CB ALA A 8 65.318 3.164 90.121 1.00 0.72 C
ATOM 30 N GLY A 9 65.202 5.573 87.930 1.00 0.79 N
ATOM 31 CA GLY A 9 65.378 6.960 87.505 1.00 0.79 C
ATOM 32 C GLY A 9 64.102 7.644 87.070 1.00 0.79 C
ATOM 33 O GLY A 9 63.872 8.792 87.433 1.00 0.79 O
ATOM 34 N VAL A 10 63.214 6.938 86.339 1.00 0.74 N
ATOM 35 CA VAL A 10 61.884 7.413 85.976 1.00 0.74 C
ATOM 36 C VAL A 10 60.982 7.511 87.196 1.00 0.74 C
ATOM 37 O VAL A 10 60.324 8.530 87.400 1.00 0.74 O
ATOM 38 CB VAL A 10 61.231 6.534 84.906 1.00 0.74 C
ATOM 39 CG1 VAL A 10 59.819 7.061 84.547 1.00 0.74
C
ATOM 40 CG2 VAL A 10 62.131 6.544 83.652 1.00 0.74
C
ATOM 41 N ALA A 11 60.966 6.492 88.085 1.00 0.78 N
ATOM 42 CA ALA A 11 60.175 6.481 89.305 1.00 0.78 C
ATOM 43 C ALA A 11 60.522 7.630 90.264 1.00 0.78 C
ATOM 44 O ALA A 11 59.640 8.278 90.817 1.00 0.78 O
ATOM 45 CB ALA A 11 60.329 5.126 90.032 1.00 0.78 C
ATOM 46 N LYS A 12 61.833 7.950 90.424 1.00 0.75 N
ATOM 47 CA LYS A 12 62.322 9.117 91.152 1.00 0.75 C
ATOM 48 C LYS A 12 61.795 10.433 90.584 1.00 0.75 C
ATOM 49 O LYS A 12 61.321 11.291 91.308 1.00 0.75 O
ATOM 50 CB LYS A 12 63.876 9.208 91.088 1.00 0.75 C
ATOM 51 CG LYS A 12 64.606 8.168 91.954 1.00 0.75 C
ATOM 52 CD LYS A 12 66.102 8.010 91.619 1.00 0.75 C
ATOM 53 CE LYS A 12 66.895 9.317 91.722 1.00 0.75 C
ATOM 54 NZ LYS A 12 68.298 9.093 91.316 1.00 0.75 N
ATOM 55 N THR A 13 61.845 10.583 89.235 1.00 0.83 N
ATOM 56 CA THR A 13 61.332 11.751 88.511 1.00 0.83
C
ATOM 57 C THR A 13 59.864 11.946 88.719 1.00 0.83 C
ATOM 58 O THR A 13 59.395 13.057 88.963 1.00 0.83 O
ATOM 59 CB THR A 13 61.559 11.662 87.008 1.00 0.83 C
ATOM 60 OG1 THR A 13 62.951 11.721 86.766 1.00 0.83
O
ATOM 61 CG2 THR A 13 60.943 12.833 86.214 1.00 0.83
C
ATOM 62 N ILE A 14 59.088 10.850 88.671 1.00 0.77 N
ATOM 63 CA ILE A 14 57.683 10.908 88.994 1.00 0.77 C
ATOM 64 C ILE A 14 57.403 11.367 90.406 1.00 0.77 C
ATOM 65 O ILE A 14 56.600 12.266 90.622 1.00 0.77 O
ATOM 66 CB ILE A 14 57.029 9.541 88.871 1.00 0.77 C
ATOM 67 CG1 ILE A 14 57.085 9.064 87.398 1.00 0.77 C
ATOM 68 CG2 ILE A 14 55.606 9.654 89.489 1.00 0.77 C
ATOM 69 CD1 ILE A 14 56.231 9.880 86.440 1.00 0.77 C
ATOM 70 N VAL A 15 58.038 10.725 91.414 1.00 0.81 N
ATOM 71 CA VAL A 15 57.756 10.990 92.819 1.00 0.81
C
ATOM 72 C VAL A 15 58.099 12.432 93.124 1.00 0.81 C
ATOM 73 O VAL A 15 57.383 13.116 93.869 1.00 0.81 O
ATOM 74 CB VAL A 15 58.440 9.989 93.751 1.00 0.81 C
ATOM 75 CG1 VAL A 15 58.298 10.437 95.222 1.00 0.81
C
ATOM 76 CG2 VAL A 15 57.773 8.606 93.568 1.00 0.81
C
ATOM 77 N ASN A 16 59.148 12.974 92.491 1.00 0.82 N
ATOM 78 CA ASN A 16 59.478 14.383 92.532 1.00 0.82
C
ATOM 79 C ASN A 16 58.404 15.312 91.948 1.00 0.82 C
ATOM 80 O ASN A 16 58.130 16.371 92.474 1.00 0.82 O
ATOM 81 CB ASN A 16 60.833 14.676 91.851 1.00 0.82 C
ATOM 82 CG ASN A 16 61.962 14.110 92.710 1.00 0.82
C
ATOM 83 OD1 ASN A 16 61.818 13.773 93.871 1.00 0.82
O
ATOM 84 ND2 ASN A 16 63.178 14.056 92.104 1.00 0.82
N
ATOM 85 N ALA A 17 57.746 14.921 90.829 1.00 0.84 N
ATOM 86 CA ALA A 17 56.565 15.627 90.353 1.00 0.84
C
ATOM 87 C ALA A 17 55.393 15.578 91.338 1.00 0.84 C
ATOM 88 O ALA A 17 54.733 16.587 91.587 1.00 0.84 O
ATOM 89 CB ALA A 17 56.112 15.057 88.989 1.00 0.84
C
ATOM 90 N VAL A 18 55.135 14.415 91.964 1.00 0.80 N
ATOM 91 CA VAL A 18 54.136 14.243 93.014 1.00 0.80
C
ATOM 92 C VAL A 18 54.393 15.107 94.245 1.00 0.80 C
ATOM 93 O VAL A 18 53.485 15.756 94.762 1.00 0.80 O
ATOM 94 CB VAL A 18 54.089 12.786 93.469 1.00 0.80
C
ATOM 95 CG1 VAL A 18 53.212 12.584 94.725 1.00 0.80
C
ATOM 96 CG2 VAL A 18 53.553 11.938 92.302 1.00 0.80
C
ATOM 97 N SER A 19 55.649 15.139 94.745 1.00 0.83 N
ATOM 98 CA SER A 19 56.071 15.920 95.902 1.00 0.83 C
ATOM 99 C SER A 19 56.032 17.420 95.667 1.00 0.83 C
ATOM 100 O SER A 19 55.714 18.185 96.568 1.00 0.83 O
ATOM 101 CB SER A 19 57.469 15.505 96.456 1.00 0.83
C
ATOM 102 OG SER A 19 58.526 15.804 95.545 1.00 0.83
O
ATOM 103 N ALA A 20 56.320 17.863 94.425 1.00 0.82 N
ATOM 104 CA ALA A 20 56.237 19.249 94.020 1.00 0.82
C
ATOM 105 C ALA A 20 54.803 19.713 93.761 1.00 0.82 C
ATOM 106 O ALA A 20 54.554 20.908 93.595 1.00 0.82 O
ATOM 107 CB ALA A 20 57.105 19.441 92.754 1.00 0.82
C
ATOM 108 N GLY A 21 53.815 18.788 93.716 1.00 0.85 N
ATOM 109 CA GLY A 21 52.417 19.111 93.440 1.00 0.85
C
ATOM 110 C GLY A 21 52.148 19.421 91.994 1.00 0.85 C
ATOM 111 O GLY A 21 51.234 20.160 91.669 1.00 0.85 O
ATOM 112 N MET A 22 52.983 18.838 91.106 1.00 0.75
N
ATOM 113 CA MET A 22 52.852 18.881 89.662 1.00 0.75
C
ATOM 114 C MET A 22 51.628 18.141 89.155 1.00 0.75 C
ATOM 115 O MET A 22 50.863 17.511 89.888 1.00 0.75
O
ATOM 116 CB MET A 22 54.115 18.320 88.943 1.00 0.75
C
ATOM 117 CG MET A 22 55.412 19.086 89.275 1.00 0.75
C
ATOM 118 SD MET A 22 55.450 20.831 88.767 1.00 0.75
S
ATOM 119 CE MET A 22 55.504 20.515 86.981 1.00 0.75
C
ATOM 120 N ASP A 23 51.401 18.221 87.840 1.00 0.70 N
ATOM 121 CA ASP A 23 50.156 17.815 87.246 1.00 0.70
C
ATOM 122 C ASP A 23 49.999 16.300 87.134 1.00 0.70 C
ATOM 123 O ASP A 23 50.939 15.567 86.801 1.00 0.70 O

ATOM 124 CB ASP A 23 50.037 18.461 85.841 1.00 0.70
C
ATOM 125 CG ASP A 23 49.961 19.979 85.931 1.00 0.70
C
ATOM 126 OD1 ASP A 23 49.578 20.496 87.005 1.00 0.70
O
ATOM 127 OD2 ASP A 23 50.282 20.619 84.901 1.00 0.70
O
ATOM 128 N ILE A 24 48.759 15.782 87.316 1.00 0.64 N
ATOM 129 CA ILE A 24 48.393 14.400 87.007 1.00 0.64 C
ATOM 130 C ILE A 24 48.657 14.059 85.547 1.00 0.64 C
ATOM 131 O ILE A 24 49.160 12.986 85.248 1.00 0.64 O
ATOM 132 CB ILE A 24 46.940 14.062 87.375 1.00 0.64 C
ATOM 133 CG1 ILE A 24 46.811 13.893 88.907 1.00 0.64
C
ATOM 134 CG2 ILE A 24 46.469 12.768 86.653 1.00 0.64
C
ATOM 135 CD1 ILE A 24 45.358 13.696 89.361 1.00 0.64
C
ATOM 136 N ALA A 25 48.369 14.983 84.598 1.00 0.73 N
ATOM 137 CA ALA A 25 48.602 14.755 83.181 1.00 0.73
C
ATOM 138 C ALA A 25 50.076 14.490 82.860 1.00 0.73 C
ATOM 139 O ALA A 25 50.395 13.631 82.042 1.00 0.73 O
ATOM 140 CB ALA A 25 48.062 15.929 82.332 1.00 0.73
C
ATOM 141 N THR A 26 51.000 15.197 83.548 1.00 0.74 N
ATOM 142 CA THR A 26 52.447 14.989 83.477 1.00 0.74
C
ATOM 143 C THR A 26 52.877 13.659 84.044 1.00 0.74 C
ATOM 144 O THR A 26 53.685 12.949 83.461 1.00 0.74 O
ATOM 145 CB THR A 26 53.243 16.065 84.195 1.00 0.74
C
ATOM 146 OG1 THR A 26 52.851 17.328 83.689 1.00 0.74
O
ATOM 147 CG2 THR A 26 54.747 15.936 83.901 1.00 0.74
C
ATOM 148 N ALA A 27 52.313 13.253 85.207 1.00 0.76 N
ATOM 149 CA ALA A 27 52.549 11.925 85.737 1.00 0.76
C
ATOM 150 C ALA A 27 52.056 10.852 84.768 1.00 0.76 C
ATOM 151 O ALA A 27 52.806 9.954 84.398 1.00 0.76 O
ATOM 152 CB ALA A 27 51.881 11.779 87.128 1.00 0.76
C
ATOM 153 N LEU A 28 50.823 10.995 84.231 1.00 0.65 N
ATOM 154 CA LEU A 28 50.260 10.077 83.260 1.00 0.65
C
ATOM 155 C LEU A 28 51.119 9.926 81.999 1.00 0.65 C
ATOM 156 O LEU A 28 51.376 8.819 81.555 1.00 0.65 O
ATOM 157 CB LEU A 28 48.834 10.527 82.837 1.00 0.65
C
ATOM 158 CG LEU A 28 48.159 9.632 81.767 1.00 0.65 C
ATOM 159 CD1 LEU A 28 48.022 8.169 82.225 1.00 0.65
C
ATOM 160 CD2 LEU A 28 46.790 10.198 81.360 1.00 0.65
C
ATOM 161 N SER A 29 51.604 11.053 81.417 1.00 0.73 N
ATOM 162 CA SER A 29 52.467 11.051 80.232 1.00 0.73
C
ATOM 163 C SER A 29 53.841 10.443 80.421 1.00 0.73 C
ATOM 164 O SER A 29 54.351 9.741 79.545 1.00 0.73 O
ATOM 165 CB SER A 29 52.633 12.446 79.556 1.00 0.73
C
ATOM 166 OG SER A 29 53.341 13.386 80.367 1.00 0.73
O
ATOM 167 N LEU A 30 54.508 10.677 81.560 1.00 0.68 N
ATOM 168 CA LEU A 30 55.768 10.032 81.863 1.00 0.68
C
ATOM 169 C LEU A 30 55.637 8.528 82.080 1.00 0.68 C
ATOM 170 O LEU A 30 56.437 7.735 81.601 1.00 0.68 O
ATOM 171 CB LEU A 30 56.359 10.642 83.145 1.00 0.68
C
ATOM 172 CG LEU A 30 56.887 12.085 83.057 1.00 0.68
C
ATOM 173 CD1 LEU A 30 57.280 12.580 84.459 1.00 0.68
C
ATOM 174 CD2 LEU A 30 58.102 12.179 82.129 1.00 0.68
C
ATOM 175 N PHE A 31 54.591 8.109 82.824 1.00 0.56 N
ATOM 176 CA PHE A 31 54.268 6.716 83.038 1.00 0.56 C
ATOM 177 C PHE A 31 53.857 5.958 81.798 1.00 0.56 C
ATOM 178 O PHE A 31 54.279 4.828 81.597 1.00 0.56 O
ATOM 179 CB PHE A 31 53.142 6.568 84.079 1.00 0.56 C
ATOM 180 CG PHE A 31 53.697 6.814 85.446 1.00 0.56 C
ATOM 181 CD1 PHE A 31 53.214 7.778 86.352 1.00 0.56
C
ATOM 182 CD2 PHE A 31 54.655 5.897 85.891 1.00 0.56
C
ATOM 183 CE1 PHE A 31 53.625 7.748 87.689 1.00 0.56
C
ATOM 184 CE2 PHE A 31 55.112 5.898 87.204 1.00 0.56
C
ATOM 185 CZ PHE A 31 54.539 6.773 88.117 1.00 0.56 C
ATOM 186 N SER A 32 53.043 6.581 80.923 1.00 0.58 N
ATOM 187 CA SER A 32 52.675 6.031 79.624 1.00 0.58 C
ATOM 188 C SER A 32 53.883 5.840 78.713 1.00 0.58 C
ATOM 189 O SER A 32 53.973 4.864 77.970 1.00 0.58 O
ATOM 190 CB SER A 32 51.593 6.879 78.893 1.00 0.58 C
ATOM 191 OG SER A 32 52.083 8.140 78.441 1.00 0.58 O
ATOM 192 N GLY A 33 54.868 6.768 78.785 1.00 0.59 N
ATOM 193 CA GLY A 33 56.139 6.684 78.075 1.00 0.59
C
ATOM 194 C GLY A 33 57.054 5.566 78.518 1.00 0.59 C
ATOM 195 O GLY A 33 57.912 5.142 77.752 1.00 0.59 O
ATOM 196 N ALA A 34 56.891 5.052 79.758 1.00 0.53 N
ATOM 197 CA ALA A 34 57.588 3.872 80.257 1.00 0.53
C
ATOM 198 C ALA A 34 57.219 2.552 79.576 1.00 0.53 C
ATOM 199 O ALA A 34 58.073 1.679 79.479 1.00 0.53 O
ATOM 200 CB ALA A 34 57.342 3.626 81.752 1.00 0.53
C
ATOM 201 N PHE A 35 55.922 2.408 79.180 1.00 0.25 N
ATOM 202 CA PHE A 35 55.290 1.359 78.371 1.00 0.25 C
ATOM 203 C PHE A 35 54.085 0.746 79.061 1.00 0.25 C
ATOM 204 O PHE A 35 53.243 1.456 79.571 1.00 0.25 O
ATOM 205 CB PHE A 35 56.221 0.305 77.694 1.00 0.25 C
ATOM 206 CG PHE A 35 57.114 0.927 76.652 1.00 0.25 C
ATOM 207 CD1 PHE A 35 56.986 2.245 76.161 1.00 0.25
C
ATOM 208 CD2 PHE A 35 58.178 0.143 76.189 1.00 0.25
C
ATOM 209 CE1 PHE A 35 57.917 2.763 75.259 1.00 0.25
C
ATOM 210 CE2 PHE A 35 59.089 0.645 75.254 1.00 0.25
C
ATOM 211 CZ PHE A 35 58.958 1.959 74.789 1.00 0.25 C
ATOM 212 N THR A 36 53.961 -0.604 79.046 1.00 0.21 N
ATOM 213 CA THR A 36 52.921 -1.440 79.663 1.00 0.21
C
ATOM 214 C THR A 36 52.868 -1.382 81.177 1.00 0.21 C
ATOM 215 O THR A 36 52.013 -1.997 81.808 1.00 0.21 O
ATOM 216 CB THR A 36 53.069 -2.911 79.255 1.00 0.21
C
ATOM 217 OG1 THR A 36 54.395 -3.383 79.444 1.00 0.21
O
ATOM 218 CG2 THR A 36 52.802 -3.043 77.751 1.00 0.21
C
ATOM 219 N ALA A 37 53.777 -0.617 81.795 1.00 0.36 N

ATOM 220 CA ALA A 37 53.704 -0.221 83.167 1.00 0.36
C
ATOM 221 C ALA A 37 52.986 1.096 83.307 1.00 0.36 C
ATOM 222 O ALA A 37 52.620 1.765 82.354 1.00 0.36 O
ATOM 223 CB ALA A 37 55.123 -0.125 83.720 1.00 0.36
C
ATOM 224 N ALA A 38 52.715 1.500 84.545 1.00 0.50 N
ATOM 225 CA ALA A 38 51.921 2.660 84.722 1.00 0.50
C
ATOM 226 C ALA A 38 52.259 3.201 86.059 1.00 0.50 C
ATOM 227 O ALA A 38 52.957 2.589 86.875 1.00 0.50 O
ATOM 228 CB ALA A 38 50.416 2.311 84.676 1.00 0.50
C
ATOM 229 N GLY A 39 51.739 4.395 86.287 1.00 0.64 N
ATOM 230 CA GLY A 39 51.710 4.992 87.571 1.00 0.64
C
ATOM 231 C GLY A 39 50.335 5.278 87.915 1.00 0.64 C
ATOM 232 O GLY A 39 49.408 5.155 87.119 1.00 0.64 O
ATOM 233 N GLY A 40 50.209 5.718 89.153 1.00 0.72 N
ATOM 234 CA GLY A 40 48.942 6.037 89.715 1.00 0.72
C
ATOM 235 C GLY A 40 48.622 7.485 89.613 1.00 0.72 C
ATOM 236 O GLY A 40 49.307 8.288 88.987 1.00 0.72 O
ATOM 237 N ILE A 41 47.551 7.857 90.317 1.00 0.67 N
ATOM 238 CA ILE A 41 47.192 9.230 90.563 1.00 0.67 C
ATOM 239 C ILE A 41 48.234 9.849 91.503 1.00 0.67 C
ATOM 240 O ILE A 41 48.843 9.161 92.311 1.00 0.67 O
ATOM 241 CB ILE A 41 45.745 9.322 91.060 1.00 0.67 C
ATOM 242 CG1 ILE A 41 44.810 8.828 89.929 1.00 0.67 C
ATOM 243 CG2 ILE A 41 45.391 10.775 91.439 1.00 0.67
C
ATOM 244 CD1 ILE A 41 43.339 8.759 90.353 1.00 0.67 C
ATOM 245 N MET A 42 48.473 11.172 91.443 1.00 0.71
N
ATOM 246 CA MET A 42 49.275 11.904 92.403 1.00 0.71
C
ATOM 247 C MET A 42 48.883 11.688 93.884 1.00 0.71 C
ATOM 248 O MET A 42 49.724 11.425 94.716 1.00 0.71
O
ATOM 249 CB MET A 42 49.135 13.396 92.040 1.00 0.71
C
ATOM 250 CG MET A 42 49.907 14.344 92.970 1.00 0.71
C
ATOM 251 SD MET A 42 49.661 16.101 92.604 1.00 0.71
S
ATOM 252 CE MET A 42 47.930 16.196 93.153 1.00 0.71
C
ATOM 253 N ALA A 43 47.564 11.737 94.223 1.00 0.82 N
ATOM 254 CA ALA A 43 47.029 11.370 95.529 1.00 0.82
C
ATOM 255 C ALA A 43 47.302 9.913 95.938 1.00 0.82 C
ATOM 256 O ALA A 43 47.588 9.623 97.091 1.00 0.82 O
ATOM 257 CB ALA A 43 45.497 11.611 95.583 1.00 0.82
C
ATOM 258 N LEU A 44 47.211 8.963 94.974 1.00 0.76 N
ATOM 259 CA LEU A 44 47.524 7.554 95.170 1.00 0.76 C
ATOM 260 C LEU A 44 48.997 7.314 95.501 1.00 0.76 C
ATOM 261 O LEU A 44 49.331 6.633 96.475 1.00 0.76 O
ATOM 262 CB LEU A 44 47.138 6.783 93.873 1.00 0.76 C
ATOM 263 CG LEU A 44 47.407 5.265 93.878 1.00 0.76 C
ATOM 264 CD1 LEU A 44 46.654 4.564 95.009 1.00 0.76
C
ATOM 265 CD2 LEU A 44 46.993 4.628 92.544 1.00 0.76
C
ATOM 266 N ILE A 45 49.915 7.935 94.732 1.00 0.74 N
ATOM 267 CA ILE A 45 51.360 7.914 94.935 1.00 0.74 C
ATOM 268 C ILE A 45 51.768 8.583 96.231 1.00 0.74 C
ATOM 269 O ILE A 45 52.615 8.084 96.972 1.00 0.74 O
ATOM 270 CB ILE A 45 52.083 8.506 93.731 1.00 0.74 C
ATOM 271 CG1 ILE A 45 52.000 7.467 92.592 1.00 0.74 C
ATOM 272 CG2 ILE A 45 53.559 8.820 94.065 1.00 0.74 C
ATOM 273 CD1 ILE A 45 52.629 7.954 91.285 1.00 0.74 C
ATOM 274 N LYS A 46 51.131 9.718 96.567 1.00 0.77 N
ATOM 275 CA LYS A 46 51.326 10.393 97.833 1.00 0.77
C
ATOM 276 C LYS A 46 50.996 9.538 99.045 1.00 0.77 C
ATOM 277 O LYS A 46 51.747 9.511 100.016 1.00 0.77 O
ATOM 278 CB LYS A 46 50.412 11.641 97.924 1.00 0.77
C
ATOM 279 CG LYS A 46 51.017 12.911 97.307 1.00 0.77
C
ATOM 280 CD LYS A 46 50.327 14.217 97.745 1.00 0.77
C
ATOM 281 CE LYS A 46 50.396 14.475 99.260 1.00 0.77
C
ATOM 282 NZ LYS A 46 49.375 15.460 99.678 1.00 0.77
N
ATOM 283 N LYS A 47 49.863 8.817 99.003 1.00 0.78 N
ATOM 284 CA LYS A 47 49.424 7.889 100.025 1.00 0.78
C
ATOM 285 C LYS A 47 50.364 6.719 100.194 1.00 0.78 C
ATOM 286 O LYS A 47 50.646 6.253 101.303 1.00 0.78 O
ATOM 287 CB LYS A 47 48.025 7.392 99.620 1.00 0.78 C
ATOM 288 CG LYS A 47 47.292 6.576 100.700 1.00 0.78
C
ATOM 289 CD LYS A 47 45.809 6.313 100.364 1.00 0.78
C
ATOM 290 CE LYS A 47 45.063 7.584 99.932 1.00 0.78 C
ATOM 291 NZ LYS A 47 43.647 7.300 99.631 1.00 0.78 N
ATOM 292 N TYR A 48 50.913 6.219 99.080 1.00 0.71 N
ATOM 293 CA TYR A 48 51.967 5.235 99.110 1.00 0.71
C
ATOM 294 C TYR A 48 53.268 5.708 99.736 1.00 0.71 C
ATOM 295 O TYR A 48 53.894 4.956 100.480 1.00 0.71 O
ATOM 296 CB TYR A 48 52.280 4.683 97.705 1.00 0.71 C
ATOM 297 CG TYR A 48 51.162 3.906 97.072 1.00 0.71
C
ATOM 298 CD1 TYR A 48 50.267 3.135 97.835 1.00 0.71
C
ATOM 299 CD2 TYR A 48 51.070 3.857 95.669 1.00 0.71
C
ATOM 300 CE1 TYR A 48 49.307 2.335 97.211 1.00 0.71
C
ATOM 301 CE2 TYR A 48 50.112 3.044 95.043 1.00 0.71
C
ATOM 302 CZ TYR A 48 49.242 2.275 95.823 1.00 0.71 C
ATOM 303 OH TYR A 48 48.290 1.419 95.244 1.00 0.71
O
ATOM 304 N ALA A 49 53.698 6.960 99.478 1.00 0.74 N
ATOM 305 CA ALA A 49 54.813 7.575 100.167 1.00 0.74
C
ATOM 306 C ALA A 49 54.588 7.752 101.667 1.00 0.74 C
ATOM 307 O ALA A 49 55.498 7.505 102.459 1.00 0.74 O
ATOM 308 CB ALA A 49 55.111 8.954 99.551 1.00 0.74
C
ATOM 309 N GLN A 50 53.368 8.155 102.090 1.00 0.67 N
ATOM 310 CA GLN A 50 52.955 8.253 103.485 1.00 0.67
C
ATOM 311 C GLN A 50 52.993 6.924 104.220 1.00 0.67 C
ATOM 312 O GLN A 50 53.411 6.852 105.371 1.00 0.67 O
ATOM 313 CB GLN A 50 51.517 8.825 103.606 1.00 0.67
C
ATOM 314 CG GLN A 50 51.437 10.315 103.211 1.00 0.67
C
ATOM 315 CD GLN A 50 49.998 10.825 103.193 1.00 0.67
C
ATOM 316 OE1 GLN A 50 49.017 10.112 103.002 1.00 0.67
O

ATOM 317 NE2 GLN A 50 49.853 12.159 103.389 1.00 0.67
N
ATOM 318 N LYS A 51 52.542 5.838 103.561 1.00 0.68 N
ATOM 319 CA LYS A 51 52.606 4.493 104.088 1.00 0.68
C
ATOM 320 C LYS A 51 54.006 3.883 104.168 1.00 0.68 C
ATOM 321 O LYS A 51 54.330 3.197 105.126 1.00 0.68 O
ATOM 322 CB LYS A 51 51.735 3.565 103.203 1.00 0.68
C
ATOM 323 CG LYS A 51 51.679 2.097 103.667 1.00 0.68
C
ATOM 324 CD LYS A 51 50.889 1.227 102.681 1.00 0.68
C
ATOM 325 CE LYS A 51 51.035 -0.274 102.952 1.00 0.68
C
ATOM 326 NZ LYS A 51 50.202 -1.048 102.002 1.00 0.68
N
ATOM 327 N LYS A 52 54.840 4.051 103.111 1.00 0.59 N
ATOM 328 CA LYS A 52 56.038 3.237 102.967 1.00 0.59
C
ATOM 329 C LYS A 52 57.366 3.966 102.913 1.00 0.59 C
ATOM 330 O LYS A 52 58.401 3.331 102.983 1.00 0.59 O
ATOM 331 CB LYS A 52 56.009 2.502 101.618 1.00 0.59
C
ATOM 332 CG LYS A 52 54.809 1.581 101.437 1.00 0.59
C
ATOM 333 CD LYS A 52 54.988 0.831 100.116 1.00 0.59
C
ATOM 334 CE LYS A 52 53.793 -0.033 99.745 1.00 0.59 C
ATOM 335 NZ LYS A 52 54.044 -0.692 98.445 1.00 0.59 N
ATOM 336 N LEU A 53 57.356 5.309 102.747 1.00 0.58 N
ATOM 337 CA LEU A 53 58.532 6.103 102.428 1.00 0.58
C
ATOM 338 C LEU A 53 58.871 6.111 100.946 1.00 0.58 C
ATOM 339 O LEU A 53 58.518 5.212 100.169 1.00 0.58 O
ATOM 340 CB LEU A 53 59.812 5.828 103.272 1.00 0.58
C
ATOM 341 CG LEU A 53 59.555 5.813 104.787 1.00 0.58
C
ATOM 342 CD1 LEU A 53 60.669 5.035 105.502 1.00 0.58
C
ATOM 343 CD2 LEU A 53 59.383 7.241 105.320 1.00 0.58
C
ATOM 344 N TRP A 54 59.570 7.164 100.485 1.00 0.53 N
ATOM 345 CA TRP A 54 59.947 7.353 99.094 1.00 0.53 C
ATOM 346 C TRP A 54 60.831 6.265 98.513 1.00 0.53 C
ATOM 347 O TRP A 54 60.608 5.822 97.392 1.00 0.53 O
ATOM 348 CB TRP A 54 60.641 8.721 98.895 1.00 0.53 C
ATOM 349 CG TRP A 54 59.720 9.911 99.042 1.00 0.53 C
ATOM 350 CD1 TRP A 54 58.364 9.981 98.895 1.00 0.53
C
ATOM 351 CD2 TRP A 54 60.166 11.251 99.300 1.00 0.53
C
ATOM 352 NE1 TRP A 54 57.923 11.273 99.083 1.00 0.53
N
ATOM 353 CE2 TRP A 54 59.023 12.068 99.323 1.00 0.53
C
ATOM 354 CE3 TRP A 54 61.440 11.776 99.494 1.00 0.53
C
ATOM 355 CZ2 TRP A 54 59.127 13.435 99.550 1.00 0.53
C
ATOM 356 CZ3 TRP A 54 61.547 13.156 99.721 1.00 0.53
C
ATOM 357 CH2 TRP A 54 60.408 13.974 99.750 1.00 0.53
C
ATOM 358 N LYS A 55 61.835 5.777 99.272 1.00 0.57 N
ATOM 359 CA LYS A 55 62.707 4.699 98.836 1.00 0.57 C
ATOM 360 C LYS A 55 61.979 3.386 98.559 1.00 0.57 C
ATOM 361 O LYS A 55 62.249 2.718 97.569 1.00 0.57 O
ATOM 362 CB LYS A 55 63.810 4.428 99.894 1.00 0.57 C
ATOM 363 CG LYS A 55 64.816 3.346 99.456 1.00 0.57 C
ATOM 364 CD LYS A 55 65.917 3.102 100.499 1.00 0.57
C
ATOM 365 CE LYS A 55 66.899 1.998 100.082 1.00 0.57
C
ATOM 366 NZ LYS A 55 67.942 1.808 101.119 1.00 0.57
N
ATOM 367 N GLN A 56 61.032 2.992 99.441 1.00 0.54 N
ATOM 368 CA GLN A 56 60.242 1.786 99.268 1.00 0.54
C
ATOM 369 C GLN A 56 59.147 1.922 98.234 1.00 0.54 C
ATOM 370 O GLN A 56 58.703 0.930 97.674 1.00 0.54 O
ATOM 371 CB GLN A 56 59.550 1.390 100.587 1.00 0.54
C
ATOM 372 CG GLN A 56 60.522 0.897 101.674 1.00 0.54
C
ATOM 373 CD GLN A 56 59.754 0.433 102.914 1.00 0.54
C
ATOM 374 OE1 GLN A 56 58.540 0.264 102.932 1.00 0.54
O
ATOM 375 NE2 GLN A 56 60.521 0.162 103.997 1.00 0.54
N
ATOM 376 N LEU A 57 58.656 3.146 97.965 1.00 0.60 N
ATOM 377 CA LEU A 57 57.790 3.388 96.835 1.00 0.60 C
ATOM 378 C LEU A 57 58.458 3.335 95.451 1.00 0.60 C
ATOM 379 O LEU A 57 57.852 2.946 94.470 1.00 0.60 O
ATOM 380 CB LEU A 57 57.112 4.752 97.000 1.00 0.60 C
ATOM 381 CG LEU A 57 56.175 5.076 95.832 1.00 0.60 C
ATOM 382 CD1 LEU A 57 55.100 4.006 95.583 1.00 0.60
C
ATOM 383 CD2 LEU A 57 55.490 6.397 96.091 1.00 0.60
C
ATOM 384 N ILE A 58 59.721 3.806 95.345 1.00 0.62 N
ATOM 385 CA ILE A 58 60.557 3.620 94.160 1.00 0.62 C
ATOM 386 C ILE A 58 60.890 2.152 93.878 1.00 0.62 C
ATOM 387 O ILE A 58 60.998 1.754 92.726 1.00 0.62 O
ATOM 388 CB ILE A 58 61.857 4.426 94.277 1.00 0.62 C
ATOM 389 CG1 ILE A 58 61.549 5.943 94.235 1.00 0.62 C
ATOM 390 CG2 ILE A 58 62.871 4.041 93.163 1.00 0.62 C
ATOM 391 CD1 ILE A 58 62.751 6.802 94.657 1.00 0.62 C
ATOM 392 N ALA A 59 61.122 1.367 94.954 1.00 0.66 N
ATOM 393 CA ALA A 59 61.358 -0.061 94.896 1.00 0.66
C
ATOM 394 C ALA A 59 60.155 -0.963 94.467 1.00 0.66 C
ATOM 395 O ALA A 59 59.002 -0.484 94.308 1.00 0.66 O
ATOM 396 CB ALA A 59 61.852 -0.531 96.286 1.00 0.66
C
ATOM 397 OXT ALA A 59 60.413 -2.189 94.290 1.00 0.66
O
TER 398 ALA A 59
END

B)

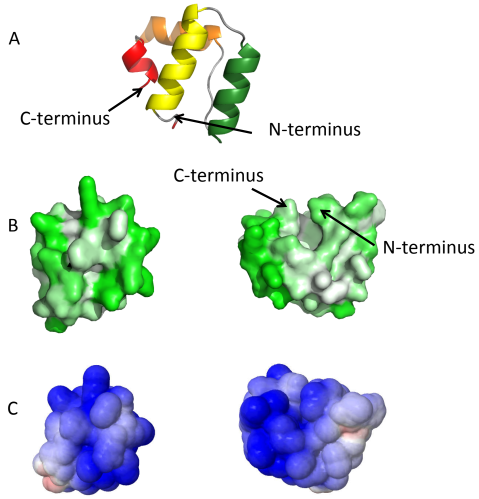

Figure S5- Structural models of garvacin ML. A) Ribbon diagram depicting the saposin-like fold. The C- and N- termini are explicitly shown. Hydrophobic surface (B) and electrostatic potential surface (C) maps of garvacin ML. Orientation peptide backbone in surface filling diagrams, B and C, correlate to one another.

6.Mutacin BhtB

A) PDB file for model of Mutacin BhtB. Structure modeled after lacticin Q using SWISS-MODEL[84]

```
TITLE     SWISS-MODEL SERVER (https://swissmodel.expasy.org)
TITLE    2 Untitled Project
EXPDTA    THEORETICAL MODEL (SWISS-MODEL SERVER)
AUTHOR    SWISS-MODEL SERVER (SEE REFERENCE IN JRNL
Records)
REVDAT   1   15-AUG-16 1MOD    1       21:39
JRNL        AUTH
M.BIASINI,S.BIENERT,A.WATERHOUSE,K.ARNOLD,G.STUDER,
JRNL        AUTH 2 T.SCHMIDT,F.KIEFER,T.GALLO
CASSARINO,M.BERTONI,L.BORDOLI,
JRNL        AUTH 3 T.SCHWEDE
JRNL        TITL   SWISS-MODEL: MODELLING PROTEIN TERTIARY
AND QUATERNARY
JRNL        TITL 2 STRUCTURE USING EVOLUTIONARY
INFORMATION
JRNL        REF    NUCLEIC.ACIDS.RES                       2014
JRNL        REFN                 ISSN 0305-1048
JRNL        PMID   24782522
JRNL        DOI    10.1093/nar/gku340
REMARK   3
REMARK   3 MODEL INFORMATION
REMARK   3  ENGIN   PROMOD3
REMARK   3  VERSN   1.0.0
REMARK   3  OSTAT   MONOMER
REMARK   3  OSRSN   USER
REMARK   3  GMQE    0.74
REMARK   3  QMN4    -1.71
REMARK   3
REMARK   3 TEMPLATE 1
REMARK   3  PDBID   2n8p
REMARK   3  CHAIN   A
REMARK   3  MMCIF   A
REMARK   3  PDBV    2016-08-12
REMARK   3  SMTLE   2n8p.1.A
REMARK   3  SMTLV   2016-08-14
REMARK   3  MTHD    SOLUTION NMR 0.00 A
REMARK   3  FOUND   HHblits
REMARK   3  GMQE    0.74
REMARK   3  SIM     0.43
REMARK   3  SID     45.24
REMARK   3  OSTAT   monomer
ATOM      1  N   TRP A   2       9.109  -0.818  -0.130  1.00  0.48           N
ATOM      2  CA  TRP A   2      10.436  -1.289  -0.665  1.00  0.48           C
ATOM      3  C   TRP A   2      10.867  -0.538  -1.927  1.00  0.48           C
ATOM      4  O   TRP A   2      11.803   0.265  -1.816  1.00  0.48           O
ATOM      5  CB  TRP A   2      10.478  -2.840  -0.737  1.00  0.48           C
ATOM      6  CG  TRP A   2      11.868  -3.431  -0.982  1.00  0.48           C
ATOM      7  CD1 TRP A   2      13.012  -3.412  -0.217  1.00  0.48           C
ATOM      8  CD2 TRP A   2      12.196  -4.175  -2.166  1.00  0.48           C
ATOM      9  NE1 TRP A   2      14.021  -4.105  -0.853  1.00  0.48           N
ATOM     10  CE2 TRP A   2      13.539  -4.578  -2.042  1.00  0.48           C
ATOM     11  CE3 TRP A   2      11.445  -4.512  -3.289  1.00  0.48           C
ATOM     12  CZ2 TRP A   2      14.154  -5.326  -3.029  1.00  0.48           C
ATOM     13  CZ3 TRP A   2      12.069  -5.276  -4.285  1.00  0.48           C
ATOM     14  CH2 TRP A   2      13.401  -5.686  -4.152  1.00  0.48           C
ATOM     15  N   GLY A   3      10.233  -0.618  -3.127  1.00  0.61           N
ATOM     16  CA  GLY A   3      10.771   0.129  -4.291  1.00  0.61           C
ATOM     17  C   GLY A   3      10.911   1.649  -4.148  1.00  0.61           C
```

ATOM	18	O	GLY	A	3	11.784	2.266	-4.752	1.00	0.61	O
ATOM	19	N	ARG	A	4	10.071	2.287	-3.310	1.00	0.50	N
ATOM	20	CA	ARG	A	4	10.164	3.700	-2.950	1.00	0.50	C
ATOM	21	C	ARG	A	4	11.385	4.114	-2.117	1.00	0.50	C
ATOM	22	O	ARG	A	4	12.016	5.130	-2.400	1.00	0.50	O
ATOM	23	CB	ARG	A	4	8.886	4.147	-2.206	1.00	0.50	C
ATOM	24	CG	ARG	A	4	7.603	4.020	-3.050	1.00	0.50	C
ATOM	25	CD	ARG	A	4	6.366	4.465	-2.267	1.00	0.50	C
ATOM	26	NE	ARG	A	4	5.188	4.302	-3.177	1.00	0.50	N
ATOM	27	CZ	ARG	A	4	3.915	4.473	-2.793	1.00	0.50	C
ATOM	28	NH1	ARG	A	4	2.934	4.351	-3.685	1.00	0.50	N
ATOM	29	NH2	ARG	A	4	3.595	4.782	-1.540	1.00	0.50	N
ATOM	30	N	ILE	A	5	11.775	3.335	-1.078	1.00	0.51	N
ATOM	31	CA	ILE	A	5	12.965	3.591	-0.259	1.00	0.51	C
ATOM	32	C	ILE	A	5	14.237	3.498	-1.117	1.00	0.51	C
ATOM	33	O	ILE	A	5	15.189	4.260	-0.935	1.00	0.51	O
ATOM	34	CB	ILE	A	5	13.024	2.749	1.041	1.00	0.51	C
ATOM	35	CG1	ILE	A	5	13.102	1.232	0.764	1.00	0.51	C
ATOM	36	CG2	ILE	A	5	11.783	3.082	1.903	1.00	0.51	C
ATOM	37	CD1	ILE	A	5	13.354	0.308	1.964	1.00	0.51	C
ATOM	38	N	LEU	A	6	14.247	2.584	-2.117	1.00	0.57	N
ATOM	39	CA	LEU	A	6	15.290	2.390	-3.126	1.00	0.57	C
ATOM	40	C	LEU	A	6	15.480	3.511	-4.097	1.00	0.57	C
ATOM	41	O	LEU	A	6	16.590	3.791	-4.550	1.00	0.57	O
ATOM	42	CB	LEU	A	6	15.022	1.125	-3.979	1.00	0.57	C
ATOM	43	CG	LEU	A	6	14.952	-0.125	-3.084	1.00	0.57	C
ATOM	44	CD1	LEU	A	6	14.763	-1.459	-3.808	1.00	0.57	C
ATOM	45	CD2	LEU	A	6	16.263	-0.231	-2.295	1.00	0.57	C
ATOM	46	N	ALA	A	7	14.371	4.158	-4.468	1.00	0.62	N
ATOM	47	CA	ALA	A	7	14.361	5.222	-5.432	1.00	0.62	C
ATOM	48	C	ALA	A	7	15.186	6.408	-4.954	1.00	0.62	C
ATOM	49	O	ALA	A	7	15.955	7.003	-5.708	1.00	0.62	O
ATOM	50	CB	ALA	A	7	12.900	5.608	-5.715	1.00	0.62	C
ATOM	51	N	PHE	A	8	15.104	6.727	-3.649	1.00	0.47	N
ATOM	52	CA	PHE	A	8	15.924	7.737	-3.007	1.00	0.47	C
ATOM	53	C	PHE	A	8	17.401	7.441	-3.022	1.00	0.47	C
ATOM	54	O	PHE	A	8	18.222	8.349	-3.121	1.00	0.47	O
ATOM	55	CB	PHE	A	8	15.532	7.923	-1.532	1.00	0.47	C
ATOM	56	CG	PHE	A	8	14.181	8.544	-1.440	1.00	0.47	C
ATOM	57	CD1	PHE	A	8	13.147	7.912	-0.736	1.00	0.47	C
ATOM	58	CD2	PHE	A	8	13.943	9.787	-2.045	1.00	0.47	C
ATOM	59	CE1	PHE	A	8	11.892	8.522	-0.627	1.00	0.47	C
ATOM	60	CE2	PHE	A	8	12.684	10.387	-1.960	1.00	0.47	C
ATOM	61	CZ	PHE	A	8	11.658	9.759	-1.243	1.00	0.47	C
ATOM	62	N	VAL	A	9	17.787	6.161	-2.926	1.00	0.56	N
ATOM	63	CA	VAL	A	9	19.167	5.734	-2.966	1.00	0.56	C
ATOM	64	C	VAL	A	9	19.878	6.103	-4.274	1.00	0.56	C
ATOM	65	O	VAL	A	9	21.082	6.353	-4.276	1.00	0.56	O
ATOM	66	CB	VAL	A	9	19.328	4.249	-2.672	1.00	0.56	C
ATOM	67	CG1	VAL	A	9	20.830	3.966	-2.514	1.00	0.56	C
ATOM	68	CG2	VAL	A	9	18.719	3.841	-1.318	1.00	0.56	C
ATOM	69	N	ALA	A	10	19.143	6.240	-5.403	1.00	0.54	N
ATOM	70	CA	ALA	A	10	19.645	6.697	-6.693	1.00	0.54	C
ATOM	71	C	ALA	A	10	20.451	8.006	-6.636	1.00	0.54	C
ATOM	72	O	ALA	A	10	21.399	8.203	-7.398	1.00	0.54	O
ATOM	73	CB	ALA	A	10	18.434	6.861	-7.638	1.00	0.54	C
ATOM	74	N	LYS	A	11	20.115	8.909	-5.685	1.00	0.44	N
ATOM	75	CA	LYS	A	11	20.882	10.098	-5.339	1.00	0.44	C
ATOM	76	C	LYS	A	11	22.294	9.832	-4.785	1.00	0.44	C
ATOM	77	O	LYS	A	11	23.212	10.621	-5.011	1.00	0.44	O
ATOM	78	CB	LYS	A	11	20.067	11.015	-4.365	1.00	0.44	C
ATOM	79	CG	LYS	A	11	20.105	10.641	-2.865	1.00	0.44	C
ATOM	80	CD	LYS	A	11	21.217	11.330	-2.061	1.00	0.44	C
ATOM	81	CE	LYS	A	11	20.772	12.643	-1.427	1.00	0.44	C
ATOM	82	NZ	LYS	A	11	21.970	13.473	-1.207	1.00	0.44	N
ATOM	83	N	TYR	A	12	22.475	8.751	-3.984	1.00	0.39	N
ATOM	84	CA	TYR	A	12	23.710	8.330	-3.324	1.00	0.39	C
ATOM	85	C	TYR	A	12	24.642	7.672	-4.320	1.00	0.39	C
ATOM	86	O	TYR	A	12	25.863	7.790	-4.232	1.00	0.39	O
ATOM	87	CB	TYR	A	12	23.455	7.337	-2.141	1.00	0.39	C
ATOM	88	CG	TYR	A	12	23.015	8.036	-0.877	1.00	0.39	C
ATOM	89	CD1	TYR	A	12	23.974	8.677	-0.076	1.00	0.39	C
ATOM	90	CD2	TYR	A	12	21.682	8.004	-0.430	1.00	0.39	C
ATOM	91	CE1	TYR	A	12	23.611	9.291	1.134	1.00	0.39	C
ATOM	92	CE2	TYR	A	12	21.317	8.613	0.780	1.00	0.39	C
ATOM	93	CZ	TYR	A	12	22.278	9.261	1.563	1.00	0.39	C
ATOM	94	OH	TYR	A	12	21.865	9.852	2.781	1.00	0.39	O
ATOM	95	N	GLY	A	13	24.070	6.953	-5.300	1.00	0.49	N
ATOM	96	CA	GLY	A	13	24.817	6.346	-6.382	1.00	0.49	C
ATOM	97	C	GLY	A	13	24.294	4.964	-6.655	1.00	0.49	C
ATOM	98	O	GLY	A	13	23.115	4.661	-6.475	1.00	0.49	O
ATOM	99	N	THR	A	14	25.186	4.062	-7.099	1.00	0.50	N
ATOM	100	CA	THR	A	14	24.845	2.709	-7.524	1.00	0.50	C
ATOM	101	C	THR	A	14	25.397	1.656	-6.578	1.00	0.50	C
ATOM	102	O	THR	A	14	25.439	0.466	-6.893	1.00	0.50	O
ATOM	103	CB	THR	A	14	25.283	2.416	-8.952	1.00	0.50	C
ATOM	104	OG1	THR	A	14	26.642	2.772	-9.177	1.00	0.50	O
ATOM	105	CG2	THR	A	14	24.415	3.294	-9.866	1.00	0.50	C
ATOM	106	N	LYS	A	15	25.802	2.054	-5.351	1.00	0.52	N
ATOM	107	CA	LYS	A	15	26.430	1.147	-4.401	1.00	0.52	C
ATOM	108	C	LYS	A	15	25.547	0.831	-3.208	1.00	0.52	C
ATOM	109	O	LYS	A	15	25.326	-0.330	-2.869	1.00	0.52	O
ATOM	110	CB	LYS	A	15	27.757	1.772	-3.925	1.00	0.52	C
ATOM	111	CG	LYS	A	15	28.673	0.818	-3.142	1.00	0.52	C
ATOM	112	CD	LYS	A	15	30.101	1.388	-3.047	1.00	0.52	C
ATOM	113	CE	LYS	A	15	30.957	1.247	-4.315	1.00	0.52	C
ATOM	114	NZ	LYS	A	15	31.339	-0.168	-4.519	1.00	0.52	N
ATOM	115	N	ALA	A	16	24.944	1.854	-2.573	1.00	0.63	N
ATOM	116	CA	ALA	A	16	24.040	1.666	-1.459	1.00	0.63	C
ATOM	117	C	ALA	A	16	22.711	1.028	-1.901	1.00	0.63	C
ATOM	118	O	ALA	A	16	22.036	0.348	-1.126	1.00	0.63	O
ATOM	119	CB	ALA	A	16	23.857	3.039	-0.772	1.00	0.63	C
ATOM	120	N	VAL	A	17	22.329	1.203	-3.195	1.00	0.61	N
ATOM	121	CA	VAL	A	17	21.102	0.706	-3.818	1.00	0.61	C
ATOM	122	C	VAL	A	17	21.126	-0.804	-3.874	1.00	0.61	C
ATOM	123	O	VAL	A	17	20.144	-1.495	-3.600	1.00	0.61	O
ATOM	124	CB	VAL	A	17	20.829	1.371	-5.185	1.00	0.61	C
ATOM	125	CG1	VAL	A	17	21.903	1.094	-6.259	1.00	0.61	C
ATOM	126	CG2	VAL	A	17	19.402	1.073	-5.697	1.00	0.61	C
ATOM	127	N	GLN	A	18	22.321	-1.352	-4.159	1.00	0.62	N
ATOM	128	CA	GLN	A	18	22.577	-2.764	-4.288	1.00	0.62	C
ATOM	129	C	GLN	A	18	22.314	-3.540	-3.009	1.00	0.62	C
ATOM	130	O	GLN	A	18	21.824	-4.672	-3.036	1.00	0.62	O
ATOM	131	CB	GLN	A	18	24.020	-2.980	-4.779	1.00	0.62	C
ATOM	132	CG	GLN	A	18	24.221	-4.434	-5.239	1.00	0.62	C
ATOM	133	CD	GLN	A	18	25.457	-4.562	-6.112	1.00	0.62	C
ATOM	134	OE1	GLN	A	18	26.551	-4.904	-5.647	1.00	0.62	O
ATOM	135	NE2	GLN	A	18	25.292	-4.273	-7.419	1.00	0.62	N
ATOM	136	N	TRP	A	19	22.611	-2.933	-1.846	1.00	0.52	N
ATOM	137	CA	TRP	A	19	22.476	-3.547	-0.537	1.00	0.52	C
ATOM	138	C	TRP	A	19	21.049	-3.965	-0.218	1.00	0.52	C
ATOM	139	O	TRP	A	19	20.787	-5.048	0.341	1.00	0.52	O
ATOM	140	CB	TRP	A	19	22.911	-2.541	0.563	1.00	0.52	C
ATOM	141	CG	TRP	A	19	22.915	-3.200	1.929	1.00	0.52	C
ATOM	142	CD1	TRP	A	19	23.664	-4.280	2.283	1.00	0.52	C
ATOM	143	CD2	TRP	A	19	22.000	-2.969	3.019	1.00	0.52	C
ATOM	144	NE1	TRP	A	19	23.296	-4.742	3.524	1.00	0.52	N
ATOM	145	CE2	TRP	A	19	22.260	-3.960	3.987	1.00	0.52	C
ATOM	146	CE3	TRP	A	19	21.014	-2.006	3.224	1.00	0.52	C
ATOM	147	CZ2	TRP	A	19	21.567	-3.992	5.192	1.00	0.52	C
ATOM	148	CZ3	TRP	A	19	20.420	-1.957	4.494	1.00	0.52	C
ATOM	149	CH2	TRP	A	19	20.698	-2.928	5.466	1.00	0.52	C

```
ATOM    150  N   ALA A  20      20.109  -3.072  -0.540  1.00  0.66           N
ATOM    151  CA  ALA A  20      18.705  -3.170  -0.240  1.00  0.66           C
ATOM    152  C   ALA A  20      17.903  -3.900  -1.317  1.00  0.66           C
ATOM    153  O   ALA A  20      16.890  -4.535  -0.995  1.00  0.66           O
ATOM    154  CB  ALA A  20      18.278  -1.686   0.002  1.00  0.66           C
ATOM    155  N   TRP A  21      18.366  -3.900  -2.589  1.00  0.54           N
ATOM    156  CA  TRP A  21      17.972  -4.869  -3.615  1.00  0.54           C
ATOM    157  C   TRP A  21      18.362  -6.303  -3.281  1.00  0.54           C
ATOM    158  O   TRP A  21      17.581  -7.237  -3.471  1.00  0.54           O
ATOM    159  CB  TRP A  21      18.550  -4.554  -5.021  1.00  0.54           C
ATOM    160  CG  TRP A  21      17.697  -3.608  -5.834  1.00  0.54           C
ATOM    161  CD1 TRP A  21      17.895  -2.281  -6.032  1.00  0.54
C
ATOM    162  CD2 TRP A  21      16.498  -3.948  -6.558  1.00  0.54
C
ATOM    163  NE1 TRP A  21      16.907  -1.749  -6.835  1.00  0.54
N
ATOM    164  CE2 TRP A  21      16.031  -2.767  -7.162  1.00  0.54           C
ATOM    165  CE3 TRP A  21      15.822  -5.154  -6.713  1.00  0.54           C
ATOM    166  CZ2 TRP A  21      14.867  -2.764  -7.930  1.00  0.54           C
ATOM    167  CZ3 TRP A  21      14.655  -5.155  -7.490  1.00  0.54           C
ATOM    168  CH2 TRP A  21      14.181  -3.981  -8.088  1.00  0.54
C
ATOM    169  N   LYS A  22      19.599  -6.497  -2.781  1.00  0.65           N
ATOM    170  CA  LYS A  22      20.102  -7.765  -2.290  1.00  0.65           C
ATOM    171  C   LYS A  22      19.352  -8.296  -1.063  1.00  0.65           C
ATOM    172  O   LYS A  22      18.976  -9.467  -1.000  1.00  0.65           O
ATOM    173  CB  LYS A  22      21.606  -7.600  -1.946  1.00  0.65           C
ATOM    174  CG  LYS A  22      22.293  -8.893  -1.493  1.00  0.65           C
ATOM    175  CD  LYS A  22      23.788  -8.701  -1.188  1.00  0.65           C
ATOM    176  CE  LYS A  22      24.453  -9.998  -0.716  1.00  0.65           C
ATOM    177  NZ  LYS A  22      25.895  -9.790  -0.453  1.00  0.65           N
ATOM    178  N   ASN A  23      19.098  -7.430  -0.058  1.00  0.64           N
ATOM    179  CA  ASN A  23      18.428  -7.784   1.185  1.00  0.64           C
ATOM    180  C   ASN A  23      16.974  -7.378   1.185  1.00  0.64           C
ATOM    181  O   ASN A  23      16.552  -6.421   1.838  1.00  0.64           O
ATOM    182  CB  ASN A  23      19.086  -7.145   2.422  1.00  0.64           C
ATOM    183  CG  ASN A  23      20.411  -7.840   2.638  1.00  0.64           C
ATOM    184  OD1 ASN A  23      20.442  -9.008   3.049  1.00  0.64
O
ATOM    185  ND2 ASN A  23      21.536  -7.151   2.376  1.00  0.64
N
ATOM    186  N   LYS A  24      16.153  -8.171   0.486  1.00  0.55           N
ATOM    187  CA  LYS A  24      14.736  -7.961   0.349  1.00  0.55           C
ATOM    188  C   LYS A  24      13.943  -8.840   1.299  1.00  0.55           C
ATOM    189  O   LYS A  24      12.953  -9.467   0.920  1.00  0.55           O
ATOM    190  CB  LYS A  24      14.340  -8.221  -1.116  1.00  0.55           C
ATOM    191  CG  LYS A  24      14.686  -9.615  -1.669  1.00  0.55           C
ATOM    192  CD  LYS A  24      14.249  -9.733  -3.139  1.00  0.55           C
ATOM    193  CE  LYS A  24      12.740  -9.851  -3.382  1.00  0.55           C
ATOM    194  NZ  LYS A  24      12.299 -11.222  -3.045  1.00  0.55
N
ATOM    195  N   TRP A  25      14.393  -8.941   2.565  1.00  0.37           N
ATOM    196  CA  TRP A  25      13.669  -9.684   3.576  1.00  0.37           C
ATOM    197  C   TRP A  25      13.757  -9.015   4.949  1.00  0.37           C
ATOM    198  O   TRP A  25      12.754  -8.887   5.648  1.00  0.37           O
ATOM    199  CB  TRP A  25      14.179 -11.158   3.605  1.00  0.37           C
ATOM    200  CG  TRP A  25      15.660 -11.307   3.922  1.00  0.37           C
ATOM    201  CD1 TRP A  25      16.746 -11.214   3.093  1.00  0.37
C
ATOM    202  CD2 TRP A  25      16.183 -11.441   5.255  1.00  0.37
C
ATOM    203  NE1 TRP A  25      17.914 -11.259   3.823  1.00  0.37
N
ATOM    204  CE2 TRP A  25      17.586 -11.407   5.152  1.00  0.37
C
ATOM    205  CE3 TRP A  25      15.549 -11.545   6.492  1.00  0.37
C
ATOM    206  CZ2 TRP A  25      18.387 -11.488   6.284  1.00  0.37
C
ATOM    207  CZ3 TRP A  25      16.357 -11.619   7.634  1.00  0.37
C
ATOM    208  CH2 TRP A  25      17.755 -11.591   7.533  1.00  0.37
C
ATOM    209  N   PHE A  26      14.938  -8.487   5.343  1.00  0.35           N
ATOM    210  CA  PHE A  26      15.150  -7.843   6.634  1.00  0.35           C
ATOM    211  C   PHE A  26      14.695  -6.387   6.621  1.00  0.35           C
ATOM    212  O   PHE A  26      14.257  -5.818   7.617  1.00  0.35           O
ATOM    213  CB  PHE A  26      16.659  -7.970   6.964  1.00  0.35           C
ATOM    214  CG  PHE A  26      16.961  -7.576   8.384  1.00  0.35           C
ATOM    215  CD1 PHE A  26      16.593  -8.393   9.467  1.00  0.35           C
ATOM    216  CD2 PHE A  26      17.574  -6.341   8.641  1.00  0.35           C
ATOM    217  CE1 PHE A  26      16.845  -7.981  10.784  1.00  0.35
C
ATOM    218  CE2 PHE A  26      17.812  -5.921   9.954  1.00  0.35           C
ATOM    219  CZ  PHE A  26      17.455  -6.745  11.027  1.00  0.35           C
ATOM    220  N   LEU A  27      14.732  -5.766   5.434  1.00  0.44           N
ATOM    221  CA  LEU A  27      14.284  -4.406   5.200  1.00  0.44           C
ATOM    222  C   LEU A  27      12.835  -4.377   4.798  1.00  0.44           C
ATOM    223  O   LEU A  27      12.274  -3.294   4.524  1.00  0.44           O
ATOM    224  CB  LEU A  27      15.041  -3.796   4.008  1.00  0.44           C
ATOM    225  CG  LEU A  27      16.561  -3.740   4.163  1.00  0.44           C
ATOM    226  CD1 LEU A  27      17.127  -3.231   2.840  1.00  0.44
C
ATOM    227  CD2 LEU A  27      17.001  -2.850   5.333  1.00  0.44
C
ATOM    228  N   LEU A  28      12.130  -5.510   4.760  1.00  0.39           N
ATOM    229  CA  LEU A  28      10.705  -5.479   4.560  1.00  0.39           C
ATOM    230  C   LEU A  28       9.943  -5.157   5.835  1.00  0.39           C
ATOM    231  O   LEU A  28       8.815  -4.676   5.768  1.00  0.39           O
ATOM    232  CB  LEU A  28      10.205  -6.813   4.002  1.00  0.39           C
ATOM    233  CG  LEU A  28      10.671  -7.097   2.566  1.00  0.39           C
ATOM    234  CD1 LEU A  28      10.179  -8.503   2.225  1.00  0.39
C
ATOM    235  CD2 LEU A  28      10.144  -6.080   1.535  1.00  0.39
C
ATOM    236  N   SER A  29      10.551  -5.366   7.025  1.00  0.33           N
ATOM    237  CA  SER A  29       9.897  -5.180   8.313  1.00  0.33           C
ATOM    238  C   SER A  29      10.200  -3.805   8.904  1.00  0.33           C
ATOM    239  O   SER A  29       9.880  -3.526  10.057  1.00  0.33           O
ATOM    240  CB  SER A  29      10.331  -6.266   9.348  1.00  0.33           C
ATOM    241  OG  SER A  29      11.754  -6.314   9.471  1.00  0.33           O
ATOM    242  N   LEU A  30      10.827  -2.897   8.117  1.00  0.34           N
ATOM    243  CA  LEU A  30      11.103  -1.516   8.497  1.00  0.34           C
ATOM    244  C   LEU A  30       9.830  -0.735   8.838  1.00  0.34           C
ATOM    245  O   LEU A  30       8.851  -0.743   8.093  1.00  0.34           O
ATOM    246  CB  LEU A  30      11.842  -0.773   7.356  1.00  0.34           C
ATOM    247  CG  LEU A  30      13.233  -1.311   6.948  1.00  0.34           C
ATOM    248  CD1 LEU A  30      13.605  -0.625   5.614  1.00  0.34
C
ATOM    249  CD2 LEU A  30      14.352  -1.428   8.018  1.00  0.34
C
ATOM    250  N   GLY A  31       9.814  -0.035   9.996  1.00  0.32           N
ATOM    251  CA  GLY A  31       8.612   0.642  10.497  1.00  0.32           C
ATOM    252  C   GLY A  31       8.333   1.985   9.885  1.00  0.32           C
ATOM    253  O   GLY A  31       7.291   2.588  10.161  1.00  0.32           O
ATOM    254  N   GLU A  32       9.262   2.491   9.068  1.00  0.32           N
ATOM    255  CA  GLU A  32       9.230   3.774   8.435  1.00  0.32           C
ATOM    256  C   GLU A  32       9.892   3.638   7.073  1.00  0.32           C
ATOM    257  O   GLU A  32      10.245   2.465   6.649  1.00  0.32           O
ATOM    258  CB  GLU A  32       9.899   4.876   9.337  1.00  0.32           C
ATOM    259  CG  GLU A  32      11.354   4.601   9.771  1.00  0.32           C
ATOM    260  CD  GLU A  32      11.900   5.633  10.762  1.00  0.32
C
ATOM    261  OE1 GLU A  32      11.164   6.591  11.109  1.00  0.32
O
```

```
ATOM    262  OE2 GLU A  32      13.071   5.438  11.181  1.00  0.32
O
ATOM    263  N   ALA A  33      10.124   4.671   6.292  1.00  0.46           N
ATOM    264  CA  ALA A  33      11.170   4.770   5.290  1.00  0.46           C
ATOM    265  C   ALA A  33      12.565   4.967   5.911  1.00  0.46           C
ATOM    266  O   ALA A  33      12.788   5.909   6.660  1.00  0.46           O
ATOM    267  CB  ALA A  33      10.897   5.974   4.375  1.00  0.46           C
ATOM    268  N   VAL A  34      13.558   4.096   5.611  1.00  0.45           N
ATOM    269  CA  VAL A  34      14.737   3.968   6.460  1.00  0.45           C
ATOM    270  C   VAL A  34      16.021   4.388   5.850  1.00  0.45           C
ATOM    271  O   VAL A  34      16.373   3.928   4.770  1.00  0.45           O
ATOM    272  CB  VAL A  34      14.852   2.542   6.923  1.00  0.45           C
ATOM    273  CG1 VAL A  34      16.140   2.277   7.768  1.00  0.45
C
ATOM    274  CG2 VAL A  34      13.542   2.441   7.749  1.00  0.45
C
ATOM    275  N   PHE A  35      16.774   5.242   6.582  1.00  0.39           N
ATOM    276  CA  PHE A  35      18.101   5.649   6.155  1.00  0.39           C
ATOM    277  C   PHE A  35      19.093   5.681   7.313  1.00  0.39           C
ATOM    278  O   PHE A  35      20.302   5.479   7.108  1.00  0.39           O
ATOM    279  CB  PHE A  35      18.086   7.032   5.462  1.00  0.39           C
ATOM    280  CG  PHE A  35      17.306   6.951   4.182  1.00  0.39           C
ATOM    281  CD1 PHE A  35      15.949   7.311   4.162  1.00  0.39           C
ATOM    282  CD2 PHE A  35      17.897   6.453   3.008  1.00  0.39           C
ATOM    283  CE1 PHE A  35      15.194   7.181   2.992  1.00  0.39           C
ATOM    284  CE2 PHE A  35      17.143   6.317   1.834  1.00  0.39           C
ATOM    285  CZ  PHE A  35      15.789   6.675   1.832  1.00  0.39           C
ATOM    286  N   ASP A  36      18.640   5.861   8.567  1.00  0.46           N
ATOM    287  CA  ASP A  36      19.470   5.867   9.768  1.00  0.46           C
ATOM    288  C   ASP A  36      20.159   4.529  10.019  1.00  0.46           C
ATOM    289  O   ASP A  36      21.322   4.450  10.422  1.00  0.46           O
ATOM    290  CB  ASP A  36      18.624   6.376  10.965  1.00  0.46           C
ATOM    291  CG  ASP A  36      18.354   7.871  10.786  1.00  0.46           C
ATOM    292  OD1 ASP A  36      18.928   8.478   9.838  1.00  0.46           O
ATOM    293  OD2 ASP A  36      17.586   8.423  11.608  1.00  0.46
O
ATOM    294  N   TYR A  37      19.462   3.436   9.668  1.00  0.35           N
ATOM    295  CA  TYR A  37      19.933   2.067   9.715  1.00  0.35           C
ATOM    296  C   TYR A  37      20.575   1.644   8.387  1.00  0.35           C
ATOM    297  O   TYR A  37      20.548   0.469   8.025  1.00  0.35           O
ATOM    298  CB  TYR A  37      18.768   1.090  10.031  1.00  0.35           C
ATOM    299  CG  TYR A  37      18.088   1.426  11.331  1.00  0.35
C
ATOM    300  CD1 TYR A  37      16.939   2.236  11.407  1.00  0.35
C
ATOM    301  CD2 TYR A  37      18.609   0.896  12.517  1.00  0.35
C
ATOM    302  CE1 TYR A  37      16.302   2.466  12.635  1.00  0.35
C
ATOM    303  CE2 TYR A  37      17.987   1.139  13.749  1.00  0.35
C
ATOM    304  CZ  TYR A  37      16.817   1.904  13.804  1.00  0.35           C
ATOM    305  OH  TYR A  37      16.145   2.115  15.023  1.00  0.35
O
ATOM    306  N   ILE A  38      21.153   2.590   7.606  1.00  0.42           N
ATOM    307  CA  ILE A  38      21.929   2.283   6.402  1.00  0.42           C
ATOM    308  C   ILE A  38      23.346   2.835   6.532  1.00  0.42           C
ATOM    309  O   ILE A  38      24.319   2.165   6.179  1.00  0.42           O
ATOM    310  CB  ILE A  38      21.273   2.835   5.130  1.00  0.42           C
ATOM    311  CG1 ILE A  38      19.838   2.268   4.998  1.00  0.42           C
ATOM    312  CG2 ILE A  38      22.126   2.505   3.878  1.00  0.42           C
ATOM    313  CD1 ILE A  38      19.093   2.723   3.741  1.00  0.42           C
ATOM    314  N   ARG A  39      23.513   4.043   7.122  1.00  0.41           N
ATOM    315  CA  ARG A  39      24.811   4.684   7.331  1.00  0.41           C
ATOM    316  C   ARG A  39      25.681   3.974   8.354  1.00  0.41           C
ATOM    317  O   ARG A  39      26.900   4.132   8.371  1.00  0.41           O
ATOM    318  CB  ARG A  39      24.637   6.134   7.842  1.00  0.41           C
ATOM    319  CG  ARG A  39      23.988   7.096   6.833  1.00  0.41           C
ATOM    320  CD  ARG A  39      23.823   8.498   7.424  1.00  0.41           C
ATOM    321  NE  ARG A  39      23.240   9.355   6.342  1.00  0.41           N
ATOM    322  CZ  ARG A  39      22.824  10.615   6.526  1.00  0.41
C
ATOM    323  NH1 ARG A  39      22.306  11.289   5.499  1.00  0.41
N
ATOM    324  NH2 ARG A  39      22.903  11.211   7.711  1.00  0.41
N
ATOM    325  N   SER A  40      25.078   3.178   9.249  1.00  0.39           N
ATOM    326  CA  SER A  40      25.818   2.354  10.181  1.00  0.39           C
ATOM    327  C   SER A  40      26.104   0.959   9.643  1.00  0.39           C
ATOM    328  O   SER A  40      26.960   0.258  10.182  1.00  0.39           O
ATOM    329  CB  SER A  40      25.012   2.194  11.496  1.00  0.39           C
ATOM    330  OG  SER A  40      23.705   1.666  11.235  1.00  0.39           O
ATOM    331  N   ILE A  41      25.409   0.520   8.570  1.00  0.38           N
ATOM    332  CA  ILE A  41      25.596  -0.808   7.995  1.00  0.38           C
ATOM    333  C   ILE A  41      26.688  -0.825   6.937  1.00  0.38           C
ATOM    334  O   ILE A  41      27.652  -1.589   7.039  1.00  0.38           O
ATOM    335  CB  ILE A  41      24.311  -1.302   7.321  1.00  0.38           C
ATOM    336  CG1 ILE A  41      23.103  -1.350   8.293  1.00  0.38           C
ATOM    337  CG2 ILE A  41      24.545  -2.659   6.610  1.00  0.38           C
ATOM    338  CD1 ILE A  41      23.220  -2.304   9.488  1.00  0.38           C
ATOM    339  N   TRP A  42      26.562  -0.009   5.867  1.00  0.31           N
ATOM    340  CA  TRP A  42      27.550   0.015   4.805  1.00  0.31           C
ATOM    341  C   TRP A  42      28.626   1.046   5.136  1.00  0.31           C
ATOM    342  O   TRP A  42      29.814   0.727   5.205  1.00  0.31           O
ATOM    343  CB  TRP A  42      26.831   0.302   3.452  1.00  0.31           C
ATOM    344  CG  TRP A  42      27.743   0.380   2.238  1.00  0.31           C
ATOM    345  CD1 TRP A  42      28.333   1.488   1.700  1.00  0.31           C
ATOM    346  CD2 TRP A  42      28.278  -0.748   1.529  1.00  0.31           C
ATOM    347  NE1 TRP A  42      29.201   1.134   0.685  1.00  0.31           N
ATOM    348  CE2 TRP A  42      29.198  -0.248   0.587  1.00  0.31           C
ATOM    349  CE3 TRP A  42      28.072  -2.116   1.682  1.00  0.31           C
ATOM    350  CZ2 TRP A  42      29.928  -1.114  -0.222  1.00  0.31           C
ATOM    351  CZ3 TRP A  42      28.766  -2.983   0.829  1.00  0.31           C
ATOM    352  CH2 TRP A  42      29.677  -2.493  -0.114  1.00  0.31
C
ATOM    353  N   GLY A  43      28.196   2.294   5.397  1.00  0.42           N
ATOM    354  CA  GLY A  43      29.042   3.445   5.670  1.00  0.42           C
ATOM    355  C   GLY A  43      28.745   4.566   4.655  1.00  0.42           C
ATOM    356  O   GLY A  43      27.815   4.395   3.814  1.00  0.42           O
ATOM    357  OXT GLY A  43      29.431   5.621   4.728  1.00  0.42
O
TER     358      GLY A  43
END
```

B)

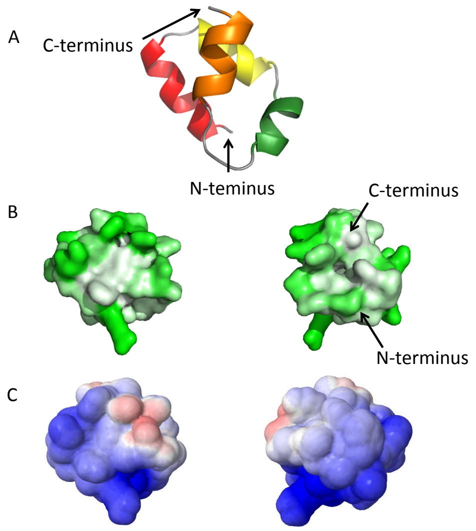

Figure S6- Structural models of mutacin BhtB. A) Ribbon diagram depicting the saposin-like fold. The C- and N- termini are explicitly shown. Hydrophobic surface (B) and electrostatic potential surface (C) maps of mutacin BhtB. Orientation peptide backbone in surface filling diagrams, B and C, correlate to one another.

References:

84 M. Biasini, S. Bienert, A. Waterhouse, K. Arnold, G. Studer, T. Schmidt, F. Kiefer, T. G. Cassarino, M. Bertoni, L. Bordoli and T. Schwede, *Nucleic Acids Res.*, 2014, **42**, 252–258.

85 H. M. Berman, J. Westbrook, Z. Feng, G. Gilliland, T. N. Bhat, H. Weissig, I. N. Shindyalov and P. E. Bourne, *Nucleic Acids Res.*, 2000, 28, 235–242.
